# Diversity and Screening of Cellulolytic Microorganisms from Mangrove Forests, Natural Parks, Paddy Field, and Sugarcane Plantation in Panay Island, Philippines

**DOI:** 10.1155/2024/5573158

**Published:** 2024-07-23

**Authors:** Isabel Grace T. Gatpatan, Rhudith B. Cabulong, Resurreccion B. Sadaba

**Affiliations:** Division of Biological Sciences College of Arts and Sciences University of the Philippines Visayas, Miagao, Iloilo 5023, Philippines

## Abstract

Cellulolytic microorganisms secrete cellulase, which plays a crucial role in the conversion of lignocellulosic biomass into value-added products with diverse applications in industries, such as biofuel, healthcare, and agriculture. As the world transitions to a bioenergy future, cellulase demand is likely to expand. However, the high cost and low catalytic activity of cellulase hinder the commercialization of biorefineries. Searching for cellulase-producing microorganisms in different environments through bioprospecting can aid in broadening the range of cellulases that are currently available. Meanwhile, the cellulolytic activity of marine microorganisms remains largely unexplored, making it difficult to compare the cellulolytic activity of terrestrial and marine environments. Thus, this study aimed to investigate the diversity and activity of culturable cellulolytic microorganisms in four terrestrial and three marine sites within Panay Island, Philippines. The results showed that the cellulolytic microbial load was tenfold higher in the terrestrial sites than in the marine sites, possibly due to the dynamic mangrove environment. Out of the 42 isolates with a high cellulolytic index (CI) of ≥3.0, 36 were from terrestrial and 6 from marine habitats. The CMCase, Avicelase, and FPase activities were then tested on the 18 isolates with the highest CI. It was observed that many isolates had a high CI, but few exhibited high enzyme activities. Marine isolates showed higher CMCase and Avicelase activities, with comparable FPase activity to their terrestrial counterparts. Isolates S1ACP6B from a sugarcane field and MS1OMP2A from a mangrove site exhibited the highest cellulase activities at 0.41 and 0.29 U/mg, respectively, and were identified as *Enterobacter roggenkampii* and *Rhodococcus erythropolis*, respectively. Among the 18 identified isolates, three are resistant to chloramphenicol and three isolates are potentially new species of *Halomonas* sp. MS1ACP1B, *Albirhodobacter* sp. MP2ACP3B, and *Saccharomycetaceae* sp. B1CZP10A. Overall, this study provides an insight on the composition of cellulolytic microbial load and their activities among various habitats.

## 1. Introduction

Oil and energy markets are essential to the functioning of the economy. However, the use of fossil fuels such as coal, oil, and natural gas has significant environmental and health costs. Increasing fossil fuel consumption contributes to the global increase in carbon footprint. With this continuing economic and environmental problem, many researchers around the world have undertaken the challenge of finding solutions by employing new and innovative technologies, such as the conversion of renewable and sustainable biomass into building blocks of polymers and other materials. Bioconversion minimizes carbon emissions and employs inexpensive feedstocks that do not compete with food production and consumption. Recently, the bioconversion of lignocellulosic biomass has gained popularity due to its potential as a raw material for bioprocesses, which could ameliorate the global energy, food, and climate crises.

Lignocellulosic biomass can be used industrially, but it must first be cleaved into its lignin, hemicellulose, and cellulose-building units. This natural process is made possible in nature by cellulolytic microorganisms, such as bacteria, fungi, and actinomycetes. Cellulolytic microorganisms play a vital role in the global carbon cycle by secreting cellulase. They regulate the dynamics of soil organic matter, carbon sequestration, greenhouse gas emissions, and other aspects of nutrient cycling [1]. However, a comprehensive and comparative study on the cellulolytic microorganisms present in different environments, especially marine environments, remains unexplored and needs further elucidation.

Endoglucanases, cellobiohydrolase (also known as exoglucanase), and *β*-glucosidase make up the cellulase enzymes [2]. Due to its widespread use in industries such as food production, agriculture, biofuel, textiles, and healthcare, cellulase has a growing commercial market demand [3]. However, the high cost of cellulase and its low catalytic efficiency have impeded the commercialization of biomass-based manufacturing industries [4, 5]. This means that more research is needed to lower the cost of making cellulase and identify new cellulolytic microorganisms with better catalytic efficiencies. Exploring diverse environments for cellulase-producing microorganisms through bioprospecting offers the potential to broaden the repertoire of available cellulases. Moreover, there is a significant demand for cellulases that possess inherent capabilities to function in extreme environments, meeting the demand of modern biotechnological industries. Marine-derived cellulases are examples of enzymes that can withstand extreme conditions, crucial for the hydrolysis of seaweed and other marine biomass applicable to the marine (blue) bioeconomy. Despite their potential, only a few marine-derived cellulases have been investigated for their biotechnological applications.

The Philippines' archipelagic topography offers a variety of conditions conducive to supporting biodiversity. The country is recognized as a biodiversity hotspot due to a strong correlation between microbial species richness and attributes, such as habitat availability, heterogeneity, and surface temperature [6]. Consequently, this makes the Philippines an attractive reservoir for discovering novel cellulases from both the marine and terrestrial environments. However, the diversity and screening of cellulolytic microorganisms in the country remains underexplored.

The current study presents the enumeration, isolation, characterization, and identification of cellulolytic microorganisms derived from mangrove sediments and terrestrial soils on the Philippine Island of Panay. The terrestrial sites exhibit a tenfold higher cellulolytic microbial load than the marine sites, suggesting the influence of the dynamic mangrove environment. This provides valuable insight on the understudied environmental factors that may influence the abundance and distribution of cellulolytic microorganisms existing in various habitats. While the microbial ecology of cellulose degradation in bovine rumen and termites is well understood and researched, there much remains to be elucidated on these cellulolytic microorganisms present from different environments, particularly the marine environments [7]. In addition, marine cellulolytic microorganisms remain understudied, and this current study is among the limited number of studies that have compared cellulolytic microorganisms present in both terrestrial and marine environments.

The isolated cellulolytic microorganisms were characterized and compared based on the zone of clearing on CMC agar plate and on their enzymatic activities. Marine isolates displayed significantly higher CMCase and Avicelase activities than their terrestrial counterparts, with comparable FPase enzyme activity. *Enterobacter roggenkampii* and *Rhodococcus erythropolis* were identified as the highest cellulase producers among the isolates from terrestrial and marine environments, respectively. In addition, three isolates are resistant to chloramphenicol, suggesting potential applications in antibiotic production and other secondary metabolites. Lastly, three isolates are potentially new species of cellulolytic microorganisms, which need further elucidation of their genetic makeup and physiological characteristics.

## 2. Materials and Methods

### 2.1. Collection of Soil Samples

**Figure 1:**
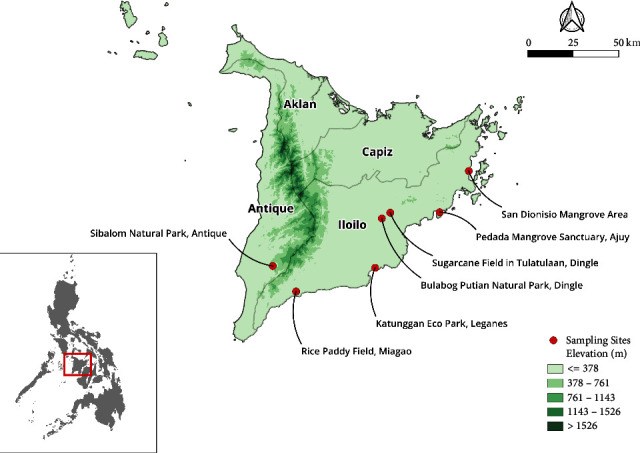
Map of sample collection sites (drawn using QGIS version 3.34). Panay Island is divided into four provinces—namely, Iloilo, Antique, Capiz, and Aklan—all situated within the Western Visayas region of the Philippines.

The seven chosen sites for soil sampling were (1) Bulabog Putian National Park of Dingle, Iloilo; (2) Sibalom Natural Park of Antique; (3) a mangrove forest located at San Dionisio, Concepcion, Iloilo; (4) Pedada Mangrove Sanctuary in Ajuy, Iloilo; (5) Katunggan Eco-Park in Leganes, Iloilo City; (6) a rice paddy field in Brgy. Malagyan, Miagao, Iloilo; and (7) a sugarcane plantation in Brgy. Tulatula-an, Dingle, Iloilo, due to the different geographical features and environmental conditions (Figure 1). The terrestrial sites are the Bulabog Putian National Park, a protected wildlife and natural park located between the town of Dingle and San Enrique, Iloilo province in Panay Island with an elevation of 112 meters and an area of 854.33 hectares along a 40 kilometers trail; Sibalom National Park, another protected area located at Barangay Imparayan, Sibalom, Antique province with an elevation of 134 meters, an area of 5,511.47 hectares, and considered as one of the last lowland rainforests within Panay Island; the rice paddy field, located at Barangay Malagyan, Miagao, Iloilo, with an elevation of 29 meters, an estimated area of 2 hectares, and owned by a local resident; and the sugarcane field, located at Barangay Tulatula-an, Dingle, Iloilo, with an elevation of 84 meters, an estimated area of 1 hectare, and likewise owned by a local resident. On the other hand, the marine sites are the Pedada Mangrove Sanctuary, a mangrove eco-park located at Barangay Pedada, Ajuy, Iloilo, with an elevation of 3 meters, approximately 300 meters along the coastline, and managed by the Barangay authorities; the San Dionisio Mangrove area located at Concepcion—San Dionisio Road, Iloilo, with an elevation of 18 meters and observed to be within residential area; and the Leganes Integrated Katunggan Eco-Park located at Barangay Gua-an, Leganes, Iloilo, with an elevation of 10 meters and an area of 15 hectares of reforested mangrove along the coastline of Leganes.

Diverse microbial communities are most prevalent in the O (organic) and A (subsurface) horizons between 0 and 15 cm from the surface [8]. Consequently, 100 g soil samples were randomly collected from three organic-rich spots roughly 100 ft apart on each site, then combined in one Whirl-Pak sterile sampling bag to create a composite sample. All collected soils were transported to the Soils Laboratory of the Department of Agriculture Region VI for pH and soil chemical analyses. The remaining samples were brought back to the microbiology lab for the isolation of desired microorganisms. Prior to soil collection, soil temperature and pH were measured, as well as the elevation, coordinates, and relative humidity of the site.

### 2.2. Enumeration and Isolation of Cellulolytic Microorganisms

The enumeration of cellulolytic microorganisms was assessed using a conventional serial dilution technique and spread plating. Omeliansky's medium (OM; composition: 1 g/L (NH₄)₂SO₄, 1 g/L K_2_HPO_4_ (anhydrous basis), 0.5 g/L MgSO_4_·7 H_2_O, 2 g/L CaCO_3_, 0.1 g/L NaCl), Czapek's medium (CZ; composition: 2 g/L NaNO_3_, 0.5 g/L KCl, 1.5 g/L MgSO_4_·7 H_2_O, 1.0 g/L K_2_HPO_4_, 0.01 g/L FeSO_4_), and Shirling and Gottlieb modified medium (AC; composition: 1 g/L K_2_HPO_4_, 1 g/L MgSO_4_·7 H_2_O, 1 g/L NaCl, 2 g/L (NH₄)₂SO₄, 0.01 g/L FeSO_4_) were used for the cultivation of bacteria, fungi, and actinomycetes, respectively [9, 10]. Each medium contained 10 g/L carboxymethyl cellulose (CMC) powder as the sole carbon source and was supplemented with either chloramphenicol (35 *µ*g/ml) or nystatin (100 U/mL) to prevent unwanted growth. For marine-derived microorganisms, 20 g/L of sodium chloride was added to the agar medium. After seven days of incubation at 30°C, colonies were counted and expressed as colony-forming units (CFU) per gram of sample. Subsequently, distinct colonies were picked and restreaked on fresh media. The isolates were then characterized further by streaking them on agar plates containing either 10 g/L of cellulose or 10 g/L of rice husk as the sole carbon source. Isolates that grew on either cellulose or rice husk after seven days of incubation were recorded, and glycerol stocks were prepared.

### 2.3. Quantitative Screening of Cellulolytic Microorganisms

Cell cultures were grown for a period of one to three days until the optical density at 600 nm (OD_600_) reached approximately 0.3. These cultures were prepared using the well-isolated colonies from the initial screening and subsequently spot-plated onto modified AC agar plates (MAC). The MAC medium comprised of 1 g/L K_2_HPO_4_ (anhydrous basis), 1 g/L MgSO_4_·7 H_2_O, 1 g/L NaCl, 1 g/L (NH₄)₂SO₄, 0.01 g/L FeSO_4_, 0.3 g/L peptone, and 0.3 g/L yeast extract with 5 g/L CMC as the sole carbon source. After 72 hours of incubation at 30°C, the plates were flooded with 1% Gram's iodine solution. All experiments were performed in triplicates. Positive colonies exhibiting a zone of clearing indicate that the CMC was utilized. The diameter of the zone of clearing was then measured and compared for each microorganism, and the cellulolytic index (CI) was calculated using the formula as follows.

(1) $$\begin{array}{c} \begin{array}{cc} \; & \text{Cellulolytic Index}\left(\text{CI}\right)\\ \; & =\frac{\text{Diameter of clearance zone}-\text{diameter of bacterial colony}}{\text{diameter of bacterial colony}}. \end{array} \end{array}$$

### 2.4. Enzyme Activity Assays

Enzyme activities on CMC, Avicel, and filter paper were tested using the 3,5-dinitrosalicylic acid (DNS) method, as described by Liang et al. (2014) [11]. Briefly, microorganisms were cultivated in MAC broth containing 5 g/L cellulose and harvested after 72 hours. The resulting supernatants served as crude enzymes for subsequent enzyme assays. The crude enzyme concentration was measured at 280 nm using a UV-Vis spectrophotometer [12]. To measure CMCase, Avicelase, and FPase activities, 200 *µ*L of crude enzyme was mixed with 0.2% (w/v) CMC, Avicel, or Whatman number 1 filter paper, respectively. The mixtures were then incubated for 30 minutes at 50°C [13]. To stop the reaction, 3 mL DNS reagent was added, and the mixture was heated in boiling water for 5 minutes until color development was observed. Prior to the reading of absorbance, the mixture was placed in an ice bath. The absorbance was then measured at 540 nm [14]. Using glucose as a standard, absorbance values were recorded and the amount of reducing sugar was calculated. Under the assay conditions, one unit (U) of cellulase activity is defined as the amount of cellulase required to produce 1 *µ*mol of reducing ends per minute. All assays were performed in triplicates.

### 2.5. Optimization of pH and Temperature for Optimum Growth and Cellulase Production

The effects of temperature and pH on enzyme production were determined in this study. Two isolates, S1ACP6B (a terrestrial isolate) and MS1OMP2A (a marine isolate), which exhibited the highest cellulase activity during the initial enzyme activity assay, were grown in MAC agar supplemented with 5 g/L CMC as their carbon source and appropriate antibiotics, and with varying pH of 4.5, 5.5, 6.5, 7.5, and 8.5. The plates were incubated for 72 hours at 30°C. The pH value that yielded the highest CI was selected for subsequent agar preparation. Similarly, the isolates were incubated at different temperatures of 25, 30, 37, 42, and 50°C, and CIs were measured. Using the optimum pH and temperature, the two isolates were then cultured in a MAC medium broth containing 5 g/L cellulose, and enzyme activity was measured using the DNS method as described previously. All experiments were performed in triplicates.

### 2.6. Molecular Identification of Cellulolytic Microorganisms

Eighteen cellulolytic microorganisms that showed the highest CIs were identified using molecular biology techniques. The genomic DNAs (gDNAs) of the isolates were extracted and purified at the Philippine Genome Center (PGC) Visayas using QIAGEN DNeasy blood and tissue extraction kit for fungal isolates and the Vivantis Bacterial DNA Extraction kit for bacterial isolates. The extracted gDNA then served as template for the amplification of 16S rRNA and ITS1-5.8S-ITS2 rRNA regions in bacteria and fungi, respectively. The primers used for amplification were 27F (5′ AGAGTTTGATCMTGGCTCAG 3′) and 1492R (5′ TACGGYTACCTTGTTACGACTT 3′) for bacteria and actinomycetes, and ITS-1F (5′ CTTGGTCATTTAGAGGAAGTAA 3′) and ITS-4 (5′ TCCTCCGCTTATTGATATGC 3′) for fungi. The amplified and purified 16S rRNA and ITS regions were then sequenced by PGC Visayas. The clean sequence results were then compared and analyzed using NCBI Microbial Nucleotide BLAST (https://blast.ncbi.nlm.nih.gov/Blast.cgi?PAGE_TYPE=BlastSearch&BLAST_SPEC=MicrobialGenomes) and NCBI Standard Nucleotide BLAST (https://blast.ncbi.nlm.nih.gov/Blast.cgi?PROGRAM=blastn&BLAST_SPEC=GeoBlast&PAGE_TYPE=BlastSearch) online. Multiple alignment analysis was performed using ClustalW, and phylogenetic tree was constructed with a bootstrap value of 1000 using MEGA 11 software and further visualized using iTOL online [15]. Lastly, the partial 16S rRNA sequences of the potentially new species were deposited in GenBank with accession numbers PP748307, PP748306, and PP748254 for *Saccharomycetaceae* sp. B1CZP10A, *Albirhodobacter* sp. MP2ACP3B, and *Halomonas* sp. MS1ACP1B, respectively (Table S6).

### 2.7. Biochemical Characterization of Possible Novel Isolates

The cultural and morphological properties of the isolates, including color, form, elevation, and margin, were examined by colonies grown in nutrient agar (NA) medium for bacteria/actinomycetes and potato dextrose agar (PDA) medium for fungi. Bacterial isolates were gram-stained, while the fungal isolate was stained with lactophenol cotton blue (LPCB) and viewed using a light microscope. The physiological and biochemical characterization of the isolates was carried out following the standard protocols [16]. The biochemical tests were as follows: IMViC test, catalase, hydrolyzing ability on casein, starch, urea, and gelatin, and different carbohydrates.

### 2.8. Statistical Analysis

Statistical analyses were performed using ANOVA and Tukey's test. Minitab and Jamovi software types were used to determine the significant difference between variables [17, 18]. Only enzyme assay and cellulolytic index results were subjected to statistical analysis.

## 3. Results and Discussion

### 3.1. Abundance of Cellulolytic Microorganisms Varied Significantly between Terrestrial and Mangrove Soil

Cellulolytic microorganisms are important in organic matter degradation in the environment. They degrade cellulose, which is a recalcitrant and a major plant component, with the cellulase enzyme they produce. They are also important in the bioprocess of converting biomass to valuable chemicals. Enumeration of cellulolytic microorganisms in various environments would help in discovering novel cellulases that may have new applications. It would also help in improving our understanding of the cellulolytic activities present in various and across different environments. However, only a few studies have compared the cellulolytic activities present in both terrestrial and marine habitats. The cellulolytic activity of marine microorganisms remains largely unexplored, making it difficult to compare the cellulolytic activity of terrestrial and marine environments. In this study, several cellulolytic microorganisms from the different locations in Panay Island, Philippines, were enumerated and compared.

**Figure 2:**
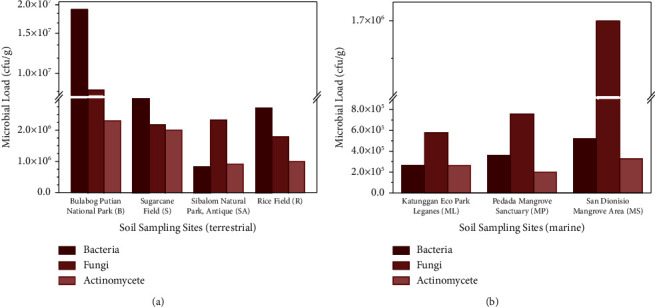
A comparison of the number of cellulolytic microorganisms present at various (a) terrestrial and (b) mangrove sites in Panay Island.

The abundance and composition of culturable cellulolytic microorganisms, including bacteria, fungi, and actinomycetes, were found to differ between terrestrial and mangrove soil habitats (Figure 2). The microbial count in the terrestrial soil (10^6^ cfu/g) was approximately ten times higher than that in the mangrove soil (10^5^ cfu/g). Moreover, the microbial community structure varied between the two habitats. In the terrestrial soil samples, bacterial populations were found to be dominant, followed by fungi and actinomycetes. In contrast, the mangrove soil samples revealed a higher abundance of fungi, followed by bacteria and actinomycetes. The observed differences in microbial abundance and community structure between the habitats can potentially be attributed to the contrasting environmental conditions. The terrestrial environment provides relatively stable soil structure, which facilitates microbial growth and, in turn, enhances the soil fertility and productivity, whereas the mangrove environment is characterized by frequent inundation and dynamic soil conditions, which may be less favorable for microbial growth. The frequent inundation in the mangrove areas leads to an inconsistent availability of nutrients and organic matter essential for sustained microbial activity, in contrast to the steady conditions found in terrestrial environments. Generally, the collected mangrove soils exhibited low total organic matter and nitrogen as compared to terrestrial areas (Table S1).

Among the examined soil samples, Bulabog Putian National Park in Dingle, Iloilo, had the highest cellulolytic microbial counts for bacteria (1.91 × 10^7^ cfu/g), fungi (8.47 × 10^6^ cfu/g), and actinomycetes (2.30 × 10^6^ cfu/g) (Figure 2(a)). This may be attributed to the park's natural and undisturbed environment, which allows for a rich plant diversity, including native plant species flourishing in the area. It is known that plant diversity can have a significant effect on soil carbon storage and, therefore, on microbial diversity as well [19]. The soil collected from the sugarcane field in the same municipality had the second-highest microbial load (bacteria: 4.28 × 10^6^ cfu/g; fungi: 2.17 × 10^6^ cfu/g; actinomycetes: 1.99 × 10^6^ cfu/g), followed by the rice field in Miagao, Iloilo (bacteria: 2.71 × 10^6^ cfu/g; fungi: 1.78 × 10^6^ cfu/g; actinomycetes: 9.94 × 10^5^ cfu/g), and Sibalom Natural Park in Antique (bacteria: 8.37 × 10^5^ cfu/g; fungi: 2.33 × 10^6^ cfu/g; actinomycetes: 9.10 × 10^5^ cfu/g).

Results showed that bacterial isolates predominate in the terrestrial soils except for the soils collected from Sibalom Natural Park, Antique, where the fungal load was the highest. This may be due to the area of collection of soil samples within the reforested area in Sibalom where mahogany is the dominant tree species. The low microbial load in Sibalom soil samples may be attributed to its low levels of total nitrogen and organic matter compared to Bulabog Putian Natural Park (Table S1). Both the Sibalom Natural Park and the Bulabog Putian Natural Park are protected reserve areas in Panay Island, and the differences in microbial abundance could be attributed to variations in the soil properties and vegetation cover in each location. Furthermore, the abundance of fungi may be due to the presence of phytochemical tannin in the mahogany trees, which could exert an allelopathic effect on their surroundings. Tannins have been shown to have antibacterial activity and are inherently weak acids, potentially leading to the slight acidity of soil (pH 5.33–6.27) [20]. This acidic soil condition may have contributed to the proliferation of fungi, which are known to thrive in undisturbed and slightly acidic environment [21]. Another probable explanation is the prevalence of monoculture in that specific area within Sibalom Natural Park. Continuous monoculture has been observed to limit the growth and development of crops and other vegetation as well as the growth of microorganisms, in which principles could also be applied in forest areas [22]. In general, bacteria are the most common type of microorganisms found on land, but Sibalom Natural Park soil showed otherwise; hence, more research needs to be done to fully understand microbial communities on these areas. Metagenomic analysis may provide insights on the number and type of cellulolytic microorganisms present in terrestrial habitats with and without invasive plant species, such as the mahogany tree. This analysis could significantly contribute to our understanding of how invasive plant species affect microbial and soil properties within an ecosystem and could inform better management strategies for these ecosystems. It may also help in understanding the interactions between cellulolytic microorganisms and other soil microorganisms.

Among mangrove sites, samples from San Dionisio mangrove area showed the highest number of cellulolytic microorganisms for bacteria (5.20 × 10^5^ cfu/g), fungi (1.68 × 10^6^ cfu/g), and actinomycetes (3.30 × 10^5^ cfu/g). This is then followed by Pedada Mangrove Sanctuary (bacteria: 3.60 × 10^5^ cfu/g; fungi: 7.57 × 10^5^ cfu/g; actinomycetes: 2.00 × 10^5^ cfu/g) and Leganes Integrated Katunggan Eco-Park (bacteria: 2.65 × 10^5^ cfu/g; fungi: 5.80 × 10^5^ cfu/g; actinomycetes: 2.60 × 10^5^ cfu/g), respectively (Figure 2(b)). Results showed that the fungal diversity is relatively high in mangrove areas, which may be attributed to the generally limited nutrients found in mangrove environments as compared to terrestrial environments (Table S1), restricting the growth of other microorganisms. Tannins from mangrove trees, such as *Sonneratia alba* and *Bruguiera cylindrica*, may have also leached into mangrove soils, contributing to another plausible explanation [23, 24]. It is noteworthy that there is a similarity in the microbial load count observed in the mangrove areas and Sibalom Park, wherein fungal counts are higher and tannins are possibly present. Lastly, the remarkable adaptability and tolerance of fungi to harsher and more dynamic environments, such as mangroves, may also have contributed to the high fungal load [25].

### 3.2. Screening of Cellulolytic Microorganisms

Among the 340 distinct colonies that were picked, 183 isolates grew on agar plates containing cellulose or rice husk as the sole carbon source (Figures S1 and S2). Despite being considered a waste product from rice milling and often being dumped or burned, rice husk is an inexpensive and easily accessible source of lignocellulosic biomass that could be used in biorefinery processes. It is possible that the isolates that displayed growth on rice husk could also breakdown the recalcitrant lignin component. To disrupt the recalcitrant structure of rice husk, physical and/or chemical pretreatment or a combination of these processes is required [26, 27]. In this study, the degradation of rice husk by these cellulolytic microbes may be attributed to the milling and sterilization of rice husk during media preparation. Interestingly, even the isolated marine microorganisms also demonstrated growth on rice husk, indicating the potential of halophiles for biorefinery applications.

Out of the 183 isolates that grew on agar plates with cellulose or rice husk as the sole carbon source, only 42 had a high cellulolytic index (CI) greater than or equal to 3.0 (Figure S3). Of these 42, 36 were from terrestrial habitats and 6 were from marine habitats. The sugarcane field in Dingle yielded the highest number of terrestrial isolates with a high CI of greater than 3.0, followed by Sibalom Natural Park in Antique, Bulabog Putian, and Rice Paddy Field in Miagao, respectively. In comparison with bacteria and fungi, terrestrial actinomycetes were found to have a high CI. On the other hand, the San Dionisio mangrove area and Katunggan Eco-Park yielded the highest number of marine isolates with a high CI of greater than 3.0, followed by the Pedada Mangrove Sanctuary. Among all isolates, the isolate S2CZP6B from the sugarcane field displayed the highest CI of 6.7 ± 1.59, while ML1OMP2A from Katunggan Eco-Park, Leganes, had the highest CI of 4.5 ± 1.53 among the marine isolates (Table 1). This suggests that these isolates may produce a substantial amount of cellulase due to the positive correlation between hydrolysis capability (development of CI) and the cellulase production potential.

### 3.3. Enzymatic Activities of the Cellulolytic Microorganisms

To confirm the cellulase activities, the top 18 isolates with the highest CI from the 42 isolates were selected and evaluated for CMCase, Avicelase, and FPase activities. The degradation of CMC, which is a soluble cellulose derivative, was used to evaluate the endoglucanase activity of the microorganisms (CMCase). Avicel, which is an insoluble microcrystalline cellulose that mimics pretreated biomass in industrial settings, was used to measure the exoglucanase activity (Avicelase) [28]. Lastly, the degradation of filter paper, which is similar to cellulose powder, was used to measure the total cellulase activity (FPase) [29]. The results showed that the enzyme assays and the CIs of the various isolates were significantly different from each other (*p* < 0.05) (Table S2). In terms of CMCase, marine isolates MS1OMP2A and MS1ACP1B displayed the highest activities, measuring 0.279 ± 0.01 and 0.281 ± 0.05 U/mg, respectively, among all tested isolates (Table 1). On the other hand, in the terrestrial environment, the B2OMP3A isolate exhibited the highest CMCase activity of 0.246 ± 0.02 U/mg. As for Avicelase activity, marine isolate MS1ACP1B demonstrated the highest activity at 0.370 ± 0.11 U/mg among all the isolates, while isolate S1ACP2C held the top position among the terrestrial isolates with 0.242 ± 0.08 U/mg. In terms of FPase, terrestrial isolate S1ACP6B showed the highest activity of 0.390 ± 0.01 U/mg among all tested isolates, whereas isolates MS1OMP2A and ML2OMP1B exhibited the highest activities of 0.265 ± 0.04 and 0.237 ± 0.01 U/mg, respectively, among marine isolates. Finally, for CIs, terrestrial isolate S2CZP6B had the highest activity at 6.70 ± 0.00 among all the isolates, while ML1OMP2A and MS1OMP2A showed activities at 4.50 ± 0.00 and 4.40 ± 0.08, respectively, the highest CI activities among the marine isolates.

The overall enzyme and CI activities were likewise evaluated in both terrestrial and marine environments. The analysis revealed a noteworthy significant difference in CMCase, Avicelase, and CIs (*p* < 0.05) between the two environments (Table S3). In general, marine isolates exhibited considerably higher CMCase and Avicelase activities in comparison with terrestrial isolates, whereas terrestrial isolates displayed higher CIs (Table S4). However, there were no significant differences (*p*=0.66) observed between the two environments in terms of their FPase activities. These results suggest that marine enzymes can contend well with those from terrestrial origins.

Marine microorganisms have distinct genetic structures and life habitats that set them apart from terrestrial environments [34]. The complexity of the marine environment, characterized by highly variable conditions such as high salinity, high pressure, low temperature, and varying lighting conditions, contributes to the notable differences in the enzymes produced by marine microorganisms [35]. The marine environment encompasses both nutrient-rich and nutrient-deficient areas, creating niches where only a few species can thrive.

Enzymes produced by marine microorganisms, especially those in extreme areas, are believed to possess distinctive pressure-adaptation mechanisms suitable for industrial applications. This is particularly valuable in industries where increased stability and activity are crucial to withstand a variety of conditions. Marine cellulases exhibit high specific activity, thermostability in extreme conditions, and other unique biochemical properties. Despite these characteristics, very few have been studied for the biotechnological potential of marine cellulases [36]. These enzymes play a crucial role in hydrolyzing marine biomass, such as seaweeds, contributing to the emergence of marine cellulosic (blue) biotechnology. This emerging field utilizes marine resources to their full potentials for enhanced applications in medical, pharmaceutical, food, fuel, and agricultural sectors. Furthermore, investigations into cellulose degradation in saline environments may unveil novel metabolic pathways and enzymes with well-defined catalytic properties [37].

In a recent study, cellulolytic marine bacteria were isolated from seagrass sediments with the aim of hydrolyzing sugarcane bagasse and reducing the dependence on costly carbon sources in shrimp aquaculture [38]. Another study highlighted the capabilities of some marine bacterial isolates in degrading lignin-derived compounds, such as ferulic acid, benzoic acid, and 4-hydroxybenzoic acid. These microbial marine isolates were identified as members of the genera *Halomonas, Arthrobacter, Pseudoalteromonas, Marinomonas*, and *Thalassospira *[39]. These studies underscore the potential of marine microbial resources for cellulosic biotechnology. It is anticipated that there will be a noticeable increase in the application of marine microbial enzyme technology in the years to come.

The results of the enzyme assays showed that some isolates exhibited high total cellulase activities despite having low endoglucanase and exoglucanase activities, while others exhibited high endoglucanase and exoglucanase activities but low total cellulase activities. The presence of low endoglucanase and/or exoglucanase activities but high total cellulase activities could indicate that there is a synergistic relationship among the component enzymes, and all are necessary to achieve optimal cellulase activities. Conversely, a low total cellulase activity with high individual component activities may have several explanations. For instance, the microorganism may prefer a less complex substrate as CMC and Avicel over a more complex filter paper. Alternatively, the production of *β*-glucosidase enzyme by the microorganism may be insufficient compared to the production of endoglucanase and exoglucanase, resulting in a low total cellulase activity.

Additionally, the results of the enzyme assay revealed that the isolates have comparatively low activities compared to those reported in the literature [40]. Although isolates S2CZP6B and ML1OMP2A showed the highest CI among the terrestrial and marine isolates, they did not exhibit high enzyme activities. This suggests that there is no significant correlation between the CI and the cellulase activity of the isolates, similar to the previous findings of Liang et al. (2014). The absence of detectable cellulase activities in the broth medium could be due to the low concentration of enzymes produced, the inability of the isolates to secrete enzymes, or inactivation of enzymes due to the 50°C temperature used in the assays. It is also plausible that the enzymes have better activities in solid medium rather than in a liquid medium. Assessing cellulase activity against commercial substrates such as CMC may also not precisely indicate activity against the natural lignocellulosic materials. Lucena et al. highlighted the significant difference between using natural substrates versus commercial cellulase substrates to evaluate enzyme hydrolytic activities from Brazilian termites [41]. In addition, the diameter of zone of clearance was also reported to be an unreliable indicator of cellulase activity for it may not accurately reflect the actual enzyme activity of the microorganisms [42]. Isolates MS1OMP2A and S1ACP6B, which showed high total cellulase activities, were then used for subsequent experiments.

### 3.4. Optimization of pH and Temperature

**Figure 3:**
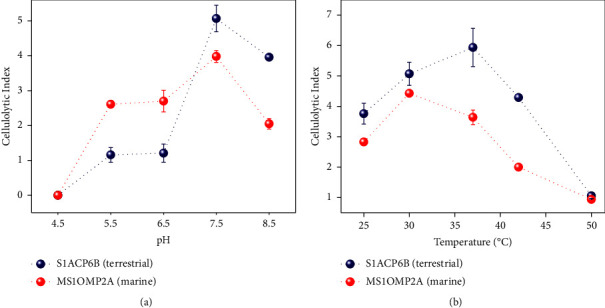
The optimization of (a) pH and (b) temperature of S1ACP6B and MS1OMP2A isolates grown on CMC agar medium.

Optimization of the different enzyme production parameters is important for commercial applications. In assaying enzymes, the crucial factors to be considered are temperature, pH, ionic strength, and the appropriate concentration of key components like substrates and enzymes [43]. pH and temperature serve as important control factors in the fermentative process, ensuring optimal microbial growth, enzyme activity, and product formation [44]. Hence, in this study, the optimal pH and temperature for the growth of two isolates, namely, S1ACP6B from a sugarcane field and MS1OMP2A from San Dionisio mangrove site, which exhibited the highest FPase or total cellulase activity, were determined. The two isolates were first grown on agar with varying pH values, and their CIs were measured. The results showed that both isolates exhibited optimal growth at pH 7.5, with CI values of 5.0 ± 0.46 and 4.0 ± 1.45, respectively (Figure 3(a)). The CI values at pH 7.5 were either greater than or comparable to the values before optimization, indicating that neutral pH is favorable for optimal growth. The findings are consistent with previous studies that have also shown that several cellulolytic bacterial species exhibit good growth at pH between 7.0 and 7.5 [45, 46].

**Figure 4:**
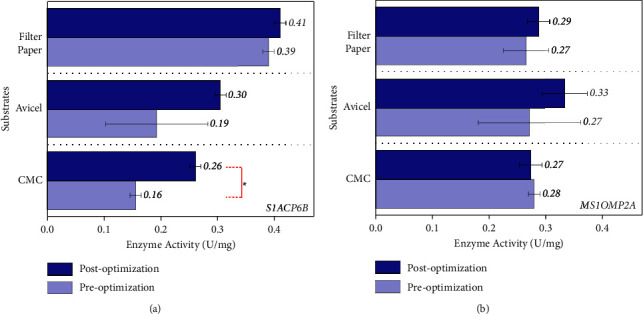
The enzyme activities of the (a) terrestrial isolate S1ACP6B and (b) marine isolate MS1OMP2A before and after optimization of medium pH and incubation temperature. ^*∗*^Significantly different (*p* < 0.05).

After optimizing the pH, the incubation temperature was also optimized, and the results showed that the cellulolytic activity of terrestrial isolate S1ACP6B on the CMC agar plate increased significantly at 30–37°C and reached its maximum activity at 37°C (CI ranges from 5.0 ± 1.8 to 5.9 ± 1.06) (Figure 3(b)). The marine isolate MS1OMP2A, on the other hand, showed an optimal activity at 25–30°C, with maximum activity at 30°C with a clearing zone of 4.4 ± 1.36. The optimum pH and temperature were then used for the following enzyme assay to determine whether an increase in enzyme activity would be observed. Results showed that terrestrial isolate S1ACP6B exhibited activity levels of 0.25 U/mg against CMC, 0.30 U/mg against Avicel, and 0.41 U/mg against filter paper (Figure 4(a)). On the other hand, marine isolate MS1OMP2A showed enzyme activity levels of 0.27 U/mg, 0.33 U/mg, and 0.29 U/mg against CMC, Avicel, and filter paper, respectively (Figure 4(b)). However, the statistical analysis, performed using ANOVA and Tukey's test, revealed a significant difference only in the optimization of CMCase enzyme for the terrestrial isolate S1ACP6B between the pre- and postoptimization stages (Table S5). The optimizations for the other enzymes did not yield significant changes. This suggests that the optimization of pH and temperature for enzyme assays appears to be beneficial specifically for CMCase derived from the terrestrial isolate but might not exert a similar influence on the other enzymes investigated in the study. It also implies that further optimization of factors beyond pH and temperature, such as enzyme inhibitors and cofactors, may be necessary to further enhance enzyme activities. In particular, marine enzymes might require optimization for additional factors, such as salinity, in the enzyme assays. Furthermore, in certain bacterial species, cellulases are anchored to cell walls and produced in complexes known as cellulosomes [47]. The presence of these cellulosomes and their complete dissociation into their individual components would also be necessary considerations during enzyme assays.

### 3.5. Molecular Identity of the Isolates

The eighteen isolates with high CIs were molecularly identified using 16S rRNA and ITS sequencing. The obtained sequences were then analyzed and compared to reference sequences in GenBank. Identity analysis using NCBI BLASTn revealed that the isolates were members of various families, including Bacillaceae (33.33%), Nocardiaceae (5.56%), Enterobacteriaceae (5.56%), Paenibacillaceae (16.67%), Pseudomonadaceae (5.56%), Rhodobacteraceae (5.56%), Cellulomonadaceae (5.56%), Saccharomycetaceae (5.56%), Halomonadaceae (5.56%), and Streptomycetaceae (11.11%) family (Table S6).

The two highest cellulase-producing isolates, S1ACP6B and MS1OMP2A, were identified as *Enterobacter roggenkampii* (99.88%) and *Rhodococcus erythropolis* (100%), respectively (Figures S4 and S5; Table S6). *E. roggenkampii* is a Gram-negative bacterium that belongs to the family of Enterobacteriaceae. It exhibits optimal growth at neutral pH and temperatures ranging from 30 to 37°C, which is consistent with the results of the current study [48]. In a study conducted by Guo et al. (2020), *E. roggenkampii* strain ED5, isolated from the sugarcane, showed cellulase and endoglucanase activities of 179.02 IU/mL and 1355.87 IU/mL, respectively. Moreover, strain ED5 displayed significant nitrogenase activity, making it a potential plant growth promoter [49]. In a recent study, *E. roggenkampii*, isolated from the gut of an insect pest *Neurozerra conferta* Walker, showed cellulase activity of 0.57 U/mL ± 0.001 [50]. These values are higher as compared to the cellulase (0.105 ± 0.003 U/mL) and endoglucanase (0.148 ± 0.002 U/mL) activities observed for *E. roggenkampii* in this study. Another study by Gan et al. reported the biodegradation of cellulose nanocrystal (CNC)/chitosan composite films by *E. roggenkampii*. These natural biopolymers are utilized as alternatives for plastics and have a higher crystallinity, which increases resistance to microbial degradation. The data also showed that *E. roggenkampii* and *E. kobei* have the highest optical density growth in liquid minimal salt media containing powdered chitosan composite films [51]. These studies show the superior capabilities of *E. roggenkampii* in the degradation of complex compounds.

On the other hand, *R. erythropolis* is an actinomycete, Gram-positive bacterium known for its hydrocarbon degrading abilities and bioremediation capabilities [52]. A previous study indicated that *Rhodococcus* species were incapable of hydrolyzing cellulose [53]. However, recent research contradicts this finding. According to a recent study, certain species in this genus possess the ability to degrade complex compounds, such as toluene, by 43 to 49% through its toluene monooxygenase gene [54]. Additionally, it has been discovered that *Rhodococcus sp.* can utilize lignin as a carbon source for growth and convert some lignin-derived compounds into triacylglycerols, making them a promising option for converting lignocellulosic biomass into biofuels [55]. Studies have proposed using genetic and metabolic engineering strategies to develop sustainable processes for biofuel production using *Rhodococcus* spp. strains, particularly *R. opacus* PD630 and *R. jostii* RHA1 [56]. However, there has been no comprehensive study specifically investigating the cellulolytic capacity of *R. erythropolis*.

Surprisingly, three bacterial isolates out of the eighteen isolates exhibited growth on CMC agar medium supplemented with chloramphenicol, indicating potential resistance to the antibiotic. These isolates were S2CZP6B, B1CZP7A, and S2CZP6A and were identified as *Streptomyces coacervatus* (99.52%), *Streptomyces chartreusis* (99.46%), and *Brevibacillus* sp. (99.92%) through 16S sequencing, respectively (Figures S6 and S7; Table S6). Interestingly, isolates S2CZP6B and B1CZP7A were the top two isolates with the highest CI on CMC agar medium (Table 1). These isolates were from Bulabog Putian National Park and sugarcane field located in the same municipality. Streptomyces genus is well-known producers of antibiotics and other secondary metabolites. *S. chartreusis* NRRL 3882 is a strain that produces calcimycin and tunicamycin [57]. Similarly, some *Brevibacillus* species, such as *B. agri*, are capable of producing antibiotics gramicidin and tyrocidine [58]. Cellulolytic microorganisms that exhibit moderately high antimicrobial properties were also reported by Daquioag and Peculiar, including seven *Streptomyces* species [59]. Additionally, *Streptomyces* bacteria found in soil serve as a significant source of genes associated with multiple antibiotic resistances. They possess resistance to antibiotics used in clinical setting, even in the absence of anthropogenic contamination [60, 61]. Antibiotic resistance in these isolates may be due to intrinsic characteristics of self-resistance [62]. Cellulolytic microorganisms that could produce antibiotics would be more beneficial for this could contribute to a more sustainable and efficient production of antibiotics from cellulose-based waste materials.

Three isolates, namely, B1CZP10A, MS1ACP1B, and MP2ACP3B, exhibited a query coverage or percent identity of less than 98% after a blast search in NCBI (Table S6). Any 16S rRNA or ITS gene sequence showing <98% sequence similarity can be considered as a distinct species, while a <95% may represent a different genus and require further characterization for proper identification [63–65]. MS1ACP1B and MP2ACP3B may potentially be new species related to *Halomonas zhaodongensis* (1149 bp/1192 bp) and *Albirhodobacter marinus* (1177 bp/1248 bp), with percent similarity of 96.39% and 94.31%, respectively. MS1ACP1B and MP2ACP3B were isolated from a mangrove area located at San Dionisio, Concepcion, Iloilo, and Pedada Mangrove Sanctuary in Ajuy, Iloilo, respectively. B1CZP10A, on the other hand, is believed to be a new species or genus of cellulolytic fungus related to *Saccharomyces* genus, which may have not been cultured before. Notably, B1C7P10A was isolated from an undisturbed environment in the Bulabog Putian National Park, which can indicate an increased likelihood of discovering new microorganisms.

**Figure 5:**
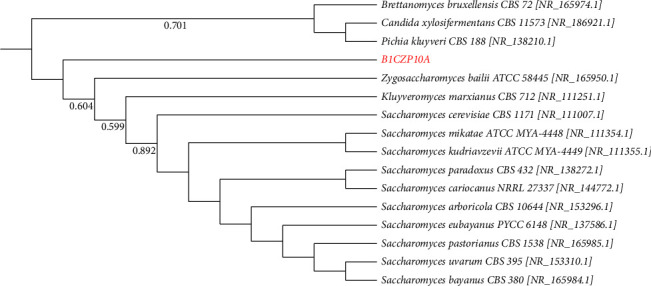
The maximum likelihood phylogenetic tree of the genus *Saccharomyces* based on the T92 + G model of DNA substitution; bootstrap values less than 50% are not shown. The tree is rooted on a member of *Brettanomyces, Candida,* and *Pichia*. Red font represents the isolate(s) in this study.

**Figure 6:**
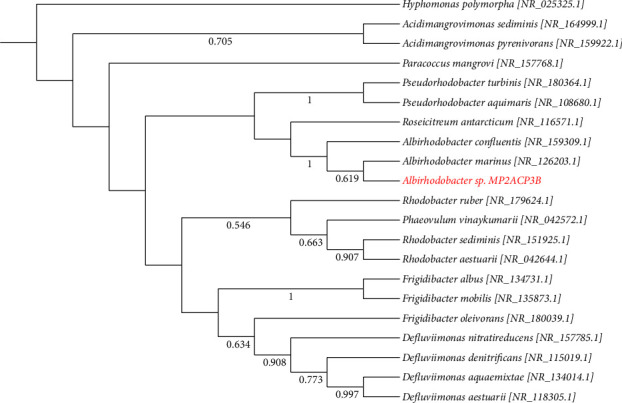
The neighbor-joining phylogenetic tree of the genus *Albirhodobacter* based on the K2 + G model of DNA substitution; bootstrap values less than 50% are not shown. The tree is rooted on a member of *Hyphomonas*. Red font represents the isolate(s) in this study.

**Figure 7:**
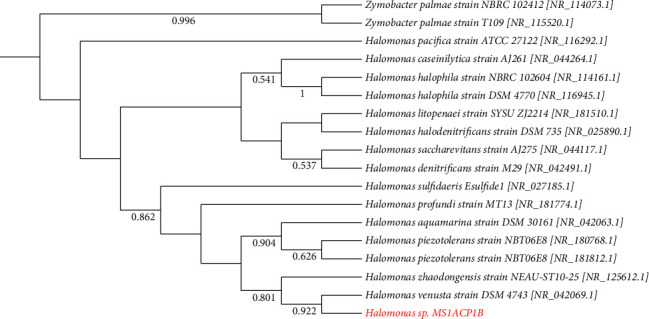
The maximum likelihood phylogenetic tree of the genus *Halomonas* based on the T92 + G model of DNA substitution; bootstrap values less than 50% are not shown. The tree is rooted on a member of *Zymobacter*. Red font represents the isolate(s) in this study.

In the phylogenetic trees constructed using the sequences of the isolates and downloaded sequences of various type strains, a number of isolates clustered with their closest match based on their BLAST results (Figures 5, 6, 7; Figures S4–S12). However, the BLAST identity of B1CZP10A did not support the phylogenetic analysis (Figure 5). The isolate may not belong to the genus *Saccharomyces* and may be from a different genus within the Saccharomycetaceae or another family of fungi. The same uncertainty applied to other isolates like MP1ACP1B, as their identities did not clearly reflect or align with their phylogenetic positions (Figure S11). This suggests the need for additional polyphasic taxonomic studies to determine their specific names. Overall, this study provides valuable insights into the composition and activities of cellulolytic microorganisms in various habitats in Panay Island, Philippines.

### 3.6. Biochemical Characterization of the Possible Novel Isolates

Biochemical characterization was conducted on the three possible novel isolates. Cultural examination revealed the colony characteristics of the following isolates on NA or PDA medium: (1) MS1ACP1B, identified as related to *Halomonas* sp., appeared off-white, irregular, has convex elevation and curled margin; (2) MP2ACP3B, identified as related to *Albirhodobacter marinus*, appeared off-white to yellow, circular, has a convex elevation and an entire margin; and, lastly, (3) isolate B1CZP10A, identified as related to *Saccharomyces* genus, appeared to have dry chalky white to dirty white colonies and ciliated margin. Through Gram staining and microscopy, it was observed that the marine isolates, MS1ACP1B and MP2ACP3B, were rod-shaped, Gram-negative bacteria. On the other hand, microscopic observations in B1CZP10A revealed the presence of hyphae-like structures.

The biochemical properties of the three possible novel species are shown in Table 2. Isolate MS1ACP1B demonstrates biochemical properties more akin to *H. venusta* rather than its corresponding identity, *H. zhaodongensis*. The phylogenetic analysis further indicates a close relationship between isolate MS1ACP1B and *H. venusta* (Figure 7). Both MS1ACP1B and *H. venusta* exhibit positive catalase activity and oxidize D-maltose, sucrose, glucose, and D-glycerol [30]. However, MS1ACP1B differs by fermenting lactose while being unable to ferment mannitol, in contrast to *H. venusta*, which lacks lactose fermentation ability can utilize mannitol.

As for isolate M2ACP3B, its phylogenetic analysis suggests a close association with *A. marinus* within the Rhodobacteraceae family (Figure 6). *A. marinus* is characterized by its nonmotility, negative indole production, and oxidation of substrates, such as urea, lactose, glucose, and mannitol but not casein, gelatin, starch, sucrose, or maltose [31]. It shares similar physiological characteristics with isolate M2ACP3B, except that M2ACP3B can hydrolyze starch, sucrose, and maltose but not mannitol, urea, casein, or gelatin. In contrast, another species, *A. confluentis*, is motile and utilizes casein, gelatin, mannitol, and maltose [32].

Isolate B1CZP10A, identified as closely related to the *Saccharomyces* genus, exhibits positive catalase activity but does not utilize sugars except for starch and glucose, unlike *S. cerevisiae* var. *boulardii*, which shows utilization of certain sugars such as sucrose [33]. Extensive characterization and whole-genome sequencing are needed to further verify the novelty of these three cellulolytic microorganisms.

## 4. Conclusion

Diverse microorganisms thrive in the soil. Microbial activities in the soil support the growth of higher organisms through the recycling of nutrients; hence, microbial activities promote fertility and productivity. Cellulolytic microorganisms are especially important as they act as the first line of organic matter decomposition and could be the key to solving the climate issue and enabling the transition to a bioenergy future. This study focused on identifying and characterizing cellulolytic microorganisms in soil samples obtained from seven different locations in Panay Island. Results showed that terrestrial habitats had significantly higher levels of cellulolytic microorganisms than mangrove habitats, potentially due to environmental factors such as nutrient availability and plant diversity. The study also revealed a diverse community of cellulolytic bacteria dominated by *Niallia, Rhodococcus, Bacillus, Enterobacter, Brevibacillus, Stutzerimonas, Siminovitchia, Cellulomonas, Streptomyces,* and the existence of possible novel species, which needs further characterization.

Meanwhile, the enzymatic activities of the isolated colonies were evaluated to determine their economic viability. Results showed that S1ACP6B from the sugarcane field and MS1OMP2A from the marine environment had the highest cellulase activities among all isolates, which were identified as *Enterobacter roggenkampii* and *Rhodococcus erythropolis*, respectively. However, despite the high diversity of cellulolytic microorganisms, very few have commercial use potential due to their low cellulase activities. Enhancing the CMCase enzymatic activity of certain isolates may be achieved through the optimization of microbial growth conditions, including pH and temperature. However, further investigation and optimization of the cellulolytic activities of S1ACP6B and MS1OMP2A are essential prerequisites before considering their commercial application. Overall, this research contributes to the understanding of the diversity of cellulolytic microorganisms in the soils of Panay Island and may serve as a vital resource in the search for novel cellulolytic microorganisms with potential technological applications.

## Figures and Tables

**Table 1:** Enzyme activities of the top eighteen terrestrial and marine isolates on CMC, Avicel, and Filter paper after three days of incubation in cellulose broth at 30°C.

Code	CMCase (U/mg)	Avicelase (U/mg)	FPase (U/mg)	CI
*Terrestrial*				
S2CZP6B	0.099 ± 0.01^g^	0.136 ± 0.06^b,c,d^	0.143 ± 0.00^g,h^	6.70 ± 0.00^a^^*∗*^
B1CZP7A	0.141 ± 0.02^f,g^	0.132 ± 0.04^c,d^	0.127 ± 0.03^h^	5.70 ± 0.67^b^
R1OMP1C	0.138 ± 0.00^f,g^	0.173 ± 0.00^b,c,d^	0.167 ± 0.00^d,e,f,g,h^	5.10 ± 0.13^b,c^
S1ACP4C	0.206 ± 0.02^b,c,d,e^	0.204 ± 0.02^b,c,d^	0.260 ± 0.01^b,c^	4.80 ± 0.20^c,d^
S1ACP5A	0.147 ± 0.00^f,g^	0.127 ± 0.00^c,d^	0.156 ± 0.03^e,f,g,h^	4.50 ± 0.14^c,d^
S1ACP6B	0.155 ± 0.00^e,f,g^	0.192 ± 0.09^b,c,d^	0.390 ± 0.01^a^^*∗*^	4.40 ± 0.08^c,d^
B2OMP3A	0.246 ± 0.02^a,b^^*∗*^	0.195 ± 0.01^b,c,d^	0.201 ± 0.01^c,d,e,f,g^	4.30 ± 0.37^d^
R1ACD1A	0.120 ± 0.00^g^	0.103 ± 0.03^d^	0.221 ± 0.04^c,d,e^	4.30 ± 0.43^d^
S1ACP2C	0.230 ± 0.01^a,b,c^	0.242 ± 0.08^a,b,c,d^^*∗*^	0.148 ± 0.02^g,h^	4.30 ± 0.42^d^
S2ACP4B	0.138 ± 0.00^f,g^	0.103 ± 0.02^d^	0.293 ± 0.01^b^	4.20 ± 0.19^d,e^
B1CZP10A	0.214 ± 0.02^b,c,d^	0.169 ± 0.00^b,c,d^	0.216 ± 0.02^c,d,e,f^	4.10 ± 0.18^d,e^
S2CZP6A	0.179 ± 0.03^c,d,e,f^	0.209 ± 0.03^b,c,d^	0.165 ± 0.01^d,e,f,g,h^	4.10 ± 0.39^d,e,f^

*Marine*				
ML1OMP2A	0.156 ± 0.02^d,e,f,g^	0.131 ± 0.00^c,d^	0.225 ± 0.02^c,d^	4.50 ± 0.00^c,d^^*∗*^
MS1OMP2A	0.279 ± 0.01^a^	0.271 ± 0.09^a,b,c^	0.265 ± 0.04^b,c^^*∗*^	4.40 ± 0.08^c,d^
MP1ACP1B	0.254 ± 0.02^a,b^	0.275 ± 0.03^a,b,c^	0.146 ± 0.03^g,h^	4.20 ± 0.19^d,e^
ML2OMP1B	0.262 ± 0.03^a,b^	0.289 ± 0.06^a,b^	0.237 ± 0.01^b,c^	3.30 ± 0.07^e,f,g^
MS1ACP1B	0.281 ± 0.05^a^^*∗*^	0.370 ± 0.11^a^^*∗*^	0.164 ± 0.04^d,e,f,g,h^	3.20 ± 0.28^f,g^
MP2ACP3B	0.151 ± 0.01^e,f,g^	0.211 ± 0.04^b,c,d^	0.154 ± 0.02^f,g,h^	3.00 ± 0.23^g^

^a,b,c,d,e,f,g,h^Grouping information using the Tukey method and 95% confidence; means that do not share a letter are significantly different; ^*∗*^Highest values.

**Table 2:** Biochemical properties of the three possible novel cellulolytic microorganisms.

Tests	Isolates			Control		Reference species (type strains)				
	MS1ACP1B (*Halomonas* sp.)	MP2ACP3B (*Albirhodobacter* sp.)	B1CZP10A (yeast)	*E. coli*	*B. subtilis*	*Halomonas venusta* DSM 4743^*∗*^	*Halomonas halophila* F 5-7^*∗*^	*Albirhodobacter marinus* N9^a^	*Albirhodobacter confluentis^b^*	Saccharomyces cerevisiae var. boulardii^c^
Gram staining	−	−	NA	−	+	−	−	−	−	NA
Indole	−	−	−	+	−	−	−	−	−	ND
Motility	+	−	−	+	−	+	+	−	+	−
Catalase	+	+	+	+	+	+	+	+	+	ND
Casein	−	−	−	−	+	−	ND	−	+	ND
Gelatin	−	−	−	−	+	−	−	−	+	−
Starch	+	+	+	−	+	ND	−	−	−	+
Urea	−	−	−	−	−	−	−	+	−	−
Glycerol	+	+	−	+	+	+	−	ND	ND	ND
Lactose	+	+	−	+	−	−	−	+	ND	−
Glucose	+	+	+	+	+	+	−	+	−	+
Sucrose	+	+	−	+	+	+	−	−	ND	+
Mannitol	−	−	−	+	−	+	−	+	+	ND
Maltose	+	+	−	+	+	+	−	−	+	ND
MR	−	−	−	+	NA	ND	ND	ND	−	ND
VP	−	−	−	−	NA	ND	ND	ND	ND	ND
Citrate	+	−	−	−	NA	+	−	−	+	ND

(+) indicates positive test; (−) indicates negative test; NA, not applicable; ND, no data available in literature. ^*∗*^Type species data retrieved from The Bacterial Diversity Metadatabase (BacDive) [30], ^a^[31], ^b^[32], ^c^[33].

## Data Availability

All raw data used to support the findings of this study are available from the corresponding author upon request.

## Supplementary Materials

Supplementary Materials Supplementary 1. Fig. S1: Example of cellulolytic microorganisms isolated from the collected soils within Panay Island. A: actinomycete; B: bacteria; C–D: fungi; E–F: pigmented isolates. Supplementary 2. Fig. S2: Example of cellulolytic microorganisms growing on agar plates with rice husk (A) or cellulose (B) as the sole carbon source. Supplementary 3. Fig. S3: Example of cellulolytic microorganisms showing clear zones on CMC agar plates following the addition of 1% Gram iodine solution. Supplementary 4. Fig. S4: The maximum likelihood phylogenetic tree of the genus Enterobacter based on the K2 + G + I model of DNA substitution; bootstrap values less than 50% are not shown. The tree is rooted on Escherichia coli. Red font represents the isolate(s) in this study. Supplementary 5. Fig. S5: The neighbor-joining phylogenetic tree of the genus Rhodococcus based on the TN93 + G model of DNA substitution; bootstrap values less than 50% are not shown. The tree is rooted on a member of Corynebacterium. Red font represents the isolate(s) in this study. Supplementary 6. Fig. S6: The maximum likelihood phylogenetic tree of the genus Streptomyces based on the T92 + G model of DNA substitution; bootstrap values less than 50% are not shown. The tree is rooted on a member of Kitasatospora. Red font represents the isolate(s) in this study. Supplementary 7. Fig. S7: The neighbor-joining phylogenetic tree of the genus Brevibacillus based on the T92 + G model of DNA substitution; bootstrap values less than 50% are not shown. The tree is rooted on members of Bacillus and Paenibacillus. Red font represents the isolate(s) in this study. Supplementary 8. Fig. S8: The maximum likelihood phylogenetic tree of the genus Cellulomonas based on the K2 + G model of DNA substitution; bootstrap values less than 50% are not shown. The tree is rooted on a member of Actinotalea. Red font represents the isolate(s) in this study. Supplementary 9. Fig. S9: The neighbor-joining phylogenetic tree of the genus Niallia based on the T92 + G model of DNA substitution; bootstrap values less than 50% are not shown. The tree is rooted on a member of Paenibacillus and Bacillus. Red font represents the isolate(s) in this study. Supplementary 10. Fig. S10: The maximum likelihood phylogenetic tree of the genus Siminovitchia based on the T92 + G + I model of DNA substitution; bootstrap values less than 50% are not shown. The tree is rooted on a member of Bacillus. Red font represents the isolate(s) in this study. Supplementary 11. Fig. S11: The neighbor-joining phylogenetic tree of the genus Stutzerimonas (presumptive identity of MP1ACP1B isolate) based on the K2 + G model of DNA substitution; bootstrap values less than 50% are not shown. The tree is rooted on a member of Rhizobacter, Brevundimonas, Marinobacter, and E. coli. Red font represents the isolate(s) in this study. Supplementary 12. Fig. S12: The maximum likelihood phylogenetic tree of the genus Bacillus based on the T92 + G model of DNA substitution; bootstrap values less than 50% are not shown. The tree is rooted on members of Acetobacter, Paenibacillus, and Geobacillus. Red font represents the isolate(s) in this study. Supplementary 13. Table S1: Summary of the soil sample analysis data and sampling sites. Supplementary 14. Table S2: Statistical analysis of enzyme assays and CIs among isolates using ANOVA. Supplementary 15. Table S3: Statistical analysis of enzyme assay between the terrestrial and marine environments using ANOVA and Tukey's test. Supplementary 16. Table S4: Estimated marginal means of the enzyme assays and cellulolytic index between the two environments. Supplementary 17. Table S5: Statistical analysis of pre- and postoptimization of enzyme assay between S1ACP6B and MS1OMP2A isolates using ANOVA and Tukey's test. Supplementary 18. Table S6: The molecular identity of the eighteen cellulolytic isolates using 16S rRNA and ITS gene sequences.
