# Supplementary material for: Diversity and Screening of Cellulolytic Microorganisms from Mangrove Forests, Natural Parks, Paddy Field, and Sugarcane Plantation in Panay Island, Philippines
Source: Int J Microbiol. 2024 Jul 23;2024:5573158. doi: 10.1155/2024/5573158 (PMC11288694; doi:10.1155/2024/5573158)
Supplement: Supplementary Materials — Supplementary 1. Fig. S1: Example of cellulolytic microorganisms isolated from the collected soils within Panay Island. A: actinomycete; B: bacteria; C–D: fungi; E–F: pigmented isolates. Supplementary 2. Fig. S2: Example of cellulolytic microorganisms growing on agar plates with rice husk (A) or cellulose (B) as the sole carbon source. Supplementary 3. Fig. S3: Example of cellulolytic microorganisms showing clear zones on CMC agar plates following the addition of 1% Gram iodine solution. Supplementary 4. Fig. S4: The maximum likelihood phylogenetic tree of the genus Enterobacter based on the K2 + G + I model of DNA substitution; bootstrap values less than 50% are not shown. The tree is rooted on Escherichia coli. Red font represents the isolate(s) in this study. Supplementary 5. Fig. S5: The neighbor-joining phylogenetic tree of the genus Rhodococcus based on the TN93 + G model of DNA substitution; bootstrap values less than 50% are not shown. The tree is rooted on a member of Corynebacterium. Red font represents the isolate(s) in this study. Supplementary 6. Fig. S6: The maximum likelihood phylogenetic tree of the genus Streptomyces based on the T92 + G model of DNA substitution; bootstrap values less than 50% are not shown. The tree is rooted on a member of Kitasatospora. Red font represents the isolate(s) in this study. Supplementary 7. Fig. S7: The neighbor-joining phylogenetic tree of the genus Brevibacillus based on the T92 + G model of DNA substitution; bootstrap values less than 50% are not shown. The tree is rooted on members of Bacillus and Paenibacillus. Red font represents the isolate(s) in this study. Supplementary 8. Fig. S8: The maximum likelihood phylogenetic tree of the genus Cellulomonas based on the K2 + G model of DNA substitution; bootstrap values less than 50% are not shown. The tree is rooted on a member of Actinotalea. Red font represents the isolate(s) in this study. Supplementary 9. Fig. S9: The neighbor-joining phylogenetic tree of the gen [file 5573158.f1.zip › Post Hoc tests.docx]

**Post Hoc tests**

| **Isolates** | **Code** | **Isolates** | **Code** |
| --- | --- | --- | --- |
| MS1ACP1B | AC2.2 | ML1OMP2A | OM2.1 |
| MS1OMP2A | OM2.2 | S1ACP6B | AC1.3 |
| ML2OMP1B | OM2.3 | MP2ACP3B | AC2.3 |
| MP1ACP1B | AC2.1 | S1ACP5A | AC1.2 |
| B2OMP3A | OM1.2 | B1CZP7A | CZ1.2 |
| S1ACP2C | AC1.6 | R1OMP1C | OM1.1 |
| B1CZP10A | CZ1.3 | S2ACP4B | AC1.5 |
| S1ACP4C | AC1.1 | R1ACD1A | AC1.4 |
| S2CZP6A | CZ1.4 | S2CZP6B | CZ1.1 |

| **CMCase** Post Hoc Comparisons - Isolate Code | | | | | | | | | | | | | | | |
| --- | --- | --- | --- | --- | --- | --- | --- | --- | --- | --- | --- | --- | --- | --- | --- |
| **Comparison** | | | | | |  | | | | | | | | | |
| **Isolate Code** | |  | | **Isolate Code** | | **Mean Difference** | | **SE** | | **df** | | **t** | | **p_tukey_** | |
| AC1.4 |  | - |  | AC1.6 |  | -0.10985 |  | 0.0156 |  | 36.0 |  | -7.0562 |  | < .001 |  |
|  |  | - |  | CZ1.1 |  | 0.02082 |  | 0.0156 |  | 36.0 |  | 1.3372 |  | 0.995 |  |
|  |  | - |  | CZ1.2 |  | -0.02077 |  | 0.0156 |  | 36.0 |  | -1.3345 |  | 0.995 |  |
|  |  | - |  | AC1.1 |  | -0.08648 |  | 0.0156 |  | 36.0 |  | -5.5551 |  | < .001 |  |
|  |  | - |  | AC1.2 |  | -0.02722 |  | 0.0156 |  | 36.0 |  | -1.7488 |  | 0.943 |  |
|  |  | - |  | AC1.3 |  | -0.03536 |  | 0.0156 |  | 36.0 |  | -2.2710 |  | 0.697 |  |
|  |  | - |  | AC1.5 |  | -0.01787 |  | 0.0156 |  | 36.0 |  | -1.1477 |  | 0.999 |  |
|  |  | - |  | OM2.1 |  | -0.03575 |  | 0.0156 |  | 36.0 |  | -2.2967 |  | 0.680 |  |
|  |  | - |  | OM2.2 |  | -0.15907 |  | 0.0156 |  | 36.0 |  | -10.2177 |  | < .001 |  |
|  |  | - |  | OM2.3 |  | -0.14201 |  | 0.0156 |  | 36.0 |  | -9.1221 |  | < .001 |  |
|  |  | - |  | AC2.1 |  | -0.13416 |  | 0.0156 |  | 36.0 |  | -8.6180 |  | < .001 |  |
|  |  | - |  | AC2.2 |  | -0.16121 |  | 0.0156 |  | 36.0 |  | -10.3553 |  | < .001 |  |
|  |  | - |  | AC2.3 |  | -0.03053 |  | 0.0156 |  | 36.0 |  | -1.9611 |  | 0.868 |  |
|  |  | - |  | CZ1.3 |  | -0.09372 |  | 0.0156 |  | 36.0 |  | -6.0203 |  | < .001 |  |
|  |  | - |  | CZ1.4 |  | -0.05882 |  | 0.0156 |  | 36.0 |  | -3.7782 |  | 0.047 |  |
|  |  | - |  | OM1.2 |  | -0.12652 |  | 0.0156 |  | 36.0 |  | -8.1272 |  | < .001 |  |
|  |  | - |  | OM1.1 |  | -0.01808 |  | 0.0156 |  | 36.0 |  | -1.1617 |  | 0.999 |  |
| AC1.6 |  | - |  | CZ1.1 |  | 0.13067 |  | 0.0156 |  | 36.0 |  | 8.3933 |  | < .001 |  |
|  |  | - |  | CZ1.2 |  | 0.08907 |  | 0.0156 |  | 36.0 |  | 5.7217 |  | < .001 |  |
|  |  | - |  | AC1.1 |  | 0.02337 |  | 0.0156 |  | 36.0 |  | 1.5010 |  | 0.985 |  |
|  |  | - |  | AC1.2 |  | 0.08262 |  | 0.0156 |  | 36.0 |  | 5.3074 |  | < .001 |  |
|  |  | - |  | AC1.3 |  | 0.07449 |  | 0.0156 |  | 36.0 |  | 4.7851 |  | 0.003 |  |
|  |  | - |  | AC1.5 |  | 0.09198 |  | 0.0156 |  | 36.0 |  | 5.9085 |  | < .001 |  |
|  |  | - |  | OM2.1 |  | 0.07409 |  | 0.0156 |  | 36.0 |  | 4.7594 |  | 0.003 |  |
|  |  | - |  | OM2.2 |  | -0.04922 |  | 0.0156 |  | 36.0 |  | -3.1616 |  | 0.185 |  |
|  |  | - |  | OM2.3 |  | -0.03216 |  | 0.0156 |  | 36.0 |  | -2.0659 |  | 0.817 |  |
|  |  | - |  | AC2.1 |  | -0.02431 |  | 0.0156 |  | 36.0 |  | -1.5619 |  | 0.978 |  |
|  |  | - |  | AC2.2 |  | -0.05136 |  | 0.0156 |  | 36.0 |  | -3.2991 |  | 0.140 |  |
|  |  | - |  | AC2.3 |  | 0.07932 |  | 0.0156 |  | 36.0 |  | 5.0950 |  | 0.001 |  |
|  |  | - |  | CZ1.3 |  | 0.01613 |  | 0.0156 |  | 36.0 |  | 1.0359 |  | 1.000 |  |
|  |  | - |  | CZ1.4 |  | 0.05103 |  | 0.0156 |  | 36.0 |  | 3.2780 |  | 0.146 |  |
|  |  | - |  | OM1.2 |  | -0.01667 |  | 0.0156 |  | 36.0 |  | -1.0710 |  | 1.000 |  |
|  |  | - |  | OM1.1 |  | 0.09176 |  | 0.0156 |  | 36.0 |  | 5.8945 |  | < .001 |  |
| CZ1.1 |  | - |  | CZ1.2 |  | -0.04159 |  | 0.0156 |  | 36.0 |  | -2.6716 |  | 0.433 |  |
|  |  | - |  | AC1.1 |  | -0.10730 |  | 0.0156 |  | 36.0 |  | -6.8923 |  | < .001 |  |
|  |  | - |  | AC1.2 |  | -0.04804 |  | 0.0156 |  | 36.0 |  | -3.0859 |  | 0.215 |  |
|  |  | - |  | AC1.3 |  | -0.05617 |  | 0.0156 |  | 36.0 |  | -3.6082 |  | 0.070 |  |
|  |  | - |  | AC1.5 |  | -0.03868 |  | 0.0156 |  | 36.0 |  | -2.4849 |  | 0.555 |  |
|  |  | - |  | OM2.1 |  | -0.05657 |  | 0.0156 |  | 36.0 |  | -3.6339 |  | 0.066 |  |
|  |  | - |  | OM2.2 |  | -0.17988 |  | 0.0156 |  | 36.0 |  | -11.5549 |  | < .001 |  |
|  |  | - |  | OM2.3 |  | -0.16283 |  | 0.0156 |  | 36.0 |  | -10.4593 |  | < .001 |  |
|  |  | - |  | AC2.1 |  | -0.15498 |  | 0.0156 |  | 36.0 |  | -9.9552 |  | < .001 |  |
|  |  | - |  | AC2.2 |  | -0.18203 |  | 0.0156 |  | 36.0 |  | -11.6925 |  | < .001 |  |
|  |  | - |  | AC2.3 |  | -0.05135 |  | 0.0156 |  | 36.0 |  | -3.2983 |  | 0.140 |  |
|  |  | - |  | CZ1.3 |  | -0.11454 |  | 0.0156 |  | 36.0 |  | -7.3574 |  | < .001 |  |
|  |  | - |  | CZ1.4 |  | -0.07963 |  | 0.0156 |  | 36.0 |  | -5.1154 |  | 0.001 |  |
|  |  | - |  | OM1.2 |  | -0.14734 |  | 0.0156 |  | 36.0 |  | -9.4644 |  | < .001 |  |
|  |  | - |  | OM1.1 |  | -0.03890 |  | 0.0156 |  | 36.0 |  | -2.4988 |  | 0.545 |  |
| CZ1.2 |  | - |  | AC1.1 |  | -0.06571 |  | 0.0156 |  | 36.0 |  | -4.2207 |  | 0.015 |  |
|  |  | - |  | AC1.2 |  | -0.00645 |  | 0.0156 |  | 36.0 |  | -0.4143 |  | 1.000 |  |
|  |  | - |  | AC1.3 |  | -0.01458 |  | 0.0156 |  | 36.0 |  | -0.9366 |  | 1.000 |  |
|  |  | - |  | AC1.5 |  | 0.00291 |  | 0.0156 |  | 36.0 |  | 0.1868 |  | 1.000 |  |
|  |  | - |  | OM2.1 |  | -0.01498 |  | 0.0156 |  | 36.0 |  | -0.9623 |  | 1.000 |  |
|  |  | - |  | OM2.2 |  | -0.13829 |  | 0.0156 |  | 36.0 |  | -8.8833 |  | < .001 |  |
|  |  | - |  | OM2.3 |  | -0.12124 |  | 0.0156 |  | 36.0 |  | -7.7876 |  | < .001 |  |
|  |  | - |  | AC2.1 |  | -0.11339 |  | 0.0156 |  | 36.0 |  | -7.2836 |  | < .001 |  |
|  |  | - |  | AC2.2 |  | -0.14043 |  | 0.0156 |  | 36.0 |  | -9.0208 |  | < .001 |  |
|  |  | - |  | AC2.3 |  | -0.00976 |  | 0.0156 |  | 36.0 |  | -0.6267 |  | 1.000 |  |
|  |  | - |  | CZ1.3 |  | -0.07295 |  | 0.0156 |  | 36.0 |  | -4.6858 |  | 0.004 |  |
|  |  | - |  | CZ1.4 |  | -0.03804 |  | 0.0156 |  | 36.0 |  | -2.4437 |  | 0.583 |  |
|  |  | - |  | OM1.2 |  | -0.10575 |  | 0.0156 |  | 36.0 |  | -6.7927 |  | < .001 |  |
|  |  | - |  | OM1.1 |  | 0.00269 |  | 0.0156 |  | 36.0 |  | 0.1728 |  | 1.000 |  |
| AC1.1 |  | - |  | AC1.2 |  | 0.05926 |  | 0.0156 |  | 36.0 |  | 3.8064 |  | 0.044 |  |
|  |  | - |  | AC1.3 |  | 0.05113 |  | 0.0156 |  | 36.0 |  | 3.2841 |  | 0.144 |  |
|  |  | - |  | AC1.5 |  | 0.06861 |  | 0.0156 |  | 36.0 |  | 4.4074 |  | 0.009 |  |
|  |  | - |  | OM2.1 |  | 0.05073 |  | 0.0156 |  | 36.0 |  | 3.2584 |  | 0.152 |  |
|  |  | - |  | OM2.2 |  | -0.07259 |  | 0.0156 |  | 36.0 |  | -4.6626 |  | 0.004 |  |
|  |  | - |  | OM2.3 |  | -0.05553 |  | 0.0156 |  | 36.0 |  | -3.5670 |  | 0.077 |  |
|  |  | - |  | AC2.1 |  | -0.04768 |  | 0.0156 |  | 36.0 |  | -3.0629 |  | 0.224 |  |
|  |  | - |  | AC2.2 |  | -0.07473 |  | 0.0156 |  | 36.0 |  | -4.8002 |  | 0.003 |  |
|  |  | - |  | AC2.3 |  | 0.05595 |  | 0.0156 |  | 36.0 |  | 3.5940 |  | 0.073 |  |
|  |  | - |  | CZ1.3 |  | -0.00724 |  | 0.0156 |  | 36.0 |  | -0.4651 |  | 1.000 |  |
|  |  | - |  | CZ1.4 |  | 0.02766 |  | 0.0156 |  | 36.0 |  | 1.7769 |  | 0.935 |  |
|  |  | - |  | OM1.2 |  | -0.04004 |  | 0.0156 |  | 36.0 |  | -2.5721 |  | 0.497 |  |
|  |  | - |  | OM1.1 |  | 0.06840 |  | 0.0156 |  | 36.0 |  | 4.3935 |  | 0.009 |  |
| AC1.2 |  | - |  | AC1.3 |  | -0.00813 |  | 0.0156 |  | 36.0 |  | -0.5223 |  | 1.000 |  |
|  |  | - |  | AC1.5 |  | 0.00936 |  | 0.0156 |  | 36.0 |  | 0.6011 |  | 1.000 |  |
|  |  | - |  | OM2.1 |  | -0.00853 |  | 0.0156 |  | 36.0 |  | -0.5480 |  | 1.000 |  |
|  |  | - |  | OM2.2 |  | -0.13184 |  | 0.0156 |  | 36.0 |  | -8.4690 |  | < .001 |  |
|  |  | - |  | OM2.3 |  | -0.11479 |  | 0.0156 |  | 36.0 |  | -7.3734 |  | < .001 |  |
|  |  | - |  | AC2.1 |  | -0.10694 |  | 0.0156 |  | 36.0 |  | -6.8693 |  | < .001 |  |
|  |  | - |  | AC2.2 |  | -0.13398 |  | 0.0156 |  | 36.0 |  | -8.6065 |  | < .001 |  |
|  |  | - |  | AC2.3 |  | -0.00331 |  | 0.0156 |  | 36.0 |  | -0.2124 |  | 1.000 |  |
|  |  | - |  | CZ1.3 |  | -0.06650 |  | 0.0156 |  | 36.0 |  | -4.2715 |  | 0.013 |  |
|  |  | - |  | CZ1.4 |  | -0.03159 |  | 0.0156 |  | 36.0 |  | -2.0294 |  | 0.836 |  |
|  |  | - |  | OM1.2 |  | -0.09930 |  | 0.0156 |  | 36.0 |  | -6.3784 |  | < .001 |  |
|  |  | - |  | OM1.1 |  | 0.00914 |  | 0.0156 |  | 36.0 |  | 0.5871 |  | 1.000 |  |
| AC1.3 |  | - |  | AC1.5 |  | 0.01749 |  | 0.0156 |  | 36.0 |  | 1.1233 |  | 0.999 |  |
|  |  | - |  | OM2.1 |  | -4.00e−4 |  | 0.0156 |  | 36.0 |  | -0.0257 |  | 1.000 |  |
|  |  | - |  | OM2.2 |  | -0.12371 |  | 0.0156 |  | 36.0 |  | -7.9467 |  | < .001 |  |
|  |  | - |  | OM2.3 |  | -0.10666 |  | 0.0156 |  | 36.0 |  | -6.8511 |  | < .001 |  |
|  |  | - |  | AC2.1 |  | -0.09881 |  | 0.0156 |  | 36.0 |  | -6.3470 |  | < .001 |  |
|  |  | - |  | AC2.2 |  | -0.12585 |  | 0.0156 |  | 36.0 |  | -8.0842 |  | < .001 |  |
|  |  | - |  | AC2.3 |  | 0.00482 |  | 0.0156 |  | 36.0 |  | 0.3099 |  | 1.000 |  |
|  |  | - |  | CZ1.3 |  | -0.05837 |  | 0.0156 |  | 36.0 |  | -3.7492 |  | 0.050 |  |
|  |  | - |  | CZ1.4 |  | -0.02346 |  | 0.0156 |  | 36.0 |  | -1.5071 |  | 0.984 |  |
|  |  | - |  | OM1.2 |  | -0.09117 |  | 0.0156 |  | 36.0 |  | -5.8561 |  | < .001 |  |
|  |  | - |  | OM1.1 |  | 0.01727 |  | 0.0156 |  | 36.0 |  | 1.1094 |  | 0.999 |  |
| AC1.5 |  | - |  | OM2.1 |  | -0.01789 |  | 0.0156 |  | 36.0 |  | -1.1490 |  | 0.999 |  |
|  |  | - |  | OM2.2 |  | -0.14120 |  | 0.0156 |  | 36.0 |  | -9.0700 |  | < .001 |  |
|  |  | - |  | OM2.3 |  | -0.12414 |  | 0.0156 |  | 36.0 |  | -7.9744 |  | < .001 |  |
|  |  | - |  | AC2.1 |  | -0.11630 |  | 0.0156 |  | 36.0 |  | -7.4703 |  | < .001 |  |
|  |  | - |  | AC2.2 |  | -0.14334 |  | 0.0156 |  | 36.0 |  | -9.2076 |  | < .001 |  |
|  |  | - |  | AC2.3 |  | -0.01266 |  | 0.0156 |  | 36.0 |  | -0.8134 |  | 1.000 |  |
|  |  | - |  | CZ1.3 |  | -0.07585 |  | 0.0156 |  | 36.0 |  | -4.8726 |  | 0.002 |  |
|  |  | - |  | CZ1.4 |  | -0.04095 |  | 0.0156 |  | 36.0 |  | -2.6305 |  | 0.459 |  |
|  |  | - |  | OM1.2 |  | -0.10865 |  | 0.0156 |  | 36.0 |  | -6.9795 |  | < .001 |  |
|  |  | - |  | OM1.1 |  | -2.17e−4 |  | 0.0156 |  | 36.0 |  | -0.0140 |  | 1.000 |  |
| OM2.1 |  | - |  | OM2.2 |  | -0.12331 |  | 0.0156 |  | 36.0 |  | -7.9210 |  | < .001 |  |
|  |  | - |  | OM2.3 |  | -0.10626 |  | 0.0156 |  | 36.0 |  | -6.8254 |  | < .001 |  |
|  |  | - |  | AC2.1 |  | -0.09841 |  | 0.0156 |  | 36.0 |  | -6.3213 |  | < .001 |  |
|  |  | - |  | AC2.2 |  | -0.12545 |  | 0.0156 |  | 36.0 |  | -8.0585 |  | < .001 |  |
|  |  | - |  | AC2.3 |  | 0.00522 |  | 0.0156 |  | 36.0 |  | 0.3356 |  | 1.000 |  |
|  |  | - |  | CZ1.3 |  | -0.05797 |  | 0.0156 |  | 36.0 |  | -3.7235 |  | 0.053 |  |
|  |  | - |  | CZ1.4 |  | -0.02306 |  | 0.0156 |  | 36.0 |  | -1.4815 |  | 0.987 |  |
|  |  | - |  | OM1.2 |  | -0.09077 |  | 0.0156 |  | 36.0 |  | -5.8304 |  | < .001 |  |
|  |  | - |  | OM1.1 |  | 0.01767 |  | 0.0156 |  | 36.0 |  | 1.1351 |  | 0.999 |  |
| OM2.2 |  | - |  | OM2.3 |  | 0.01706 |  | 0.0156 |  | 36.0 |  | 1.0956 |  | 1.000 |  |
|  |  | - |  | AC2.1 |  | 0.02490 |  | 0.0156 |  | 36.0 |  | 1.5997 |  | 0.973 |  |
|  |  | - |  | AC2.2 |  | -0.00214 |  | 0.0156 |  | 36.0 |  | -0.1375 |  | 1.000 |  |
|  |  | - |  | AC2.3 |  | 0.12854 |  | 0.0156 |  | 36.0 |  | 8.2566 |  | < .001 |  |
|  |  | - |  | CZ1.3 |  | 0.06535 |  | 0.0156 |  | 36.0 |  | 4.1975 |  | 0.016 |  |
|  |  | - |  | CZ1.4 |  | 0.10025 |  | 0.0156 |  | 36.0 |  | 6.4395 |  | < .001 |  |
|  |  | - |  | OM1.2 |  | 0.03255 |  | 0.0156 |  | 36.0 |  | 2.0906 |  | 0.804 |  |
|  |  | - |  | OM1.1 |  | 0.14098 |  | 0.0156 |  | 36.0 |  | 9.0561 |  | < .001 |  |
| OM2.3 |  | - |  | AC2.1 |  | 0.00785 |  | 0.0156 |  | 36.0 |  | 0.5041 |  | 1.000 |  |
|  |  | - |  | AC2.2 |  | -0.01920 |  | 0.0156 |  | 36.0 |  | -1.2332 |  | 0.998 |  |
|  |  | - |  | AC2.3 |  | 0.11148 |  | 0.0156 |  | 36.0 |  | 7.1610 |  | < .001 |  |
|  |  | - |  | CZ1.3 |  | 0.04829 |  | 0.0156 |  | 36.0 |  | 3.1018 |  | 0.208 |  |
|  |  | - |  | CZ1.4 |  | 0.08319 |  | 0.0156 |  | 36.0 |  | 5.3439 |  | < .001 |  |
|  |  | - |  | OM1.2 |  | 0.01549 |  | 0.0156 |  | 36.0 |  | 0.9949 |  | 1.000 |  |
|  |  | - |  | OM1.1 |  | 0.12393 |  | 0.0156 |  | 36.0 |  | 7.9605 |  | < .001 |  |
| AC2.1 |  | - |  | AC2.2 |  | -0.02704 |  | 0.0156 |  | 36.0 |  | -1.7372 |  | 0.945 |  |
|  |  | - |  | AC2.3 |  | 0.10363 |  | 0.0156 |  | 36.0 |  | 6.6569 |  | < .001 |  |
|  |  | - |  | CZ1.3 |  | 0.04044 |  | 0.0156 |  | 36.0 |  | 2.5978 |  | 0.480 |  |
|  |  | - |  | CZ1.4 |  | 0.07535 |  | 0.0156 |  | 36.0 |  | 4.8399 |  | 0.003 |  |
|  |  | - |  | OM1.2 |  | 0.00764 |  | 0.0156 |  | 36.0 |  | 0.4909 |  | 1.000 |  |
|  |  | - |  | OM1.1 |  | 0.11608 |  | 0.0156 |  | 36.0 |  | 7.4564 |  | < .001 |  |
| AC2.2 |  | - |  | AC2.3 |  | 0.13068 |  | 0.0156 |  | 36.0 |  | 8.3941 |  | < .001 |  |
|  |  | - |  | CZ1.3 |  | 0.06749 |  | 0.0156 |  | 36.0 |  | 4.3350 |  | 0.011 |  |
|  |  | - |  | CZ1.4 |  | 0.10239 |  | 0.0156 |  | 36.0 |  | 6.5771 |  | < .001 |  |
|  |  | - |  | OM1.2 |  | 0.03469 |  | 0.0156 |  | 36.0 |  | 2.2281 |  | 0.724 |  |
|  |  | - |  | OM1.1 |  | 0.14312 |  | 0.0156 |  | 36.0 |  | 9.1936 |  | < .001 |  |
| AC2.3 |  | - |  | CZ1.3 |  | -0.06319 |  | 0.0156 |  | 36.0 |  | -4.0591 |  | 0.023 |  |
|  |  | - |  | CZ1.4 |  | -0.02829 |  | 0.0156 |  | 36.0 |  | -1.8170 |  | 0.923 |  |
|  |  | - |  | OM1.2 |  | -0.09599 |  | 0.0156 |  | 36.0 |  | -6.1660 |  | < .001 |  |
|  |  | - |  | OM1.1 |  | 0.01245 |  | 0.0156 |  | 36.0 |  | 0.7995 |  | 1.000 |  |
| CZ1.3 |  | - |  | CZ1.4 |  | 0.03490 |  | 0.0156 |  | 36.0 |  | 2.2421 |  | 0.715 |  |
|  |  | - |  | OM1.2 |  | -0.03280 |  | 0.0156 |  | 36.0 |  | -2.1069 |  | 0.795 |  |
|  |  | - |  | OM1.1 |  | 0.07564 |  | 0.0156 |  | 36.0 |  | 4.8586 |  | 0.003 |  |
| CZ1.4 |  | - |  | OM1.2 |  | -0.06770 |  | 0.0156 |  | 36.0 |  | -4.3490 |  | 0.011 |  |
|  |  | - |  | OM1.1 |  | 0.04073 |  | 0.0156 |  | 36.0 |  | 2.6165 |  | 0.468 |  |
| OM1.2 |  | - |  | OM1.1 |  | 0.10844 |  | 0.0156 |  | 36.0 |  | 6.9655 |  | < .001 |  |
| Note. Comparisons are based on estimated marginal means | | | | | | | | | | | | | | | |
|  | | | | | | | | | | | | | | | |

| **CMCase** Estimated Marginal Means - Isolate Code | | | | | | | | | |
| --- | --- | --- | --- | --- | --- | --- | --- | --- | --- |
|  | | | | | | **95% Confidence Interval** | | | |
| **Isolate Code** | | **Mean** | | **SE** | | **Lower** | | **Upper** | |
| AC1.4 |  | 0.1199 |  | 0.0110 |  | 0.0976 |  | 0.142 |  |
| AC1.6 |  | 0.2298 |  | 0.0110 |  | 0.2075 |  | 0.252 |  |
| CZ1.1 |  | 0.0991 |  | 0.0110 |  | 0.0768 |  | 0.121 |  |
| CZ1.2 |  | 0.1407 |  | 0.0110 |  | 0.1184 |  | 0.163 |  |
| AC1.1 |  | 0.2064 |  | 0.0110 |  | 0.1841 |  | 0.229 |  |
| AC1.2 |  | 0.1472 |  | 0.0110 |  | 0.1248 |  | 0.169 |  |
| AC1.3 |  | 0.1553 |  | 0.0110 |  | 0.1330 |  | 0.178 |  |
| AC1.5 |  | 0.1378 |  | 0.0110 |  | 0.1155 |  | 0.160 |  |
| OM2.1 |  | 0.1557 |  | 0.0110 |  | 0.1334 |  | 0.178 |  |
| OM2.2 |  | 0.2790 |  | 0.0110 |  | 0.2567 |  | 0.301 |  |
| OM2.3 |  | 0.2619 |  | 0.0110 |  | 0.2396 |  | 0.284 |  |
| AC2.1 |  | 0.2541 |  | 0.0110 |  | 0.2318 |  | 0.276 |  |
| AC2.2 |  | 0.2811 |  | 0.0110 |  | 0.2588 |  | 0.303 |  |
| AC2.3 |  | 0.1505 |  | 0.0110 |  | 0.1281 |  | 0.173 |  |
| CZ1.3 |  | 0.2137 |  | 0.0110 |  | 0.1913 |  | 0.236 |  |
| CZ1.4 |  | 0.1788 |  | 0.0110 |  | 0.1564 |  | 0.201 |  |
| OM1.2 |  | 0.2465 |  | 0.0110 |  | 0.2241 |  | 0.269 |  |
| OM1.1 |  | 0.1380 |  | 0.0110 |  | 0.1157 |  | 0.160 |  |
|  | | | | | | | | | |

| **CMCase** Post Hoc Comparisons - Environment | | | | | | | | | | | | | | | |
| --- | --- | --- | --- | --- | --- | --- | --- | --- | --- | --- | --- | --- | --- | --- | --- |
| **Comparison** | | | | | |  | | | | | | | | | |
| **Envi** | |  | | **Envi** | | **Mean Difference** | | **SE** | | **df** | | **t** | | **p_tukey_** | |
| TERRE |  | - |  | MAR |  | -0.0626 |  | 0.0150 |  | 52.0 |  | -4.18 |  | < .001 |  |
| Note. Comparisons are based on estimated marginal means | | | | | | | | | | | | | | | |
|  | | | | | | | | | | | | | | | |

| **CMCase** Estimated Marginal Means - Environment | | | | | | | | | |
| --- | --- | --- | --- | --- | --- | --- | --- | --- | --- |
|  | | | | | | **95% Confidence Interval** | | | |
| **Envi** | | **Mean** | | **SE** | | **Lower** | | **Upper** | |
| TERRE |  | 0.168 |  | 0.00865 |  | 0.150 |  | 0.185 |  |
| MAR |  | 0.230 |  | 0.01223 |  | 0.206 |  | 0.255 |  |
|  | | | | | | | | | |

| **Avicelase** Post Hoc Comparisons - Isolate Code | | | | | | | | | | | | | | | |
| --- | --- | --- | --- | --- | --- | --- | --- | --- | --- | --- | --- | --- | --- | --- | --- |
| **Comparison** | | | | | |  | | | | | | | | | |
| **Isolate Code** | |  | | **Isolate Code** | | **Mean Difference** | | **SE** | | **df** | | **t** | | **p_tukey_** | |
| AC1.4 |  | - |  | AC1.6 |  | -0.13967 |  | 0.0416 |  | 36.0 |  | -3.36042 |  | 0.123 |  |
|  |  | - |  | CZ1.1 |  | -0.03300 |  | 0.0416 |  | 36.0 |  | -0.79399 |  | 1.000 |  |
|  |  | - |  | CZ1.2 |  | -0.02867 |  | 0.0416 |  | 36.0 |  | -0.68973 |  | 1.000 |  |
|  |  | - |  | AC1.1 |  | -0.10067 |  | 0.0416 |  | 36.0 |  | -2.42207 |  | 0.597 |  |
|  |  | - |  | AC1.2 |  | -0.02467 |  | 0.0416 |  | 36.0 |  | -0.59349 |  | 1.000 |  |
|  |  | - |  | AC1.3 |  | -0.08933 |  | 0.0416 |  | 36.0 |  | -2.14939 |  | 0.771 |  |
|  |  | - |  | AC1.5 |  | -3.33e−4 |  | 0.0416 |  | 36.0 |  | -0.00802 |  | 1.000 |  |
|  |  | - |  | OM2.1 |  | -0.02833 |  | 0.0416 |  | 36.0 |  | -0.68171 |  | 1.000 |  |
|  |  | - |  | OM2.2 |  | -0.16767 |  | 0.0416 |  | 36.0 |  | -4.03411 |  | 0.025 |  |
|  |  | - |  | OM2.3 |  | -0.18633 |  | 0.0416 |  | 36.0 |  | -4.48324 |  | 0.007 |  |
|  |  | - |  | AC2.1 |  | -0.17133 |  | 0.0416 |  | 36.0 |  | -4.12233 |  | 0.019 |  |
|  |  | - |  | AC2.2 |  | -0.26733 |  | 0.0416 |  | 36.0 |  | -6.43212 |  | < .001 |  |
|  |  | - |  | AC2.3 |  | -0.10800 |  | 0.0416 |  | 36.0 |  | -2.59851 |  | 0.479 |  |
|  |  | - |  | CZ1.3 |  | -0.06567 |  | 0.0416 |  | 36.0 |  | -1.57996 |  | 0.976 |  |
|  |  | - |  | CZ1.4 |  | -0.10600 |  | 0.0416 |  | 36.0 |  | -2.55039 |  | 0.511 |  |
|  |  | - |  | OM1.2 |  | -0.09200 |  | 0.0416 |  | 36.0 |  | -2.21355 |  | 0.733 |  |
|  |  | - |  | OM1.1 |  | -0.06933 |  | 0.0416 |  | 36.0 |  | -1.66818 |  | 0.961 |  |
| AC1.6 |  | - |  | CZ1.1 |  | 0.10667 |  | 0.0416 |  | 36.0 |  | 2.56643 |  | 0.501 |  |
|  |  | - |  | CZ1.2 |  | 0.11100 |  | 0.0416 |  | 36.0 |  | 2.67069 |  | 0.433 |  |
|  |  | - |  | AC1.1 |  | 0.03900 |  | 0.0416 |  | 36.0 |  | 0.93835 |  | 1.000 |  |
|  |  | - |  | AC1.2 |  | 0.11500 |  | 0.0416 |  | 36.0 |  | 2.76693 |  | 0.375 |  |
|  |  | - |  | AC1.3 |  | 0.05033 |  | 0.0416 |  | 36.0 |  | 1.21104 |  | 0.998 |  |
|  |  | - |  | AC1.5 |  | 0.13933 |  | 0.0416 |  | 36.0 |  | 3.35240 |  | 0.125 |  |
|  |  | - |  | OM2.1 |  | 0.11133 |  | 0.0416 |  | 36.0 |  | 2.67871 |  | 0.428 |  |
|  |  | - |  | OM2.2 |  | -0.02800 |  | 0.0416 |  | 36.0 |  | -0.67369 |  | 1.000 |  |
|  |  | - |  | OM2.3 |  | -0.04667 |  | 0.0416 |  | 36.0 |  | -1.12281 |  | 0.999 |  |
|  |  | - |  | AC2.1 |  | -0.03167 |  | 0.0416 |  | 36.0 |  | -0.76191 |  | 1.000 |  |
|  |  | - |  | AC2.2 |  | -0.12767 |  | 0.0416 |  | 36.0 |  | -3.07170 |  | 0.221 |  |
|  |  | - |  | AC2.3 |  | 0.03167 |  | 0.0416 |  | 36.0 |  | 0.76191 |  | 1.000 |  |
|  |  | - |  | CZ1.3 |  | 0.07400 |  | 0.0416 |  | 36.0 |  | 1.78046 |  | 0.934 |  |
|  |  | - |  | CZ1.4 |  | 0.03367 |  | 0.0416 |  | 36.0 |  | 0.81003 |  | 1.000 |  |
|  |  | - |  | OM1.2 |  | 0.04767 |  | 0.0416 |  | 36.0 |  | 1.14687 |  | 0.999 |  |
|  |  | - |  | OM1.1 |  | 0.07033 |  | 0.0416 |  | 36.0 |  | 1.69224 |  | 0.956 |  |
| CZ1.1 |  | - |  | CZ1.2 |  | 0.00433 |  | 0.0416 |  | 36.0 |  | 0.10426 |  | 1.000 |  |
|  |  | - |  | AC1.1 |  | -0.06767 |  | 0.0416 |  | 36.0 |  | -1.62808 |  | 0.968 |  |
|  |  | - |  | AC1.2 |  | 0.00833 |  | 0.0416 |  | 36.0 |  | 0.20050 |  | 1.000 |  |
|  |  | - |  | AC1.3 |  | -0.05633 |  | 0.0416 |  | 36.0 |  | -1.35540 |  | 0.995 |  |
|  |  | - |  | AC1.5 |  | 0.03267 |  | 0.0416 |  | 36.0 |  | 0.78597 |  | 1.000 |  |
|  |  | - |  | OM2.1 |  | 0.00467 |  | 0.0416 |  | 36.0 |  | 0.11228 |  | 1.000 |  |
|  |  | - |  | OM2.2 |  | -0.13467 |  | 0.0416 |  | 36.0 |  | -3.24012 |  | 0.158 |  |
|  |  | - |  | OM2.3 |  | -0.15333 |  | 0.0416 |  | 36.0 |  | -3.68925 |  | 0.058 |  |
|  |  | - |  | AC2.1 |  | -0.13833 |  | 0.0416 |  | 36.0 |  | -3.32834 |  | 0.131 |  |
|  |  | - |  | AC2.2 |  | -0.23433 |  | 0.0416 |  | 36.0 |  | -5.63813 |  | < .001 |  |
|  |  | - |  | AC2.3 |  | -0.07500 |  | 0.0416 |  | 36.0 |  | -1.80452 |  | 0.927 |  |
|  |  | - |  | CZ1.3 |  | -0.03267 |  | 0.0416 |  | 36.0 |  | -0.78597 |  | 1.000 |  |
|  |  | - |  | CZ1.4 |  | -0.07300 |  | 0.0416 |  | 36.0 |  | -1.75640 |  | 0.941 |  |
|  |  | - |  | OM1.2 |  | -0.05900 |  | 0.0416 |  | 36.0 |  | -1.41956 |  | 0.991 |  |
|  |  | - |  | OM1.1 |  | -0.03633 |  | 0.0416 |  | 36.0 |  | -0.87419 |  | 1.000 |  |
| CZ1.2 |  | - |  | AC1.1 |  | -0.07200 |  | 0.0416 |  | 36.0 |  | -1.73234 |  | 0.947 |  |
|  |  | - |  | AC1.2 |  | 0.00400 |  | 0.0416 |  | 36.0 |  | 0.09624 |  | 1.000 |  |
|  |  | - |  | AC1.3 |  | -0.06067 |  | 0.0416 |  | 36.0 |  | -1.45966 |  | 0.989 |  |
|  |  | - |  | AC1.5 |  | 0.02833 |  | 0.0416 |  | 36.0 |  | 0.68171 |  | 1.000 |  |
|  |  | - |  | OM2.1 |  | 3.33e-4 |  | 0.0416 |  | 36.0 |  | 0.00802 |  | 1.000 |  |
|  |  | - |  | OM2.2 |  | -0.13900 |  | 0.0416 |  | 36.0 |  | -3.34438 |  | 0.127 |  |
|  |  | - |  | OM2.3 |  | -0.15767 |  | 0.0416 |  | 36.0 |  | -3.79351 |  | 0.045 |  |
|  |  | - |  | AC2.1 |  | -0.14267 |  | 0.0416 |  | 36.0 |  | -3.43260 |  | 0.105 |  |
|  |  | - |  | AC2.2 |  | -0.23867 |  | 0.0416 |  | 36.0 |  | -5.74239 |  | < .001 |  |
|  |  | - |  | AC2.3 |  | -0.07933 |  | 0.0416 |  | 36.0 |  | -1.90878 |  | 0.890 |  |
|  |  | - |  | CZ1.3 |  | -0.03700 |  | 0.0416 |  | 36.0 |  | -0.89023 |  | 1.000 |  |
|  |  | - |  | CZ1.4 |  | -0.07733 |  | 0.0416 |  | 36.0 |  | -1.86066 |  | 0.908 |  |
|  |  | - |  | OM1.2 |  | -0.06333 |  | 0.0416 |  | 36.0 |  | -1.52382 |  | 0.983 |  |
|  |  | - |  | OM1.1 |  | -0.04067 |  | 0.0416 |  | 36.0 |  | -0.97845 |  | 1.000 |  |
| AC1.1 |  | - |  | AC1.2 |  | 0.07600 |  | 0.0416 |  | 36.0 |  | 1.82858 |  | 0.919 |  |
|  |  | - |  | AC1.3 |  | 0.01133 |  | 0.0416 |  | 36.0 |  | 0.27268 |  | 1.000 |  |
|  |  | - |  | AC1.5 |  | 0.10033 |  | 0.0416 |  | 36.0 |  | 2.41405 |  | 0.602 |  |
|  |  | - |  | OM2.1 |  | 0.07233 |  | 0.0416 |  | 36.0 |  | 1.74036 |  | 0.945 |  |
|  |  | - |  | OM2.2 |  | -0.06700 |  | 0.0416 |  | 36.0 |  | -1.61204 |  | 0.971 |  |
|  |  | - |  | OM2.3 |  | -0.08567 |  | 0.0416 |  | 36.0 |  | -2.06117 |  | 0.820 |  |
|  |  | - |  | AC2.1 |  | -0.07067 |  | 0.0416 |  | 36.0 |  | -1.70026 |  | 0.954 |  |
|  |  | - |  | AC2.2 |  | -0.16667 |  | 0.0416 |  | 36.0 |  | -4.01005 |  | 0.026 |  |
|  |  | - |  | AC2.3 |  | -0.00733 |  | 0.0416 |  | 36.0 |  | -0.17644 |  | 1.000 |  |
|  |  | - |  | CZ1.3 |  | 0.03500 |  | 0.0416 |  | 36.0 |  | 0.84211 |  | 1.000 |  |
|  |  | - |  | CZ1.4 |  | -0.00533 |  | 0.0416 |  | 36.0 |  | -0.12832 |  | 1.000 |  |
|  |  | - |  | OM1.2 |  | 0.00867 |  | 0.0416 |  | 36.0 |  | 0.20852 |  | 1.000 |  |
|  |  | - |  | OM1.1 |  | 0.03133 |  | 0.0416 |  | 36.0 |  | 0.75389 |  | 1.000 |  |
| AC1.2 |  | - |  | AC1.3 |  | -0.06467 |  | 0.0416 |  | 36.0 |  | -1.55590 |  | 0.979 |  |
|  |  | - |  | AC1.5 |  | 0.02433 |  | 0.0416 |  | 36.0 |  | 0.58547 |  | 1.000 |  |
|  |  | - |  | OM2.1 |  | -0.00367 |  | 0.0416 |  | 36.0 |  | -0.08822 |  | 1.000 |  |
|  |  | - |  | OM2.2 |  | -0.14300 |  | 0.0416 |  | 36.0 |  | -3.44062 |  | 0.103 |  |
|  |  | - |  | OM2.3 |  | -0.16167 |  | 0.0416 |  | 36.0 |  | -3.88975 |  | 0.035 |  |
|  |  | - |  | AC2.1 |  | -0.14667 |  | 0.0416 |  | 36.0 |  | -3.52884 |  | 0.084 |  |
|  |  | - |  | AC2.2 |  | -0.24267 |  | 0.0416 |  | 36.0 |  | -5.83863 |  | < .001 |  |
|  |  | - |  | AC2.3 |  | -0.08333 |  | 0.0416 |  | 36.0 |  | -2.00503 |  | 0.848 |  |
|  |  | - |  | CZ1.3 |  | -0.04100 |  | 0.0416 |  | 36.0 |  | -0.98647 |  | 1.000 |  |
|  |  | - |  | CZ1.4 |  | -0.08133 |  | 0.0416 |  | 36.0 |  | -1.95690 |  | 0.870 |  |
|  |  | - |  | OM1.2 |  | -0.06733 |  | 0.0416 |  | 36.0 |  | -1.62006 |  | 0.970 |  |
|  |  | - |  | OM1.1 |  | -0.04467 |  | 0.0416 |  | 36.0 |  | -1.07469 |  | 1.000 |  |
| AC1.3 |  | - |  | AC1.5 |  | 0.08900 |  | 0.0416 |  | 36.0 |  | 2.14137 |  | 0.776 |  |
|  |  | - |  | OM2.1 |  | 0.06100 |  | 0.0416 |  | 36.0 |  | 1.46768 |  | 0.988 |  |
|  |  | - |  | OM2.2 |  | -0.07833 |  | 0.0416 |  | 36.0 |  | -1.88472 |  | 0.899 |  |
|  |  | - |  | OM2.3 |  | -0.09700 |  | 0.0416 |  | 36.0 |  | -2.33385 |  | 0.656 |  |
|  |  | - |  | AC2.1 |  | -0.08200 |  | 0.0416 |  | 36.0 |  | -1.97294 |  | 0.863 |  |
|  |  | - |  | AC2.2 |  | -0.17800 |  | 0.0416 |  | 36.0 |  | -4.28273 |  | 0.013 |  |
|  |  | - |  | AC2.3 |  | -0.01867 |  | 0.0416 |  | 36.0 |  | -0.44913 |  | 1.000 |  |
|  |  | - |  | CZ1.3 |  | 0.02367 |  | 0.0416 |  | 36.0 |  | 0.56943 |  | 1.000 |  |
|  |  | - |  | CZ1.4 |  | -0.01667 |  | 0.0416 |  | 36.0 |  | -0.40101 |  | 1.000 |  |
|  |  | - |  | OM1.2 |  | -0.00267 |  | 0.0416 |  | 36.0 |  | -0.06416 |  | 1.000 |  |
|  |  | - |  | OM1.1 |  | 0.02000 |  | 0.0416 |  | 36.0 |  | 0.48121 |  | 1.000 |  |
| AC1.5 |  | - |  | OM2.1 |  | -0.02800 |  | 0.0416 |  | 36.0 |  | -0.67369 |  | 1.000 |  |
|  |  | - |  | OM2.2 |  | -0.16733 |  | 0.0416 |  | 36.0 |  | -4.02609 |  | 0.025 |  |
|  |  | - |  | OM2.3 |  | -0.18600 |  | 0.0416 |  | 36.0 |  | -4.47522 |  | 0.008 |  |
|  |  | - |  | AC2.1 |  | -0.17100 |  | 0.0416 |  | 36.0 |  | -4.11431 |  | 0.020 |  |
|  |  | - |  | AC2.2 |  | -0.26700 |  | 0.0416 |  | 36.0 |  | -6.42410 |  | < .001 |  |
|  |  | - |  | AC2.3 |  | -0.10767 |  | 0.0416 |  | 36.0 |  | -2.59049 |  | 0.485 |  |
|  |  | - |  | CZ1.3 |  | -0.06533 |  | 0.0416 |  | 36.0 |  | -1.57194 |  | 0.977 |  |
|  |  | - |  | CZ1.4 |  | -0.10567 |  | 0.0416 |  | 36.0 |  | -2.54237 |  | 0.516 |  |
|  |  | - |  | OM1.2 |  | -0.09167 |  | 0.0416 |  | 36.0 |  | -2.20553 |  | 0.738 |  |
|  |  | - |  | OM1.1 |  | -0.06900 |  | 0.0416 |  | 36.0 |  | -1.66016 |  | 0.962 |  |
| OM2.1 |  | - |  | OM2.2 |  | -0.13933 |  | 0.0416 |  | 36.0 |  | -3.35240 |  | 0.125 |  |
|  |  | - |  | OM2.3 |  | -0.15800 |  | 0.0416 |  | 36.0 |  | -3.80153 |  | 0.044 |  |
|  |  | - |  | AC2.1 |  | -0.14300 |  | 0.0416 |  | 36.0 |  | -3.44062 |  | 0.103 |  |
|  |  | - |  | AC2.2 |  | -0.23900 |  | 0.0416 |  | 36.0 |  | -5.75041 |  | < .001 |  |
|  |  | - |  | AC2.3 |  | -0.07967 |  | 0.0416 |  | 36.0 |  | -1.91680 |  | 0.887 |  |
|  |  | - |  | CZ1.3 |  | -0.03733 |  | 0.0416 |  | 36.0 |  | -0.89825 |  | 1.000 |  |
|  |  | - |  | CZ1.4 |  | -0.07767 |  | 0.0416 |  | 36.0 |  | -1.86868 |  | 0.905 |  |
|  |  | - |  | OM1.2 |  | -0.06367 |  | 0.0416 |  | 36.0 |  | -1.53184 |  | 0.982 |  |
|  |  | - |  | OM1.1 |  | -0.04100 |  | 0.0416 |  | 36.0 |  | -0.98647 |  | 1.000 |  |
| OM2.2 |  | - |  | OM2.3 |  | -0.01867 |  | 0.0416 |  | 36.0 |  | -0.44913 |  | 1.000 |  |
|  |  | - |  | AC2.1 |  | -0.00367 |  | 0.0416 |  | 36.0 |  | -0.08822 |  | 1.000 |  |
|  |  | - |  | AC2.2 |  | -0.09967 |  | 0.0416 |  | 36.0 |  | -2.39801 |  | 0.613 |  |
|  |  | - |  | AC2.3 |  | 0.05967 |  | 0.0416 |  | 36.0 |  | 1.43560 |  | 0.990 |  |
|  |  | - |  | CZ1.3 |  | 0.10200 |  | 0.0416 |  | 36.0 |  | 2.45415 |  | 0.576 |  |
|  |  | - |  | CZ1.4 |  | 0.06167 |  | 0.0416 |  | 36.0 |  | 1.48372 |  | 0.987 |  |
|  |  | - |  | OM1.2 |  | 0.07567 |  | 0.0416 |  | 36.0 |  | 1.82056 |  | 0.922 |  |
|  |  | - |  | OM1.1 |  | 0.09833 |  | 0.0416 |  | 36.0 |  | 2.36593 |  | 0.635 |  |
| OM2.3 |  | - |  | AC2.1 |  | 0.01500 |  | 0.0416 |  | 36.0 |  | 0.36090 |  | 1.000 |  |
|  |  | - |  | AC2.2 |  | -0.08100 |  | 0.0416 |  | 36.0 |  | -1.94888 |  | 0.873 |  |
|  |  | - |  | AC2.3 |  | 0.07833 |  | 0.0416 |  | 36.0 |  | 1.88472 |  | 0.899 |  |
|  |  | - |  | CZ1.3 |  | 0.12067 |  | 0.0416 |  | 36.0 |  | 2.90328 |  | 0.299 |  |
|  |  | - |  | CZ1.4 |  | 0.08033 |  | 0.0416 |  | 36.0 |  | 1.93284 |  | 0.880 |  |
|  |  | - |  | OM1.2 |  | 0.09433 |  | 0.0416 |  | 36.0 |  | 2.26969 |  | 0.698 |  |
|  |  | - |  | OM1.1 |  | 0.11700 |  | 0.0416 |  | 36.0 |  | 2.81506 |  | 0.347 |  |
| AC2.1 |  | - |  | AC2.2 |  | -0.09600 |  | 0.0416 |  | 36.0 |  | -2.30979 |  | 0.672 |  |
|  |  | - |  | AC2.3 |  | 0.06333 |  | 0.0416 |  | 36.0 |  | 1.52382 |  | 0.983 |  |
|  |  | - |  | CZ1.3 |  | 0.10567 |  | 0.0416 |  | 36.0 |  | 2.54237 |  | 0.516 |  |
|  |  | - |  | CZ1.4 |  | 0.06533 |  | 0.0416 |  | 36.0 |  | 1.57194 |  | 0.977 |  |
|  |  | - |  | OM1.2 |  | 0.07933 |  | 0.0416 |  | 36.0 |  | 1.90878 |  | 0.890 |  |
|  |  | - |  | OM1.1 |  | 0.10200 |  | 0.0416 |  | 36.0 |  | 2.45415 |  | 0.576 |  |
| AC2.2 |  | - |  | AC2.3 |  | 0.15933 |  | 0.0416 |  | 36.0 |  | 3.83361 |  | 0.041 |  |
|  |  | - |  | CZ1.3 |  | 0.20167 |  | 0.0416 |  | 36.0 |  | 4.85216 |  | 0.003 |  |
|  |  | - |  | CZ1.4 |  | 0.16133 |  | 0.0416 |  | 36.0 |  | 3.88173 |  | 0.036 |  |
|  |  | - |  | OM1.2 |  | 0.17533 |  | 0.0416 |  | 36.0 |  | 4.21857 |  | 0.015 |  |
|  |  | - |  | OM1.1 |  | 0.19800 |  | 0.0416 |  | 36.0 |  | 4.76394 |  | 0.003 |  |
| AC2.3 |  | - |  | CZ1.3 |  | 0.04233 |  | 0.0416 |  | 36.0 |  | 1.01855 |  | 1.000 |  |
|  |  | - |  | CZ1.4 |  | 0.00200 |  | 0.0416 |  | 36.0 |  | 0.04812 |  | 1.000 |  |
|  |  | - |  | OM1.2 |  | 0.01600 |  | 0.0416 |  | 36.0 |  | 0.38496 |  | 1.000 |  |
|  |  | - |  | OM1.1 |  | 0.03867 |  | 0.0416 |  | 36.0 |  | 0.93033 |  | 1.000 |  |
| CZ1.3 |  | - |  | CZ1.4 |  | -0.04033 |  | 0.0416 |  | 36.0 |  | -0.97043 |  | 1.000 |  |
|  |  | - |  | OM1.2 |  | -0.02633 |  | 0.0416 |  | 36.0 |  | -0.63359 |  | 1.000 |  |
|  |  | - |  | OM1.1 |  | -0.00367 |  | 0.0416 |  | 36.0 |  | -0.08822 |  | 1.000 |  |
| CZ1.4 |  | - |  | OM1.2 |  | 0.01400 |  | 0.0416 |  | 36.0 |  | 0.33684 |  | 1.000 |  |
|  |  | - |  | OM1.1 |  | 0.03667 |  | 0.0416 |  | 36.0 |  | 0.88221 |  | 1.000 |  |
| OM1.2 |  | - |  | OM1.1 |  | 0.02267 |  | 0.0416 |  | 36.0 |  | 0.54537 |  | 1.000 |  |
| Note. Comparisons are based on estimated marginal means | | | | | | | | | | | | | | | |
|  | | | | | | | | | | | | | | | |

| **Avicelase** Estimated Marginal Means - Isolate Code | | | | | | | | | |
| --- | --- | --- | --- | --- | --- | --- | --- | --- | --- |
|  | | | | | | **95% Confidence Interval** | | | |
| **Isolate Code** | | **Mean** | | **SE** | | **Lower** | | **Upper** | |
| AC1.4 |  | 0.103 |  | 0.0294 |  | 0.0434 |  | 0.163 |  |
| AC1.6 |  | 0.243 |  | 0.0294 |  | 0.1831 |  | 0.302 |  |
| CZ1.1 |  | 0.136 |  | 0.0294 |  | 0.0764 |  | 0.196 |  |
| CZ1.2 |  | 0.132 |  | 0.0294 |  | 0.0721 |  | 0.191 |  |
| AC1.1 |  | 0.204 |  | 0.0294 |  | 0.1441 |  | 0.263 |  |
| AC1.2 |  | 0.128 |  | 0.0294 |  | 0.0681 |  | 0.187 |  |
| AC1.3 |  | 0.192 |  | 0.0294 |  | 0.1327 |  | 0.252 |  |
| AC1.5 |  | 0.103 |  | 0.0294 |  | 0.0437 |  | 0.163 |  |
| OM2.1 |  | 0.131 |  | 0.0294 |  | 0.0717 |  | 0.191 |  |
| OM2.2 |  | 0.271 |  | 0.0294 |  | 0.2111 |  | 0.330 |  |
| OM2.3 |  | 0.289 |  | 0.0294 |  | 0.2297 |  | 0.349 |  |
| AC2.1 |  | 0.274 |  | 0.0294 |  | 0.2147 |  | 0.334 |  |
| AC2.2 |  | 0.370 |  | 0.0294 |  | 0.3107 |  | 0.430 |  |
| AC2.3 |  | 0.211 |  | 0.0294 |  | 0.1514 |  | 0.271 |  |
| CZ1.3 |  | 0.169 |  | 0.0294 |  | 0.1091 |  | 0.228 |  |
| CZ1.4 |  | 0.209 |  | 0.0294 |  | 0.1494 |  | 0.269 |  |
| OM1.2 |  | 0.195 |  | 0.0294 |  | 0.1354 |  | 0.255 |  |
| OM1.1 |  | 0.172 |  | 0.0294 |  | 0.1127 |  | 0.232 |  |
|  | | | | | | | | | |

| **Avicelase** Post Hoc Comparisons - Environment | | | | | | | | | | | | | | | |
| --- | --- | --- | --- | --- | --- | --- | --- | --- | --- | --- | --- | --- | --- | --- | --- |
| **Comparison** | | | | | |  | | | | | | | | | |
| **Envi** | |  | | **Envi** | | **Mean Difference** | | **SE** | | **df** | | **t** | | **p_tukey_** | |
| TERRE |  | - |  | MAR |  | -0.0924 |  | 0.0203 |  | 52.0 |  | -4.56 |  | < .001 |  |
| Note. Comparisons are based on estimated marginal means | | | | | | | | | | | | | | | |
|  | | | | | | | | | | | | | | | |

| **Avicelase** Estimated Marginal Means - Environment | | | | | | | | | |
| --- | --- | --- | --- | --- | --- | --- | --- | --- | --- |
|  | | | | | | **95% Confidence Interval** | | | |
| **Envi** | | **Mean** | | **SE** | | **Lower** | | **Upper** | |
| TERRE |  | 0.165 |  | 0.0117 |  | 0.142 |  | 0.189 |  |
| MAR |  | 0.258 |  | 0.0166 |  | 0.225 |  | 0.291 |  |
|  | | | | | | | | | |

| **FPase** Post Hoc Comparisons - Isolate Code | | | | | | | | | | | | | | | | | | | | | | | |
| --- | --- | --- | --- | --- | --- | --- | --- | --- | --- | --- | --- | --- | --- | --- | --- | --- | --- | --- | --- | --- | --- | --- | --- |
| **Comparison** | | | | | | | | | |  | | | | | | | | | | | | | |
| **Isolate Code** | |  | | **Isolate Code** | | | | | | **Mean Difference** | | | | **SE** | | | | **df** | | **t** | | **p_tukey_** | |
| AC1.4 |  | - |  | AC1.6 | | |  | | | 0.07233 | | |  | 0.0178 | | |  | 36.0 |  | 4.0581 |  | 0.023 |  |
|  |  | - |  | CZ1.1 | | |  | | | 0.07733 | | |  | 0.0178 | | |  | 36.0 |  | 4.3386 |  | 0.011 |  |
|  |  | - |  | CZ1.2 | | |  | | | 0.09400 | | |  | 0.0178 | | |  | 36.0 |  | 5.2736 |  | < .001 |  |
|  |  | - |  | AC1.1 | | |  | | | -0.03933 | | |  | 0.0178 | | |  | 36.0 |  | -2.2067 |  | 0.737 |  |
|  |  | - |  | AC1.2 | | |  | | | 0.06500 | | |  | 0.0178 | | |  | 36.0 |  | 3.6466 |  | 0.064 |  |
|  |  | - |  | AC1.3 | | |  | | | -0.16900 | | |  | 0.0178 | | |  | 36.0 |  | -9.4813 |  | < .001 |  |
|  |  | - |  | AC1.5 | | |  | | | -0.07233 | | |  | 0.0178 | | |  | 36.0 |  | -4.0581 |  | 0.023 |  |
|  |  | - |  | OM2.1 | | |  | | | -0.00433 | | |  | 0.0178 | | |  | 36.0 |  | -0.2431 |  | 1.000 |  |
|  |  | - |  | OM2.2 | | |  | | | -0.04467 | | |  | 0.0178 | | |  | 36.0 |  | -2.5059 |  | 0.541 |  |
|  |  | - |  | OM2.3 | | |  | | | -0.01667 | | |  | 0.0178 | | |  | 36.0 |  | -0.9350 |  | 1.000 |  |
|  |  | - |  | AC2.1 | | |  | | | 0.07467 | | |  | 0.0178 | | |  | 36.0 |  | 4.1890 |  | 0.016 |  |
|  |  | - |  | AC2.2 | | |  | | | 0.05633 | | |  | 0.0178 | | |  | 36.0 |  | 3.1604 |  | 0.186 |  |
|  |  | - |  | AC2.3 | | |  | | | 0.06700 | | |  | 0.0178 | | |  | 36.0 |  | 3.7589 |  | 0.049 |  |
|  |  | - |  | CZ1.3 | | |  | | | 0.00400 | | |  | 0.0178 | | |  | 36.0 |  | 0.2244 |  | 1.000 |  |
|  |  | - |  | CZ1.4 | | |  | | | 0.05533 | | |  | 0.0178 | | |  | 36.0 |  | 3.1043 |  | 0.207 |  |
|  |  | - |  | OM1.2 | | |  | | | 0.02000 | | |  | 0.0178 | | |  | 36.0 |  | 1.1220 |  | 0.999 |  |
|  |  | - |  | OM1.1 | | |  | | | 0.05367 | | |  | 0.0178 | | |  | 36.0 |  | 3.0108 |  | 0.247 |  |
| AC1.6 |  | - |  | CZ1.1 | | |  | | | 0.00500 | | |  | 0.0178 | | |  | 36.0 |  | 0.2805 |  | 1.000 |  |
|  |  | - |  | CZ1.2 | | |  | | | 0.02167 | | |  | 0.0178 | | |  | 36.0 |  | 1.2155 |  | 0.998 |  |
|  |  | - |  | AC1.1 | | |  | | | -0.11167 | | |  | 0.0178 | | |  | 36.0 |  | -6.2648 |  | < .001 |  |
|  |  | - |  | AC1.2 | | |  | | | -0.00733 | | |  | 0.0178 | | |  | 36.0 |  | -0.4114 |  | 1.000 |  |
|  |  | - |  | AC1.3 | | |  | | | -0.24133 | | |  | 0.0178 | | |  | 36.0 |  | -13.5393 |  | < .001 |  |
|  |  | - |  | AC1.5 | | |  | | | -0.14467 | | |  | 0.0178 | | |  | 36.0 |  | -8.1161 |  | < .001 |  |
|  |  | - |  | OM2.1 | | |  | | | -0.07667 | | |  | 0.0178 | | |  | 36.0 |  | -4.3012 |  | 0.012 |  |
|  |  | - |  | OM2.2 | | |  | | | -0.11700 | | |  | 0.0178 | | |  | 36.0 |  | -6.5640 |  | < .001 |  |
|  |  | - |  | OM2.3 | | |  | | | -0.08900 | | |  | 0.0178 | | |  | 36.0 |  | -4.9931 |  | 0.002 |  |
|  |  | - |  | AC2.1 | | |  | | | 0.00233 | | |  | 0.0178 | | |  | 36.0 |  | 0.1309 |  | 1.000 |  |
|  |  | - |  | AC2.2 | | |  | | | -0.01600 | | |  | 0.0178 | | |  | 36.0 |  | -0.8976 |  | 1.000 |  |
|  |  | - |  | AC2.3 | | |  | | | -0.00533 | | |  | 0.0178 | | |  | 36.0 |  | -0.2992 |  | 1.000 |  |
|  |  | - |  | CZ1.3 | | |  | | | -0.06833 | | |  | 0.0178 | | |  | 36.0 |  | -3.8337 |  | 0.041 |  |
|  |  | - |  | CZ1.4 | | |  | | | -0.01700 | | |  | 0.0178 | | |  | 36.0 |  | -0.9537 |  | 1.000 |  |
|  |  | - |  | OM1.2 | | |  | | | -0.05233 | | |  | 0.0178 | | |  | 36.0 |  | -2.9360 |  | 0.283 |  |
|  |  | - |  | OM1.1 | | |  | | | -0.01867 | | |  | 0.0178 | | |  | 36.0 |  | -1.0472 |  | 1.000 |  |
| CZ1.1 |  | - |  | CZ1.2 | | |  | | | 0.01667 | | |  | 0.0178 | | |  | 36.0 |  | 0.9350 |  | 1.000 |  |
|  |  | - |  | AC1.1 | | |  | | | -0.11667 | | |  | 0.0178 | | |  | 36.0 |  | -6.5453 |  | < .001 |  |
|  |  | - |  | AC1.2 | | |  | | | -0.01233 | | |  | 0.0178 | | |  | 36.0 |  | -0.6919 |  | 1.000 |  |
|  |  | - |  | AC1.3 | | |  | | | -0.24633 | | |  | 0.0178 | | |  | 36.0 |  | -13.8199 |  | < .001 |  |
|  |  | - |  | AC1.5 | | |  | | | -0.14967 | | |  | 0.0178 | | |  | 36.0 |  | -8.3966 |  | < .001 |  |
|  |  | - |  | OM2.1 | | |  | | | -0.08167 | | |  | 0.0178 | | |  | 36.0 |  | -4.5817 |  | 0.006 |  |
|  |  | - |  | OM2.2 | | |  | | | -0.12200 | | |  | 0.0178 | | |  | 36.0 |  | -6.8445 |  | < .001 |  |
|  |  | - |  | OM2.3 | | |  | | | -0.09400 | | |  | 0.0178 | | |  | 36.0 |  | -5.2736 |  | < .001 |  |
|  |  | - |  | AC2.1 | | |  | | | -0.00267 | | |  | 0.0178 | | |  | 36.0 |  | -0.1496 |  | 1.000 |  |
|  |  | - |  | AC2.2 | | |  | | | -0.02100 | | |  | 0.0178 | | |  | 36.0 |  | -1.1781 |  | 0.999 |  |
|  |  | - |  | AC2.3 | | |  | | | -0.01033 | | |  | 0.0178 | | |  | 36.0 |  | -0.5797 |  | 1.000 |  |
|  |  | - |  | CZ1.3 | | |  | | | -0.07333 | | |  | 0.0178 | | |  | 36.0 |  | -4.1142 |  | 0.020 |  |
|  |  | - |  | CZ1.4 | | |  | | | -0.02200 | | |  | 0.0178 | | |  | 36.0 |  | -1.2342 |  | 0.998 |  |
|  |  | - |  | OM1.2 | | |  | | | -0.05733 | | |  | 0.0178 | | |  | 36.0 |  | -3.2165 |  | 0.166 |  |
|  |  | - |  | OM1.1 | | |  | | | -0.02367 | | |  | 0.0178 | | |  | 36.0 |  | -1.3278 |  | 0.996 |  |
| CZ1.2 |  | - |  | AC1.1 | | |  | | | -0.13333 | | |  | 0.0178 | | |  | 36.0 |  | -7.4803 |  | < .001 |  |
|  |  | - |  | AC1.2 | | |  | | | -0.02900 | | |  | 0.0178 | | |  | 36.0 |  | -1.6270 |  | 0.968 |  |
|  |  | - |  | AC1.3 | | |  | | | -0.26300 | | |  | 0.0178 | | |  | 36.0 |  | -14.7549 |  | < .001 |  |
|  |  | - |  | AC1.5 | | |  | | | -0.16633 | | |  | 0.0178 | | |  | 36.0 |  | -9.3317 |  | < .001 |  |
|  |  | - |  | OM2.1 | | |  | | | -0.09833 | | |  | 0.0178 | | |  | 36.0 |  | -5.5167 |  | < .001 |  |
|  |  | - |  | OM2.2 | | |  | | | -0.13867 | | |  | 0.0178 | | |  | 36.0 |  | -7.7795 |  | < .001 |  |
|  |  | - |  | OM2.3 | | |  | | | -0.11067 | | |  | 0.0178 | | |  | 36.0 |  | -6.2087 |  | < .001 |  |
|  |  | - |  | AC2.1 | | |  | | | -0.01933 | | |  | 0.0178 | | |  | 36.0 |  | -1.0846 |  | 1.000 |  |
|  |  | - |  | AC2.2 | | |  | | | -0.03767 | | |  | 0.0178 | | |  | 36.0 |  | -2.1132 |  | 0.792 |  |
|  |  | - |  | AC2.3 | | |  | | | -0.02700 | | |  | 0.0178 | | |  | 36.0 |  | -1.5148 |  | 0.984 |  |
|  |  | - |  | CZ1.3 | | |  | | | -0.09000 | | |  | 0.0178 | | |  | 36.0 |  | -5.0492 |  | 0.001 |  |
|  |  | - |  | CZ1.4 | | |  | | | -0.03867 | | |  | 0.0178 | | |  | 36.0 |  | -2.1693 |  | 0.760 |  |
|  |  | - |  | OM1.2 | | |  | | | -0.07400 | | |  | 0.0178 | | |  | 36.0 |  | -4.1516 |  | 0.018 |  |
|  |  | - |  | OM1.1 | | |  | | | -0.04033 | | |  | 0.0178 | | |  | 36.0 |  | -2.2628 |  | 0.702 |  |
| AC1.1 |  | - |  | AC1.2 | | |  | | | 0.10433 | | |  | 0.0178 | | |  | 36.0 |  | 5.8533 |  | < .001 |  |
|  |  | - |  | AC1.3 | | |  | | | -0.12967 | | |  | 0.0178 | | |  | 36.0 |  | -7.2746 |  | < .001 |  |
|  |  | - |  | AC1.5 | | |  | | | -0.03300 | | |  | 0.0178 | | |  | 36.0 |  | -1.8514 |  | 0.911 |  |
|  |  | - |  | OM2.1 | | |  | | | 0.03500 | | |  | 0.0178 | | |  | 36.0 |  | 1.9636 |  | 0.867 |  |
|  |  | - |  | OM2.2 | | |  | | | -0.00533 | | |  | 0.0178 | | |  | 36.0 |  | -0.2992 |  | 1.000 |  |
|  |  | - |  | OM2.3 | | |  | | | 0.02267 | | |  | 0.0178 | | |  | 36.0 |  | 1.2717 |  | 0.997 |  |
|  |  | - |  | AC2.1 | | |  | | | 0.11400 | | |  | 0.0178 | | |  | 36.0 |  | 6.3957 |  | < .001 |  |
|  |  | - |  | AC2.2 | | |  | | | 0.09567 | | |  | 0.0178 | | |  | 36.0 |  | 5.3671 |  | < .001 |  |
|  |  | - |  | AC2.3 | | |  | | | 0.10633 | | |  | 0.0178 | | |  | 36.0 |  | 5.9655 |  | < .001 |  |
|  |  | - |  | CZ1.3 | | |  | | | 0.04333 | | |  | 0.0178 | | |  | 36.0 |  | 2.4311 |  | 0.591 |  |
|  |  | - |  | CZ1.4 | | |  | | | 0.09467 | | |  | 0.0178 | | |  | 36.0 |  | 5.3110 |  | < .001 |  |
|  |  | - |  | OM1.2 | | |  | | | 0.05933 | | |  | 0.0178 | | |  | 36.0 |  | 3.3287 |  | 0.131 |  |
|  |  | - |  | OM1.1 | | |  | | | 0.09300 | | |  | 0.0178 | | |  | 36.0 |  | 5.2175 |  | < .001 |  |
| AC1.2 |  | - |  | AC1.3 | | |  | | | -0.23400 | | |  | 0.0178 | | |  | 36.0 |  | -13.1279 |  | < .001 |  |
|  |  | - |  | AC1.5 | | |  | | | -0.13733 | | |  | 0.0178 | | |  | 36.0 |  | -7.7047 |  | < .001 |  |
|  |  | - |  | OM2.1 | | |  | | | -0.06933 | | |  | 0.0178 | | |  | 36.0 |  | -3.8898 |  | 0.035 |  |
|  |  | - |  | OM2.2 | | |  | | | -0.10967 | | |  | 0.0178 | | |  | 36.0 |  | -6.1525 |  | < .001 |  |
|  |  | - |  | OM2.3 | | |  | | | -0.08167 | | |  | 0.0178 | | |  | 36.0 |  | -4.5817 |  | 0.006 |  |
|  |  | - |  | AC2.1 | | |  | | | 0.00967 | | |  | 0.0178 | | |  | 36.0 |  | 0.5423 |  | 1.000 |  |
|  |  | - |  | AC2.2 | | |  | | | -0.00867 | | |  | 0.0178 | | |  | 36.0 |  | -0.4862 |  | 1.000 |  |
|  |  | - |  | AC2.3 | | |  | | | 0.00200 | | |  | 0.0178 | | |  | 36.0 |  | 0.1122 |  | 1.000 |  |
|  |  | - |  | CZ1.3 | | |  | | | -0.06100 | | |  | 0.0178 | | |  | 36.0 |  | -3.4222 |  | 0.107 |  |
|  |  | - |  | CZ1.4 | | |  | | | -0.00967 | | |  | 0.0178 | | |  | 36.0 |  | -0.5423 |  | 1.000 |  |
|  |  | - |  | OM1.2 | | |  | | | -0.04500 | | |  | 0.0178 | | |  | 36.0 |  | -2.5246 |  | 0.528 |  |
|  |  | - |  | OM1.1 | | |  | | | -0.01133 | | |  | 0.0178 | | |  | 36.0 |  | -0.6358 |  | 1.000 |  |
| AC1.3 |  | - |  | AC1.5 | | |  | | | 0.09667 | | |  | 0.0178 | | |  | 36.0 |  | 5.4232 |  | < .001 |  |
|  |  | - |  | OM2.1 | | |  | | | 0.16467 | | |  | 0.0178 | | |  | 36.0 |  | 9.2382 |  | < .001 |  |
|  |  | - |  | OM2.2 | | |  | | | 0.12433 | | |  | 0.0178 | | |  | 36.0 |  | 6.9754 |  | < .001 |  |
|  |  | - |  | OM2.3 | | |  | | | 0.15233 | | |  | 0.0178 | | |  | 36.0 |  | 8.5462 |  | < .001 |  |
|  |  | - |  | AC2.1 | | |  | | | 0.24367 | | |  | 0.0178 | | |  | 36.0 |  | 13.6703 |  | < .001 |  |
|  |  | - |  | AC2.2 | | |  | | | 0.22533 | | |  | 0.0178 | | |  | 36.0 |  | 12.6417 |  | < .001 |  |
|  |  | - |  | AC2.3 | | |  | | | 0.23600 | | |  | 0.0178 | | |  | 36.0 |  | 13.2401 |  | < .001 |  |
|  |  | - |  | CZ1.3 | | |  | | | 0.17300 | | |  | 0.0178 | | |  | 36.0 |  | 9.7057 |  | < .001 |  |
|  |  | - |  | CZ1.4 | | |  | | | 0.22433 | | |  | 0.0178 | | |  | 36.0 |  | 12.5856 |  | < .001 |  |
|  |  | - |  | OM1.2 | | |  | | | 0.18900 | | |  | 0.0178 | | |  | 36.0 |  | 10.6033 |  | < .001 |  |
|  |  | - |  | OM1.1 | | |  | | | 0.22267 | | |  | 0.0178 | | |  | 36.0 |  | 12.4921 |  | < .001 |  |
| AC1.5 |  | - |  | OM2.1 | | |  | | | 0.06800 | | |  | 0.0178 | | |  | 36.0 |  | 3.8150 |  | 0.043 |  |
|  |  | - |  | OM2.2 | | |  | | | 0.02767 | | |  | 0.0178 | | |  | 36.0 |  | 1.5522 |  | 0.979 |  |
|  |  | - |  | OM2.3 | | |  | | | 0.05567 | | |  | 0.0178 | | |  | 36.0 |  | 3.1230 |  | 0.200 |  |
|  |  | - |  | AC2.1 | | |  | | | 0.14700 | | |  | 0.0178 | | |  | 36.0 |  | 8.2470 |  | < .001 |  |
|  |  | - |  | AC2.2 | | |  | | | 0.12867 | | |  | 0.0178 | | |  | 36.0 |  | 7.2185 |  | < .001 |  |
|  |  | - |  | AC2.3 | | |  | | | 0.13933 | | |  | 0.0178 | | |  | 36.0 |  | 7.8169 |  | < .001 |  |
|  |  | - |  | CZ1.3 | | |  | | | 0.07633 | | |  | 0.0178 | | |  | 36.0 |  | 4.2825 |  | 0.013 |  |
|  |  | - |  | CZ1.4 | | |  | | | 0.12767 | | |  | 0.0178 | | |  | 36.0 |  | 7.1624 |  | < .001 |  |
|  |  | - |  | OM1.2 | | |  | | | 0.09233 | | |  | 0.0178 | | |  | 36.0 |  | 5.1801 |  | 0.001 |  |
|  |  | - |  | OM1.1 | | |  | | | 0.12600 | | |  | 0.0178 | | |  | 36.0 |  | 7.0689 |  | < .001 |  |
| OM2.1 |  | - |  | OM2.2 | | |  | | | -0.04033 | | |  | 0.0178 | | |  | 36.0 |  | -2.2628 |  | 0.702 |  |
|  |  | - |  | OM2.3 | | |  | | | -0.01233 | | |  | 0.0178 | | |  | 36.0 |  | -0.6919 |  | 1.000 |  |
|  |  | - |  | AC2.1 | | |  | | | 0.07900 | | |  | 0.0178 | | |  | 36.0 |  | 4.4321 |  | 0.008 |  |
|  |  | - |  | AC2.2 | | |  | | | 0.06067 | | |  | 0.0178 | | |  | 36.0 |  | 3.4035 |  | 0.112 |  |
|  |  | - |  | AC2.3 | | |  | | | 0.07133 | | |  | 0.0178 | | |  | 36.0 |  | 4.0020 |  | 0.027 |  |
|  |  | - |  | CZ1.3 | | |  | | | 0.00833 | | |  | 0.0178 | | |  | 36.0 |  | 0.4675 |  | 1.000 |  |
|  |  | - |  | CZ1.4 | | |  | | | 0.05967 | | |  | 0.0178 | | |  | 36.0 |  | 3.3474 |  | 0.126 |  |
|  |  | - |  | OM1.2 | | |  | | | 0.02433 | | |  | 0.0178 | | |  | 36.0 |  | 1.3652 |  | 0.994 |  |
|  |  | - |  | OM1.1 | | |  | | | 0.05800 | | |  | 0.0178 | | |  | 36.0 |  | 3.2539 |  | 0.154 |  |
| OM2.2 |  | - |  | OM2.3 | | |  | | | 0.02800 | | |  | 0.0178 | | |  | 36.0 |  | 1.5709 |  | 0.977 |  |
|  |  | - |  | AC2.1 | | |  | | | 0.11933 | | |  | 0.0178 | | |  | 36.0 |  | 6.6949 |  | < .001 |  |
|  |  | - |  | AC2.2 | | |  | | | 0.10100 | | |  | 0.0178 | | |  | 36.0 |  | 5.6663 |  | < .001 |  |
|  |  | - |  | AC2.3 | | |  | | | 0.11167 | | |  | 0.0178 | | |  | 36.0 |  | 6.2648 |  | < .001 |  |
|  |  | - |  | CZ1.3 | | |  | | | 0.04867 | | |  | 0.0178 | | |  | 36.0 |  | 2.7303 |  | 0.397 |  |
|  |  | - |  | CZ1.4 | | |  | | | 0.10000 | | |  | 0.0178 | | |  | 36.0 |  | 5.6102 |  | < .001 |  |
|  |  | - |  | OM1.2 | | |  | | | 0.06467 | | |  | 0.0178 | | |  | 36.0 |  | 3.6279 |  | 0.067 |  |
|  |  | - |  | OM1.1 | | |  | | | 0.09833 | | |  | 0.0178 | | |  | 36.0 |  | 5.5167 |  | < .001 |  |
| OM2.3 |  | - |  | AC2.1 | | |  | | | 0.09133 | | |  | 0.0178 | | |  | 36.0 |  | 5.1240 |  | 0.001 |  |
|  |  | - |  | AC2.2 | | |  | | | 0.07300 | | |  | 0.0178 | | |  | 36.0 |  | 4.0955 |  | 0.021 |  |
|  |  | - |  | AC2.3 | | |  | | | 0.08367 | | |  | 0.0178 | | |  | 36.0 |  | 4.6939 |  | 0.004 |  |
|  |  | - |  | CZ1.3 | | |  | | | 0.02067 | | |  | 0.0178 | | |  | 36.0 |  | 1.1594 |  | 0.999 |  |
|  |  | - |  | CZ1.4 | | |  | | | 0.07200 | | |  | 0.0178 | | |  | 36.0 |  | 4.0394 |  | 0.024 |  |
|  |  | - |  | OM1.2 | | |  | | | 0.03667 | | |  | 0.0178 | | |  | 36.0 |  | 2.0571 |  | 0.822 |  |
|  |  | - |  | OM1.1 | | |  | | | 0.07033 | | |  | 0.0178 | | |  | 36.0 |  | 3.9459 |  | 0.031 |  |
| AC2.1 |  | - |  | AC2.2 | | |  | | | -0.01833 | | |  | 0.0178 | | |  | 36.0 |  | -1.0285 |  | 1.000 |  |
|  |  | - |  | AC2.3 | | |  | | | -0.00767 | | |  | 0.0178 | | |  | 36.0 |  | -0.4301 |  | 1.000 |  |
|  |  | - |  | CZ1.3 | | |  | | | -0.07067 | | |  | 0.0178 | | |  | 36.0 |  | -3.9646 |  | 0.029 |  |
|  |  | - |  | CZ1.4 | | |  | | | -0.01933 | | |  | 0.0178 | | |  | 36.0 |  | -1.0846 |  | 1.000 |  |
|  |  | - |  | OM1.2 | | |  | | | -0.05467 | | |  | 0.0178 | | |  | 36.0 |  | -3.0669 |  | 0.223 |  |
|  |  | - |  | OM1.1 | | |  | | | -0.02100 | | |  | 0.0178 | | |  | 36.0 |  | -1.1781 |  | 0.999 |  |
| AC2.2 |  | - |  | AC2.3 | | |  | | | 0.01067 | | |  | 0.0178 | | |  | 36.0 |  | 0.5984 |  | 1.000 |  |
|  |  | - |  | CZ1.3 | | |  | | | -0.05233 | | |  | 0.0178 | | |  | 36.0 |  | -2.9360 |  | 0.283 |  |
|  |  | - |  | CZ1.4 | | |  | | | -0.00100 | | |  | 0.0178 | | |  | 36.0 |  | -0.0561 |  | 1.000 |  |
|  |  | - |  | OM1.2 | | |  | | | -0.03633 | | |  | 0.0178 | | |  | 36.0 |  | -2.0384 |  | 0.832 |  |
|  |  | - |  | OM1.1 | | |  | | | -0.00267 | | |  | 0.0178 | | |  | 36.0 |  | -0.1496 |  | 1.000 |  |
| AC2.3 |  | - |  | CZ1.3 | | |  | | | -0.06300 | | |  | 0.0178 | | |  | 36.0 |  | -3.5344 |  | 0.083 |  |
|  |  | - |  | CZ1.4 | | |  | | | -0.01167 | | |  | 0.0178 | | |  | 36.0 |  | -0.6545 |  | 1.000 |  |
|  |  | - |  | OM1.2 | | |  | | | -0.04700 | | |  | 0.0178 | | |  | 36.0 |  | -2.6368 |  | 0.455 |  |
|  |  | - |  | OM1.1 | | |  | | | -0.01333 | | |  | 0.0178 | | |  | 36.0 |  | -0.7480 |  | 1.000 |  |
| CZ1.3 |  | - |  | CZ1.4 | | |  | | | 0.05133 | | |  | 0.0178 | | |  | 36.0 |  | 2.8799 |  | 0.312 |  |
|  |  | - |  | OM1.2 | | |  | | | 0.01600 | | |  | 0.0178 | | |  | 36.0 |  | 0.8976 |  | 1.000 |  |
|  |  | - |  | OM1.1 | | |  | | | 0.04967 | | |  | 0.0178 | | |  | 36.0 |  | 2.7864 |  | 0.363 |  |
| CZ1.4 |  | - |  | OM1.2 | | |  | | | -0.03533 | | |  | 0.0178 | | |  | 36.0 |  | -1.9823 |  | 0.859 |  |
|  |  | - |  | OM1.1 | | |  | | | -0.00167 | | |  | 0.0178 | | |  | 36.0 |  | -0.0935 |  | 1.000 |  |
| OM1.2 |  | - |  | OM1.1 | | |  | | | 0.03367 | | |  | 0.0178 | | |  | 36.0 |  | 1.8888 |  | 0.898 |  |
| Note. Comparisons are based on estimated marginal means | | | | | | | | | | | | | | | | | | | | | | | |
|  | | | | | | | | | | | | | | | | | | | | | | | |
| **FPase** Estimated Marginal Means - Isolate Code | | | | | | | | | | | | | | | |  |  |  |  |  |  |  |  |
|  | | | | | | | | | **95% Confidence Interval** | | | | | | |  |  |  |  |  |  |  |  |
| **Isolate Code** | | **Mean** | | | | **SE** | | | **Lower** | | | **Upper** | | | |  |  |  |  |  |  |  |  |
| AC1.4 |  | 0.221 | | |  | 0.0126 | |  | 0.195 | |  | 0.246 | | |  |  |  |  |  |  |  |  |  |
| AC1.6 |  | 0.148 | | |  | 0.0126 | |  | 0.123 | |  | 0.174 | | |  |  |  |  |  |  |  |  |  |
| CZ1.1 |  | 0.143 | | |  | 0.0126 | |  | 0.118 | |  | 0.169 | | |  |  |  |  |  |  |  |  |  |
| CZ1.2 |  | 0.127 | | |  | 0.0126 | |  | 0.101 | |  | 0.152 | | |  |  |  |  |  |  |  |  |  |
| AC1.1 |  | 0.260 | | |  | 0.0126 | |  | 0.234 | |  | 0.286 | | |  |  |  |  |  |  |  |  |  |
| AC1.2 |  | 0.156 | | |  | 0.0126 | |  | 0.130 | |  | 0.181 | | |  |  |  |  |  |  |  |  |  |
| AC1.3 |  | 0.390 | | |  | 0.0126 | |  | 0.364 | |  | 0.415 | | |  |  |  |  |  |  |  |  |  |
| AC1.5 |  | 0.293 | | |  | 0.0126 | |  | 0.267 | |  | 0.319 | | |  |  |  |  |  |  |  |  |  |
| OM2.1 |  | 0.225 | | |  | 0.0126 | |  | 0.199 | |  | 0.251 | | |  |  |  |  |  |  |  |  |  |
| OM2.2 |  | 0.265 | | |  | 0.0126 | |  | 0.240 | |  | 0.291 | | |  |  |  |  |  |  |  |  |  |
| OM2.3 |  | 0.237 | | |  | 0.0126 | |  | 0.212 | |  | 0.263 | | |  |  |  |  |  |  |  |  |  |
| AC2.1 |  | 0.146 | | |  | 0.0126 | |  | 0.120 | |  | 0.172 | | |  |  |  |  |  |  |  |  |  |
| AC2.2 |  | 0.164 | | |  | 0.0126 | |  | 0.139 | |  | 0.190 | | |  |  |  |  |  |  |  |  |  |
| AC2.3 |  | 0.154 | | |  | 0.0126 | |  | 0.128 | |  | 0.179 | | |  |  |  |  |  |  |  |  |  |
| CZ1.3 |  | 0.217 | | |  | 0.0126 | |  | 0.191 | |  | 0.242 | | |  |  |  |  |  |  |  |  |  |
| CZ1.4 |  | 0.165 | | |  | 0.0126 | |  | 0.140 | |  | 0.191 | | |  |  |  |  |  |  |  |  |  |
| OM1.2 |  | 0.201 | | |  | 0.0126 | |  | 0.175 | |  | 0.226 | | |  |  |  |  |  |  |  |  |  |
| OM1.1 |  | 0.167 | | |  | 0.0126 | |  | 0.141 | |  | 0.193 | | |  |  |  |  |  |  |  |  |  |
|  | | | | | | | | | | | | | | | |  |  |  |  |  |  |  |  |

| **FPase** Post Hoc Comparisons - Environment | | | | | | | | | | | | | | | |
| --- | --- | --- | --- | --- | --- | --- | --- | --- | --- | --- | --- | --- | --- | --- | --- |
| **Comparison** | | | | | |  | | | | | | | | | |
| **Envi** | |  | | **Envi** | | **Mean Difference** | | **SE** | | **df** | | **t** | | **p_tukey_** | |
| TERRE |  | - |  | MAR |  | 0.00864 |  | 0.0199 |  | 52.0 |  | 0.435 |  | 0.666 |  |
| Note. Comparisons are based on estimated marginal means | | | | | | | | | | | | | | | |
|  | | | | | | | | | | | | | | | |

| **FPase** Estimated Marginal Means - Envi | | | | | | | | | |
| --- | --- | --- | --- | --- | --- | --- | --- | --- | --- |
|  | | | | | | **95% Confidence Interval** | | | |
| **Envi** | | **Mean** | | **SE** | | **Lower** | | **Upper** | |
| TERRE |  | 0.207 |  | 0.0115 |  | 0.184 |  | 0.230 |  |
| MAR |  | 0.199 |  | 0.0162 |  | 0.166 |  | 0.231 |  |
|  | | | | | | | | | |

| **Cellulolytic Index** Post Hoc Comparisons - Isolate Code | | | | | | | | | | | | | | | |
| --- | --- | --- | --- | --- | --- | --- | --- | --- | --- | --- | --- | --- | --- | --- | --- |
| **Comparison** | | | | | |  | | | | | | | | | |
| **Isolate Code** | |  | | **Isolate Code** | | **Mean Difference** | | **SE** | | **df** | | **t** | | **p_tukey_** | |
| AC1.4 |  | - |  | AC1.6 |  | -6.80e−16 |  | 0.225 |  | 36.0 |  | -3.03e−15 |  | 1.000 |  |
|  |  | - |  | CZ1.1 |  | -2.4333 |  | 0.225 |  | 36.0 |  | -10.829 |  | < .001 |  |
|  |  | - |  | CZ1.2 |  | -1.4000 |  | 0.225 |  | 36.0 |  | -6.230 |  | < .001 |  |
|  |  | - |  | AC1.1 |  | -0.5000 |  | 0.225 |  | 36.0 |  | -2.225 |  | 0.726 |  |
|  |  | - |  | AC1.2 |  | -0.2000 |  | 0.225 |  | 36.0 |  | -0.890 |  | 1.000 |  |
|  |  | - |  | AC1.3 |  | -0.1000 |  | 0.225 |  | 36.0 |  | -0.445 |  | 1.000 |  |
|  |  | - |  | AC1.5 |  | 0.0667 |  | 0.225 |  | 36.0 |  | 0.297 |  | 1.000 |  |
|  |  | - |  | OM2.1 |  | -0.2333 |  | 0.225 |  | 36.0 |  | -1.038 |  | 1.000 |  |
|  |  | - |  | OM2.2 |  | -0.1000 |  | 0.225 |  | 36.0 |  | -0.445 |  | 1.000 |  |
|  |  | - |  | OM2.3 |  | 0.9000 |  | 0.225 |  | 36.0 |  | 4.005 |  | 0.026 |  |
|  |  | - |  | AC2.1 |  | 0.0667 |  | 0.225 |  | 36.0 |  | 0.297 |  | 1.000 |  |
|  |  | - |  | AC2.2 |  | 1.0333 |  | 0.225 |  | 36.0 |  | 4.599 |  | 0.005 |  |
|  |  | - |  | AC2.3 |  | 1.2333 |  | 0.225 |  | 36.0 |  | 5.489 |  | < .001 |  |
|  |  | - |  | CZ1.3 |  | 0.1667 |  | 0.225 |  | 36.0 |  | 0.742 |  | 1.000 |  |
|  |  | - |  | CZ1.4 |  | 0.2000 |  | 0.225 |  | 36.0 |  | 0.890 |  | 1.000 |  |
|  |  | - |  | OM1.2 |  | 1.61e-15 |  | 0.225 |  | 36.0 |  | 7.16e-15 |  | 1.000 |  |
|  |  | - |  | OM1.1 |  | -0.8667 |  | 0.225 |  | 36.0 |  | -3.857 |  | 0.038 |  |
| AC1.6 |  | - |  | CZ1.1 |  | -2.4333 |  | 0.225 |  | 36.0 |  | -10.829 |  | < .001 |  |
|  |  | - |  | CZ1.2 |  | -1.4000 |  | 0.225 |  | 36.0 |  | -6.230 |  | < .001 |  |
|  |  | - |  | AC1.1 |  | -0.5000 |  | 0.225 |  | 36.0 |  | -2.225 |  | 0.726 |  |
|  |  | - |  | AC1.2 |  | -0.2000 |  | 0.225 |  | 36.0 |  | -0.890 |  | 1.000 |  |
|  |  | - |  | AC1.3 |  | -0.1000 |  | 0.225 |  | 36.0 |  | -0.445 |  | 1.000 |  |
|  |  | - |  | AC1.5 |  | 0.0667 |  | 0.225 |  | 36.0 |  | 0.297 |  | 1.000 |  |
|  |  | - |  | OM2.1 |  | -0.2333 |  | 0.225 |  | 36.0 |  | -1.038 |  | 1.000 |  |
|  |  | - |  | OM2.2 |  | -0.1000 |  | 0.225 |  | 36.0 |  | -0.445 |  | 1.000 |  |
|  |  | - |  | OM2.3 |  | 0.9000 |  | 0.225 |  | 36.0 |  | 4.005 |  | 0.026 |  |
|  |  | - |  | AC2.1 |  | 0.0667 |  | 0.225 |  | 36.0 |  | 0.297 |  | 1.000 |  |
|  |  | - |  | AC2.2 |  | 1.0333 |  | 0.225 |  | 36.0 |  | 4.599 |  | 0.005 |  |
|  |  | - |  | AC2.3 |  | 1.2333 |  | 0.225 |  | 36.0 |  | 5.489 |  | < .001 |  |
|  |  | - |  | CZ1.3 |  | 0.1667 |  | 0.225 |  | 36.0 |  | 0.742 |  | 1.000 |  |
|  |  | - |  | CZ1.4 |  | 0.2000 |  | 0.225 |  | 36.0 |  | 0.890 |  | 1.000 |  |
|  |  | - |  | OM1.2 |  | 2.29e-15 |  | 0.225 |  | 36.0 |  | 1.02e-14 |  | 1.000 |  |
|  |  | - |  | OM1.1 |  | -0.8667 |  | 0.225 |  | 36.0 |  | -3.857 |  | 0.038 |  |
| CZ1.1 |  | - |  | CZ1.2 |  | 1.0333 |  | 0.225 |  | 36.0 |  | 4.599 |  | 0.005 |  |
|  |  | - |  | AC1.1 |  | 1.9333 |  | 0.225 |  | 36.0 |  | 8.604 |  | < .001 |  |
|  |  | - |  | AC1.2 |  | 2.2333 |  | 0.225 |  | 36.0 |  | 9.939 |  | < .001 |  |
|  |  | - |  | AC1.3 |  | 2.3333 |  | 0.225 |  | 36.0 |  | 10.384 |  | < .001 |  |
|  |  | - |  | AC1.5 |  | 2.5000 |  | 0.225 |  | 36.0 |  | 11.126 |  | < .001 |  |
|  |  | - |  | OM2.1 |  | 2.2000 |  | 0.225 |  | 36.0 |  | 9.790 |  | < .001 |  |
|  |  | - |  | OM2.2 |  | 2.3333 |  | 0.225 |  | 36.0 |  | 10.384 |  | < .001 |  |
|  |  | - |  | OM2.3 |  | 3.3333 |  | 0.225 |  | 36.0 |  | 14.834 |  | < .001 |  |
|  |  | - |  | AC2.1 |  | 2.5000 |  | 0.225 |  | 36.0 |  | 11.126 |  | < .001 |  |
|  |  | - |  | AC2.2 |  | 3.4667 |  | 0.225 |  | 36.0 |  | 15.427 |  | < .001 |  |
|  |  | - |  | AC2.3 |  | 3.6667 |  | 0.225 |  | 36.0 |  | 16.317 |  | < .001 |  |
|  |  | - |  | CZ1.3 |  | 2.6000 |  | 0.225 |  | 36.0 |  | 11.571 |  | < .001 |  |
|  |  | - |  | CZ1.4 |  | 2.6333 |  | 0.225 |  | 36.0 |  | 11.719 |  | < .001 |  |
|  |  | - |  | OM1.2 |  | 2.4333 |  | 0.225 |  | 36.0 |  | 10.829 |  | < .001 |  |
|  |  | - |  | OM1.1 |  | 1.5667 |  | 0.225 |  | 36.0 |  | 6.972 |  | < .001 |  |
| CZ1.2 |  | - |  | AC1.1 |  | 0.9000 |  | 0.225 |  | 36.0 |  | 4.005 |  | 0.026 |  |
|  |  | - |  | AC1.2 |  | 1.2000 |  | 0.225 |  | 36.0 |  | 5.340 |  | < .001 |  |
|  |  | - |  | AC1.3 |  | 1.3000 |  | 0.225 |  | 36.0 |  | 5.785 |  | < .001 |  |
|  |  | - |  | AC1.5 |  | 1.4667 |  | 0.225 |  | 36.0 |  | 6.527 |  | < .001 |  |
|  |  | - |  | OM2.1 |  | 1.1667 |  | 0.225 |  | 36.0 |  | 5.192 |  | < .001 |  |
|  |  | - |  | OM2.2 |  | 1.3000 |  | 0.225 |  | 36.0 |  | 5.785 |  | < .001 |  |
|  |  | - |  | OM2.3 |  | 2.3000 |  | 0.225 |  | 36.0 |  | 10.235 |  | < .001 |  |
|  |  | - |  | AC2.1 |  | 1.4667 |  | 0.225 |  | 36.0 |  | 6.527 |  | < .001 |  |
|  |  | - |  | AC2.2 |  | 2.4333 |  | 0.225 |  | 36.0 |  | 10.829 |  | < .001 |  |
|  |  | - |  | AC2.3 |  | 2.6333 |  | 0.225 |  | 36.0 |  | 11.719 |  | < .001 |  |
|  |  | - |  | CZ1.3 |  | 1.5667 |  | 0.225 |  | 36.0 |  | 6.972 |  | < .001 |  |
|  |  | - |  | CZ1.4 |  | 1.6000 |  | 0.225 |  | 36.0 |  | 7.120 |  | < .001 |  |
|  |  | - |  | OM1.2 |  | 1.4000 |  | 0.225 |  | 36.0 |  | 6.230 |  | < .001 |  |
|  |  | - |  | OM1.1 |  | 0.5333 |  | 0.225 |  | 36.0 |  | 2.373 |  | 0.630 |  |
| AC1.1 |  | - |  | AC1.2 |  | 0.3000 |  | 0.225 |  | 36.0 |  | 1.335 |  | 0.995 |  |
|  |  | - |  | AC1.3 |  | 0.4000 |  | 0.225 |  | 36.0 |  | 1.780 |  | 0.934 |  |
|  |  | - |  | AC1.5 |  | 0.5667 |  | 0.225 |  | 36.0 |  | 2.522 |  | 0.530 |  |
|  |  | - |  | OM2.1 |  | 0.2667 |  | 0.225 |  | 36.0 |  | 1.187 |  | 0.999 |  |
|  |  | - |  | OM2.2 |  | 0.4000 |  | 0.225 |  | 36.0 |  | 1.780 |  | 0.934 |  |
|  |  | - |  | OM2.3 |  | 1.4000 |  | 0.225 |  | 36.0 |  | 6.230 |  | < .001 |  |
|  |  | - |  | AC2.1 |  | 0.5667 |  | 0.225 |  | 36.0 |  | 2.522 |  | 0.530 |  |
|  |  | - |  | AC2.2 |  | 1.5333 |  | 0.225 |  | 36.0 |  | 6.824 |  | < .001 |  |
|  |  | - |  | AC2.3 |  | 1.7333 |  | 0.225 |  | 36.0 |  | 7.714 |  | < .001 |  |
|  |  | - |  | CZ1.3 |  | 0.6667 |  | 0.225 |  | 36.0 |  | 2.967 |  | 0.268 |  |
|  |  | - |  | CZ1.4 |  | 0.7000 |  | 0.225 |  | 36.0 |  | 3.115 |  | 0.203 |  |
|  |  | - |  | OM1.2 |  | 0.5000 |  | 0.225 |  | 36.0 |  | 2.225 |  | 0.726 |  |
|  |  | - |  | OM1.1 |  | -0.3667 |  | 0.225 |  | 36.0 |  | -1.632 |  | 0.968 |  |
| AC1.2 |  | - |  | AC1.3 |  | 0.1000 |  | 0.225 |  | 36.0 |  | 0.445 |  | 1.000 |  |
|  |  | - |  | AC1.5 |  | 0.2667 |  | 0.225 |  | 36.0 |  | 1.187 |  | 0.999 |  |
|  |  | - |  | OM2.1 |  | -0.0333 |  | 0.225 |  | 36.0 |  | -0.148 |  | 1.000 |  |
|  |  | - |  | OM2.2 |  | 0.1000 |  | 0.225 |  | 36.0 |  | 0.445 |  | 1.000 |  |
|  |  | - |  | OM2.3 |  | 1.1000 |  | 0.225 |  | 36.0 |  | 4.895 |  | 0.002 |  |
|  |  | - |  | AC2.1 |  | 0.2667 |  | 0.225 |  | 36.0 |  | 1.187 |  | 0.999 |  |
|  |  | - |  | AC2.2 |  | 1.2333 |  | 0.225 |  | 36.0 |  | 5.489 |  | < .001 |  |
|  |  | - |  | AC2.3 |  | 1.4333 |  | 0.225 |  | 36.0 |  | 6.379 |  | < .001 |  |
|  |  | - |  | CZ1.3 |  | 0.3667 |  | 0.225 |  | 36.0 |  | 1.632 |  | 0.968 |  |
|  |  | - |  | CZ1.4 |  | 0.4000 |  | 0.225 |  | 36.0 |  | 1.780 |  | 0.934 |  |
|  |  | - |  | OM1.2 |  | 0.2000 |  | 0.225 |  | 36.0 |  | 0.890 |  | 1.000 |  |
|  |  | - |  | OM1.1 |  | -0.6667 |  | 0.225 |  | 36.0 |  | -2.967 |  | 0.268 |  |
| AC1.3 |  | - |  | AC1.5 |  | 0.1667 |  | 0.225 |  | 36.0 |  | 0.742 |  | 1.000 |  |
|  |  | - |  | OM2.1 |  | -0.1333 |  | 0.225 |  | 36.0 |  | -0.593 |  | 1.000 |  |
|  |  | - |  | OM2.2 |  | 1.53e-16 |  | 0.225 |  | 36.0 |  | 6.79e-16 |  | 1.000 |  |
|  |  | - |  | OM2.3 |  | 1.0000 |  | 0.225 |  | 36.0 |  | 4.450 |  | 0.008 |  |
|  |  | - |  | AC2.1 |  | 0.1667 |  | 0.225 |  | 36.0 |  | 0.742 |  | 1.000 |  |
|  |  | - |  | AC2.2 |  | 1.1333 |  | 0.225 |  | 36.0 |  | 5.044 |  | 0.001 |  |
|  |  | - |  | AC2.3 |  | 1.3333 |  | 0.225 |  | 36.0 |  | 5.934 |  | < .001 |  |
|  |  | - |  | CZ1.3 |  | 0.2667 |  | 0.225 |  | 36.0 |  | 1.187 |  | 0.999 |  |
|  |  | - |  | CZ1.4 |  | 0.3000 |  | 0.225 |  | 36.0 |  | 1.335 |  | 0.995 |  |
|  |  | - |  | OM1.2 |  | 0.1000 |  | 0.225 |  | 36.0 |  | 0.445 |  | 1.000 |  |
|  |  | - |  | OM1.1 |  | -0.7667 |  | 0.225 |  | 36.0 |  | -3.412 |  | 0.110 |  |
| AC1.5 |  | - |  | OM2.1 |  | -0.3000 |  | 0.225 |  | 36.0 |  | -1.335 |  | 0.995 |  |
|  |  | - |  | OM2.2 |  | -0.1667 |  | 0.225 |  | 36.0 |  | -0.742 |  | 1.000 |  |
|  |  | - |  | OM2.3 |  | 0.8333 |  | 0.225 |  | 36.0 |  | 3.709 |  | 0.055 |  |
|  |  | - |  | AC2.1 |  | 1.11e-16 |  | 0.225 |  | 36.0 |  | 4.94e-16 |  | 1.000 |  |
|  |  | - |  | AC2.2 |  | 0.9667 |  | 0.225 |  | 36.0 |  | 4.302 |  | 0.012 |  |
|  |  | - |  | AC2.3 |  | 1.1667 |  | 0.225 |  | 36.0 |  | 5.192 |  | < .001 |  |
|  |  | - |  | CZ1.3 |  | 0.1000 |  | 0.225 |  | 36.0 |  | 0.445 |  | 1.000 |  |
|  |  | - |  | CZ1.4 |  | 0.1333 |  | 0.225 |  | 36.0 |  | 0.593 |  | 1.000 |  |
|  |  | - |  | OM1.2 |  | -0.0667 |  | 0.225 |  | 36.0 |  | -0.297 |  | 1.000 |  |
|  |  | - |  | OM1.1 |  | -0.9333 |  | 0.225 |  | 36.0 |  | -4.154 |  | 0.018 |  |
| OM2.1 |  | - |  | OM2.2 |  | 0.1333 |  | 0.225 |  | 36.0 |  | 0.593 |  | 1.000 |  |
|  |  | - |  | OM2.3 |  | 1.1333 |  | 0.225 |  | 36.0 |  | 5.044 |  | 0.001 |  |
|  |  | - |  | AC2.1 |  | 0.3000 |  | 0.225 |  | 36.0 |  | 1.335 |  | 0.995 |  |
|  |  | - |  | AC2.2 |  | 1.2667 |  | 0.225 |  | 36.0 |  | 5.637 |  | < .001 |  |
|  |  | - |  | AC2.3 |  | 1.4667 |  | 0.225 |  | 36.0 |  | 6.527 |  | < .001 |  |
|  |  | - |  | CZ1.3 |  | 0.4000 |  | 0.225 |  | 36.0 |  | 1.780 |  | 0.934 |  |
|  |  | - |  | CZ1.4 |  | 0.4333 |  | 0.225 |  | 36.0 |  | 1.928 |  | 0.882 |  |
|  |  | - |  | OM1.2 |  | 0.2333 |  | 0.225 |  | 36.0 |  | 1.038 |  | 1.000 |  |
|  |  | - |  | OM1.1 |  | -0.6333 |  | 0.225 |  | 36.0 |  | -2.818 |  | 0.345 |  |
| OM2.2 |  | - |  | OM2.3 |  | 1.0000 |  | 0.225 |  | 36.0 |  | 4.450 |  | 0.008 |  |
|  |  | - |  | AC2.1 |  | 0.1667 |  | 0.225 |  | 36.0 |  | 0.742 |  | 1.000 |  |
|  |  | - |  | AC2.2 |  | 1.1333 |  | 0.225 |  | 36.0 |  | 5.044 |  | 0.001 |  |
|  |  | - |  | AC2.3 |  | 1.3333 |  | 0.225 |  | 36.0 |  | 5.934 |  | < .001 |  |
|  |  | - |  | CZ1.3 |  | 0.2667 |  | 0.225 |  | 36.0 |  | 1.187 |  | 0.999 |  |
|  |  | - |  | CZ1.4 |  | 0.3000 |  | 0.225 |  | 36.0 |  | 1.335 |  | 0.995 |  |
|  |  | - |  | OM1.2 |  | 0.1000 |  | 0.225 |  | 36.0 |  | 0.445 |  | 1.000 |  |
|  |  | - |  | OM1.1 |  | -0.7667 |  | 0.225 |  | 36.0 |  | -3.412 |  | 0.110 |  |
| OM2.3 |  | - |  | AC2.1 |  | -0.8333 |  | 0.225 |  | 36.0 |  | -3.709 |  | 0.055 |  |
|  |  | - |  | AC2.2 |  | 0.1333 |  | 0.225 |  | 36.0 |  | 0.593 |  | 1.000 |  |
|  |  | - |  | AC2.3 |  | 0.3333 |  | 0.225 |  | 36.0 |  | 1.483 |  | 0.987 |  |
|  |  | - |  | CZ1.3 |  | -0.7333 |  | 0.225 |  | 36.0 |  | -3.263 |  | 0.151 |  |
|  |  | - |  | CZ1.4 |  | -0.7000 |  | 0.225 |  | 36.0 |  | -3.115 |  | 0.203 |  |
|  |  | - |  | OM1.2 |  | -0.9000 |  | 0.225 |  | 36.0 |  | -4.005 |  | 0.026 |  |
|  |  | - |  | OM1.1 |  | -1.7667 |  | 0.225 |  | 36.0 |  | -7.862 |  | < .001 |  |
| AC2.1 |  | - |  | AC2.2 |  | 0.9667 |  | 0.225 |  | 36.0 |  | 4.302 |  | 0.012 |  |
|  |  | - |  | AC2.3 |  | 1.1667 |  | 0.225 |  | 36.0 |  | 5.192 |  | < .001 |  |
|  |  | - |  | CZ1.3 |  | 0.1000 |  | 0.225 |  | 36.0 |  | 0.445 |  | 1.000 |  |
|  |  | - |  | CZ1.4 |  | 0.1333 |  | 0.225 |  | 36.0 |  | 0.593 |  | 1.000 |  |
|  |  | - |  | OM1.2 |  | -0.0667 |  | 0.225 |  | 36.0 |  | -0.297 |  | 1.000 |  |
|  |  | - |  | OM1.1 |  | -0.9333 |  | 0.225 |  | 36.0 |  | -4.154 |  | 0.018 |  |
| AC2.2 |  | - |  | AC2.3 |  | 0.2000 |  | 0.225 |  | 36.0 |  | 0.890 |  | 1.000 |  |
|  |  | - |  | CZ1.3 |  | -0.8667 |  | 0.225 |  | 36.0 |  | -3.857 |  | 0.038 |  |
|  |  | - |  | CZ1.4 |  | -0.8333 |  | 0.225 |  | 36.0 |  | -3.709 |  | 0.055 |  |
|  |  | - |  | OM1.2 |  | -1.0333 |  | 0.225 |  | 36.0 |  | -4.599 |  | 0.005 |  |
|  |  | - |  | OM1.1 |  | -1.9000 |  | 0.225 |  | 36.0 |  | -8.455 |  | < .001 |  |
| AC2.3 |  | - |  | CZ1.3 |  | -1.0667 |  | 0.225 |  | 36.0 |  | -4.747 |  | 0.004 |  |
|  |  | - |  | CZ1.4 |  | -1.0333 |  | 0.225 |  | 36.0 |  | -4.599 |  | 0.005 |  |
|  |  | - |  | OM1.2 |  | -1.2333 |  | 0.225 |  | 36.0 |  | -5.489 |  | < .001 |  |
|  |  | - |  | OM1.1 |  | -2.1000 |  | 0.225 |  | 36.0 |  | -9.345 |  | < .001 |  |
| CZ1.3 |  | - |  | CZ1.4 |  | 0.0333 |  | 0.225 |  | 36.0 |  | 0.148 |  | 1.000 |  |
|  |  | - |  | OM1.2 |  | -0.1667 |  | 0.225 |  | 36.0 |  | -0.742 |  | 1.000 |  |
|  |  | - |  | OM1.1 |  | -1.0333 |  | 0.225 |  | 36.0 |  | -4.599 |  | 0.005 |  |
| CZ1.4 |  | - |  | OM1.2 |  | -0.2000 |  | 0.225 |  | 36.0 |  | -0.890 |  | 1.000 |  |
|  |  | - |  | OM1.1 |  | -1.0667 |  | 0.225 |  | 36.0 |  | -4.747 |  | 0.004 |  |
| OM1.2 |  | - |  | OM1.1 |  | -0.8667 |  | 0.225 |  | 36.0 |  | -3.857 |  | 0.038 |  |
| Note. Comparisons are based on estimated marginal means | | | | | | | | | | | | | | | |
|  | | | | | | | | | | | | | | | |

| **Cellulolytic Index** Estimated Marginal Means - Isolate Code | | | | | | | | | |
| --- | --- | --- | --- | --- | --- | --- | --- | --- | --- |
|  | | | | | | **95% Confidence Interval** | | | |
| **Isolate Code** | | **Mean** | | **SE** | | **Lower** | | **Upper** | |
| AC1.4 |  | 4.27 |  | 0.159 |  | 3.94 |  | 4.59 |  |
| AC1.6 |  | 4.27 |  | 0.159 |  | 3.94 |  | 4.59 |  |
| CZ1.1 |  | 6.70 |  | 0.159 |  | 6.38 |  | 7.02 |  |
| CZ1.2 |  | 5.67 |  | 0.159 |  | 5.34 |  | 5.99 |  |
| AC1.1 |  | 4.77 |  | 0.159 |  | 4.44 |  | 5.09 |  |
| AC1.2 |  | 4.47 |  | 0.159 |  | 4.14 |  | 4.79 |  |
| AC1.3 |  | 4.37 |  | 0.159 |  | 4.04 |  | 4.69 |  |
| AC1.5 |  | 4.20 |  | 0.159 |  | 3.88 |  | 4.52 |  |
| OM2.1 |  | 4.50 |  | 0.159 |  | 4.18 |  | 4.82 |  |
| OM2.2 |  | 4.37 |  | 0.159 |  | 4.04 |  | 4.69 |  |
| OM2.3 |  | 3.37 |  | 0.159 |  | 3.04 |  | 3.69 |  |
| AC2.1 |  | 4.20 |  | 0.159 |  | 3.88 |  | 4.52 |  |
| AC2.2 |  | 3.23 |  | 0.159 |  | 2.91 |  | 3.56 |  |
| AC2.3 |  | 3.03 |  | 0.159 |  | 2.71 |  | 3.36 |  |
| CZ1.3 |  | 4.10 |  | 0.159 |  | 3.78 |  | 4.42 |  |
| CZ1.4 |  | 4.07 |  | 0.159 |  | 3.74 |  | 4.39 |  |
| OM1.2 |  | 4.27 |  | 0.159 |  | 3.94 |  | 4.59 |  |
| OM1.1 |  | 5.13 |  | 0.159 |  | 4.81 |  | 5.46 |  |
|  | | | | | | | | | |

| **Cellulolytic Index** Post Hoc Comparisons - Environment | | | | | | | | | | | | | | | |
| --- | --- | --- | --- | --- | --- | --- | --- | --- | --- | --- | --- | --- | --- | --- | --- |
| **Comparison** | | | | | |  | | | | | | | | | |
| **Envi** | |  | | **Envi** | | **Mean Difference** | | **SE** | | **df** | | **t** | | **p_tukey_** | |
| TERRE |  | - |  | MAR |  | 0.906 |  | 0.218 |  | 52.0 |  | 4.16 |  | < .001 |  |
| Note. Comparisons are based on estimated marginal means | | | | | | | | | | | | | | | |
|  | | | | | | | | | | | | | | | |

| **Cellulolytic Index** Estimated Marginal Means - Envi | | | | | | | | | |
| --- | --- | --- | --- | --- | --- | --- | --- | --- | --- |
|  | | | | | | **95% Confidence Interval** | | | |
| **Envi** | | **Mean** | | **SE** | | **Lower** | | **Upper** | |
| TERRE |  | 4.69 |  | 0.126 |  | 4.44 |  | 4.94 |  |
| MAR |  | 3.78 |  | 0.178 |  | 3.43 |  | 4.14 |  |
|  | | | | | | | | | |

**References:**

Minitab, LLC. (2021). *Minitab*. Retrieved from https://www.minitab.com

The jamovi project (2023). jamovi (Version 2.3) [Computer Software]. Retrieved from https://www.jamovi.org
