# Supplementary material for: Diversity and Screening of Cellulolytic Microorganisms from Mangrove Forests, Natural Parks, Paddy Field, and Sugarcane Plantation in Panay Island, Philippines
Source: Int J Microbiol. 2024 Jul 23;2024:5573158. doi: 10.1155/2024/5573158 (PMC11288694; doi:10.1155/2024/5573158)
Supplement: Supplementary Materials — Supplementary 1. Fig. S1: Example of cellulolytic microorganisms isolated from the collected soils within Panay Island. A: actinomycete; B: bacteria; C–D: fungi; E–F: pigmented isolates. Supplementary 2. Fig. S2: Example of cellulolytic microorganisms growing on agar plates with rice husk (A) or cellulose (B) as the sole carbon source. Supplementary 3. Fig. S3: Example of cellulolytic microorganisms showing clear zones on CMC agar plates following the addition of 1% Gram iodine solution. Supplementary 4. Fig. S4: The maximum likelihood phylogenetic tree of the genus Enterobacter based on the K2 + G + I model of DNA substitution; bootstrap values less than 50% are not shown. The tree is rooted on Escherichia coli. Red font represents the isolate(s) in this study. Supplementary 5. Fig. S5: The neighbor-joining phylogenetic tree of the genus Rhodococcus based on the TN93 + G model of DNA substitution; bootstrap values less than 50% are not shown. The tree is rooted on a member of Corynebacterium. Red font represents the isolate(s) in this study. Supplementary 6. Fig. S6: The maximum likelihood phylogenetic tree of the genus Streptomyces based on the T92 + G model of DNA substitution; bootstrap values less than 50% are not shown. The tree is rooted on a member of Kitasatospora. Red font represents the isolate(s) in this study. Supplementary 7. Fig. S7: The neighbor-joining phylogenetic tree of the genus Brevibacillus based on the T92 + G model of DNA substitution; bootstrap values less than 50% are not shown. The tree is rooted on members of Bacillus and Paenibacillus. Red font represents the isolate(s) in this study. Supplementary 8. Fig. S8: The maximum likelihood phylogenetic tree of the genus Cellulomonas based on the K2 + G model of DNA substitution; bootstrap values less than 50% are not shown. The tree is rooted on a member of Actinotalea. Red font represents the isolate(s) in this study. Supplementary 9. Fig. S9: The neighbor-joining phylogenetic tree of the gen [file 5573158.f1.zip › Supplementary materials_FINAL.docx]

**SUPPLEMENTARY MATERIALS**


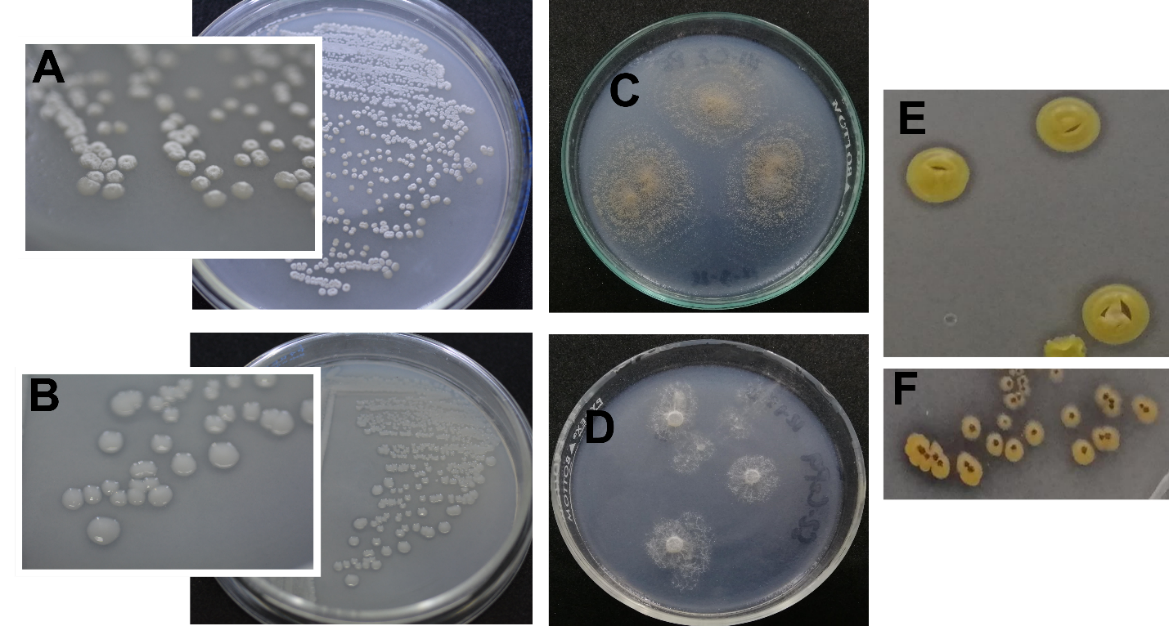


**Fig. S1.** Example of cellulolytic microorganisms isolated from the collected soils within Panay Island. A: actinomycete; B: bacteria; C-D: fungi; E-F: pigmented isolates.

**
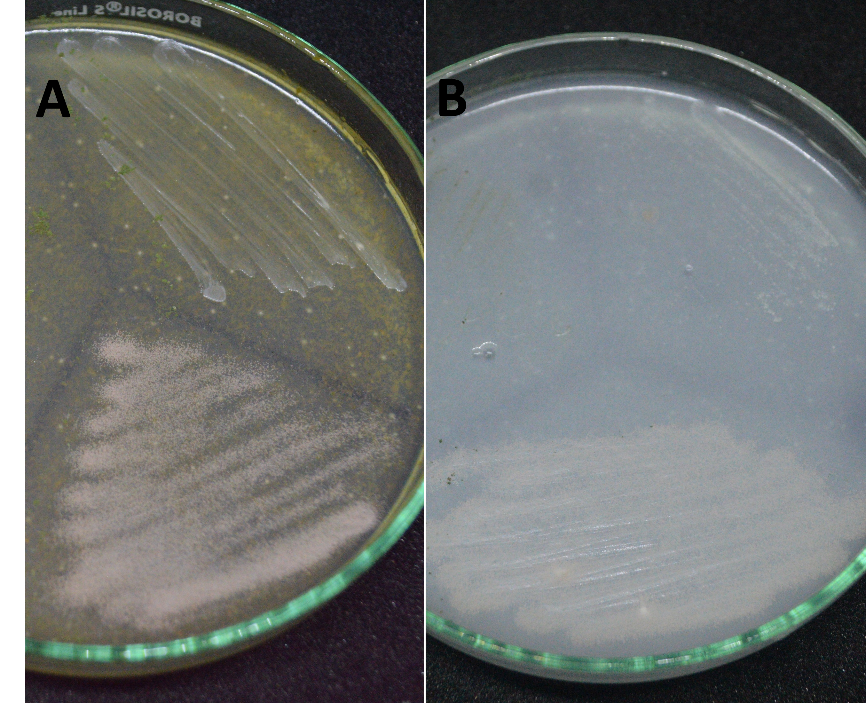
**

**Fig. S2.** Example of cellulolytic microorganisms growing on agar plates with rice husk (A) or cellulose (B) as the sole carbon source.


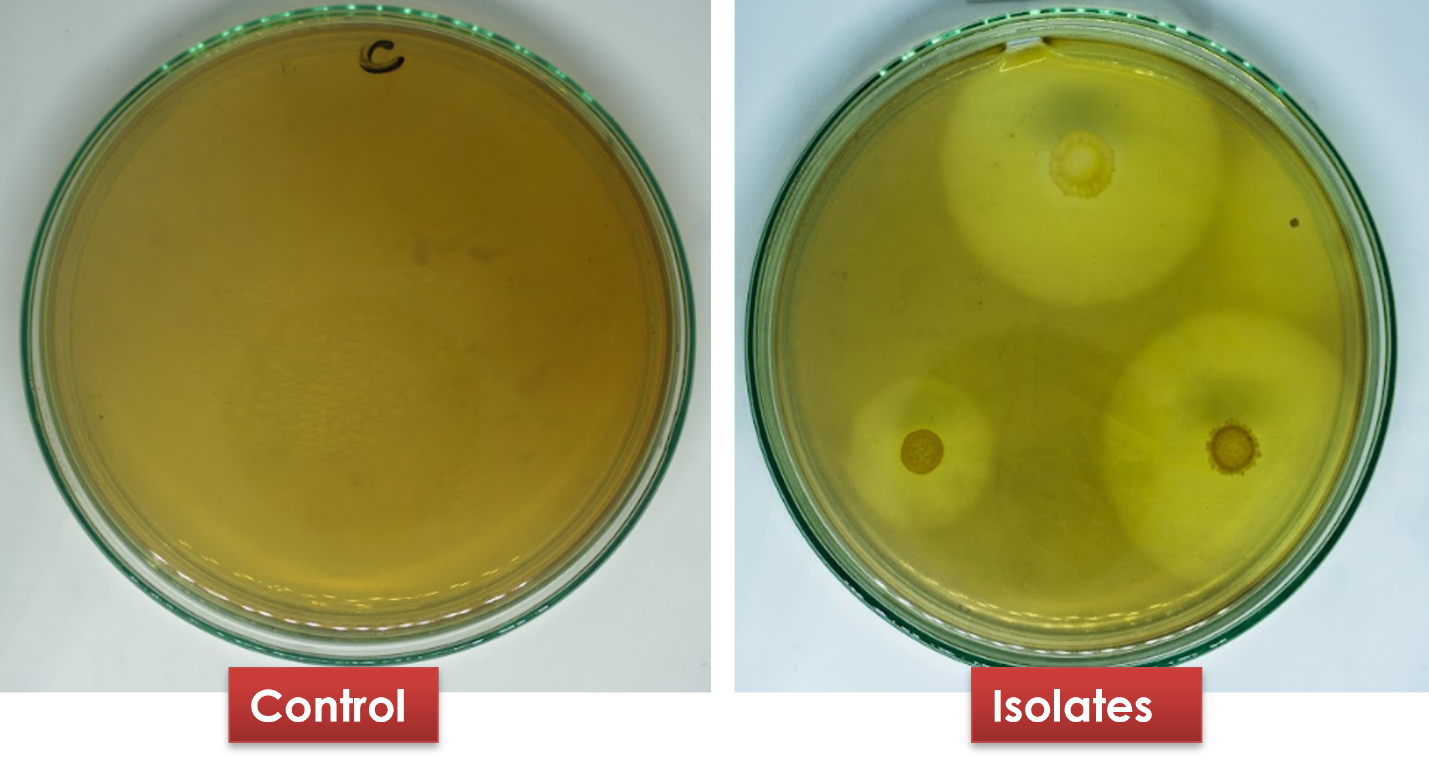


**Fig. S3.** Example of cellulolytic microorganisms showing clear zones on CMC agar plates following the addition of 1% Gram iodine solution.


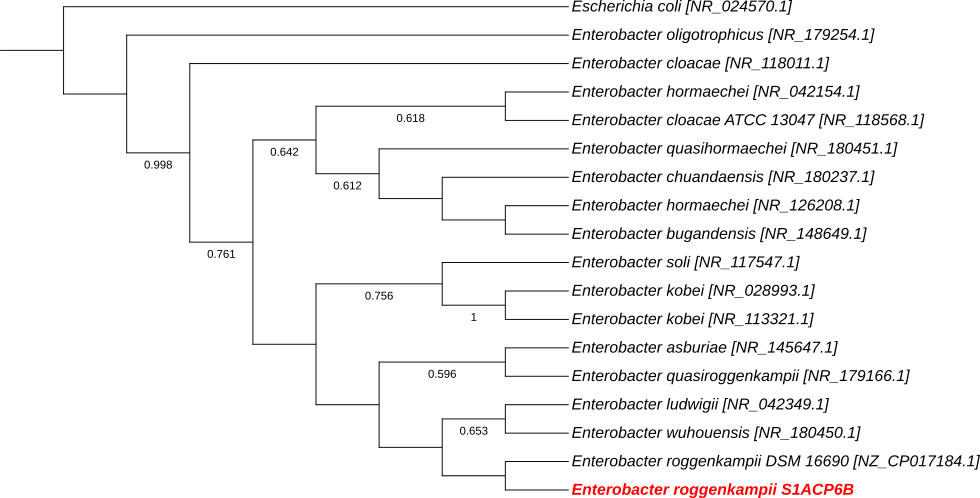


**Fig. S4.** The maximum likelihood phylogenetic tree of the genus *Enterobacter* based on the K2 + G + I model of DNA substitution; bootstrap values less than 50% are not shown. The tree is rooted on *Escherichia coli*. Red font represents the isolate(s) in this study.


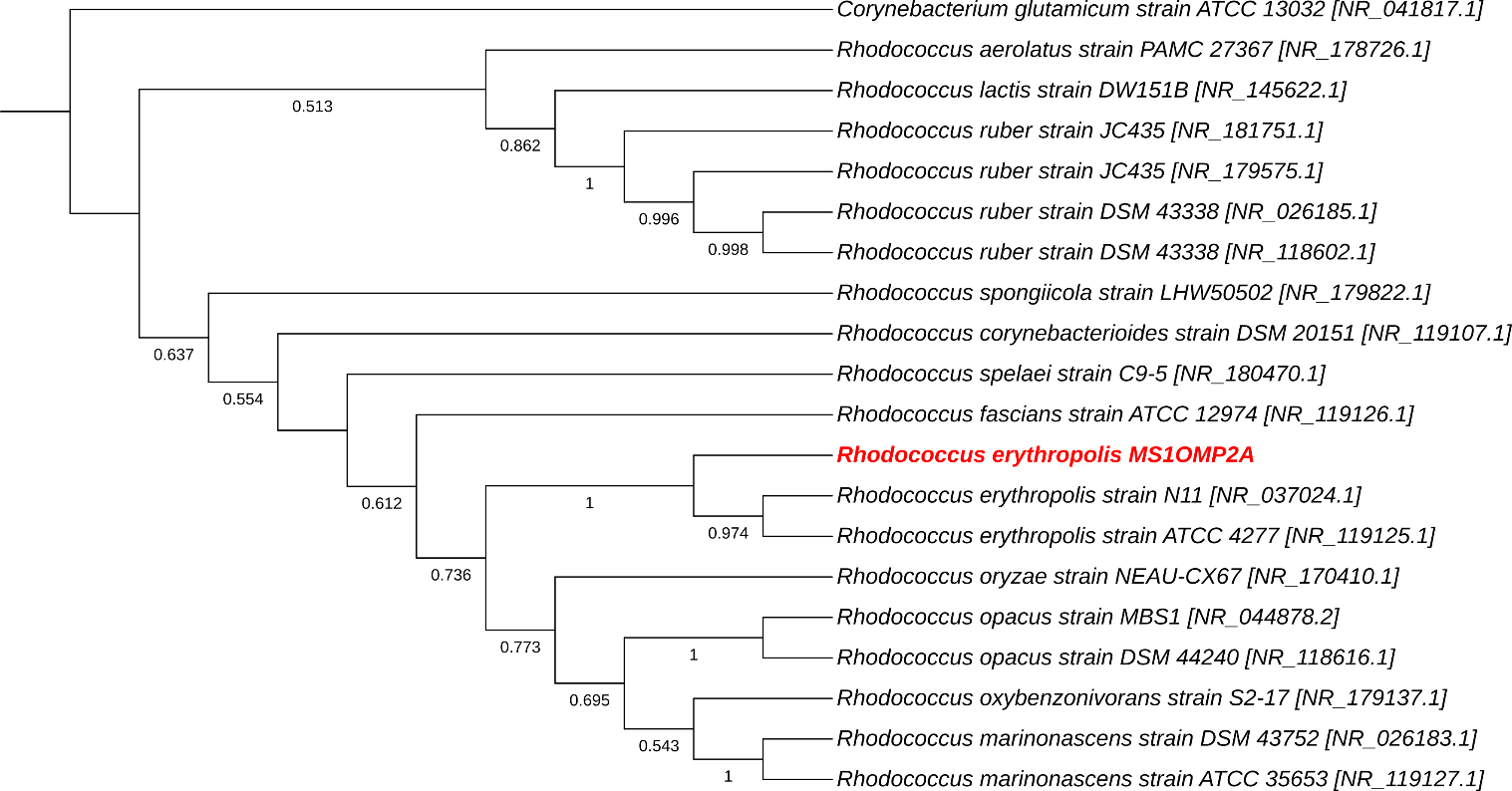


**Fig. S5.** The neighbor-joining phylogenetic tree of the genus *Rhodococcus* based on the TN93 + G model of DNA substitution; bootstrap values less than 50% are not shown. The tree is rooted on a member of *Corynebacterium*. Red font represents the isolate(s) in this study.


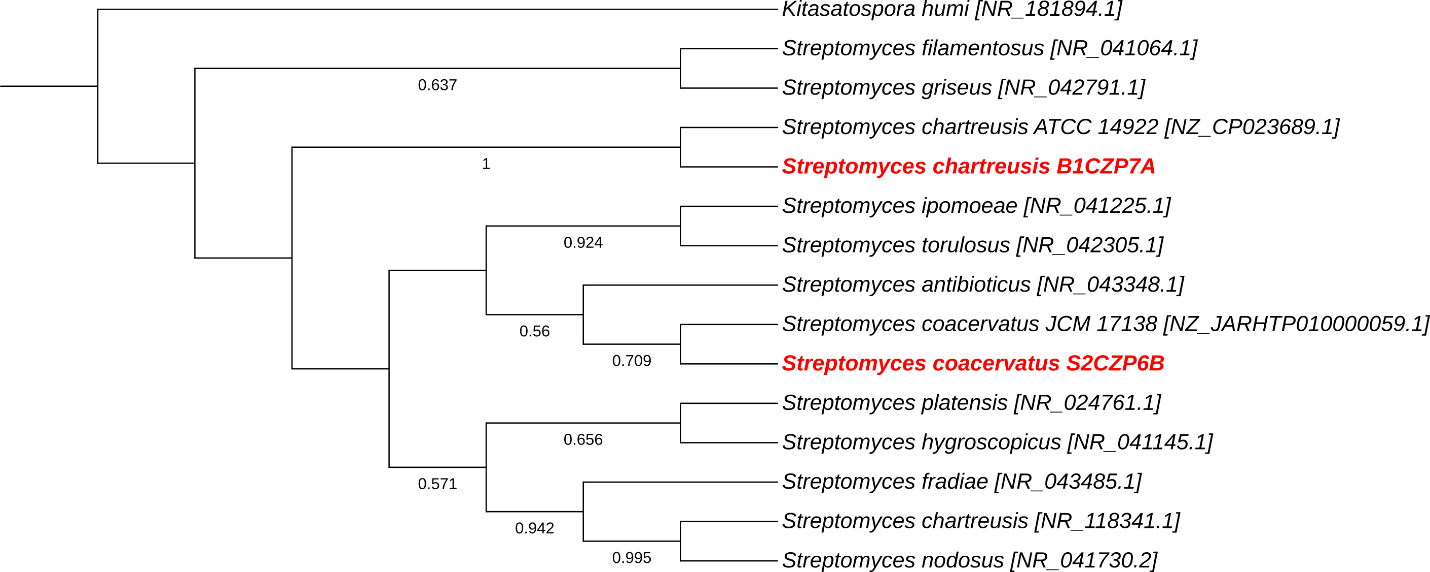


**Fig. S6.** The maximum likelihood phylogenetic tree of the genus *Streptomyces* based on the T92 + G model of DNA substitution; bootstrap values less than 50% are not shown. The tree is rooted on a member of *Kitasatospora*. Red font represents the isolate(s) in this study.


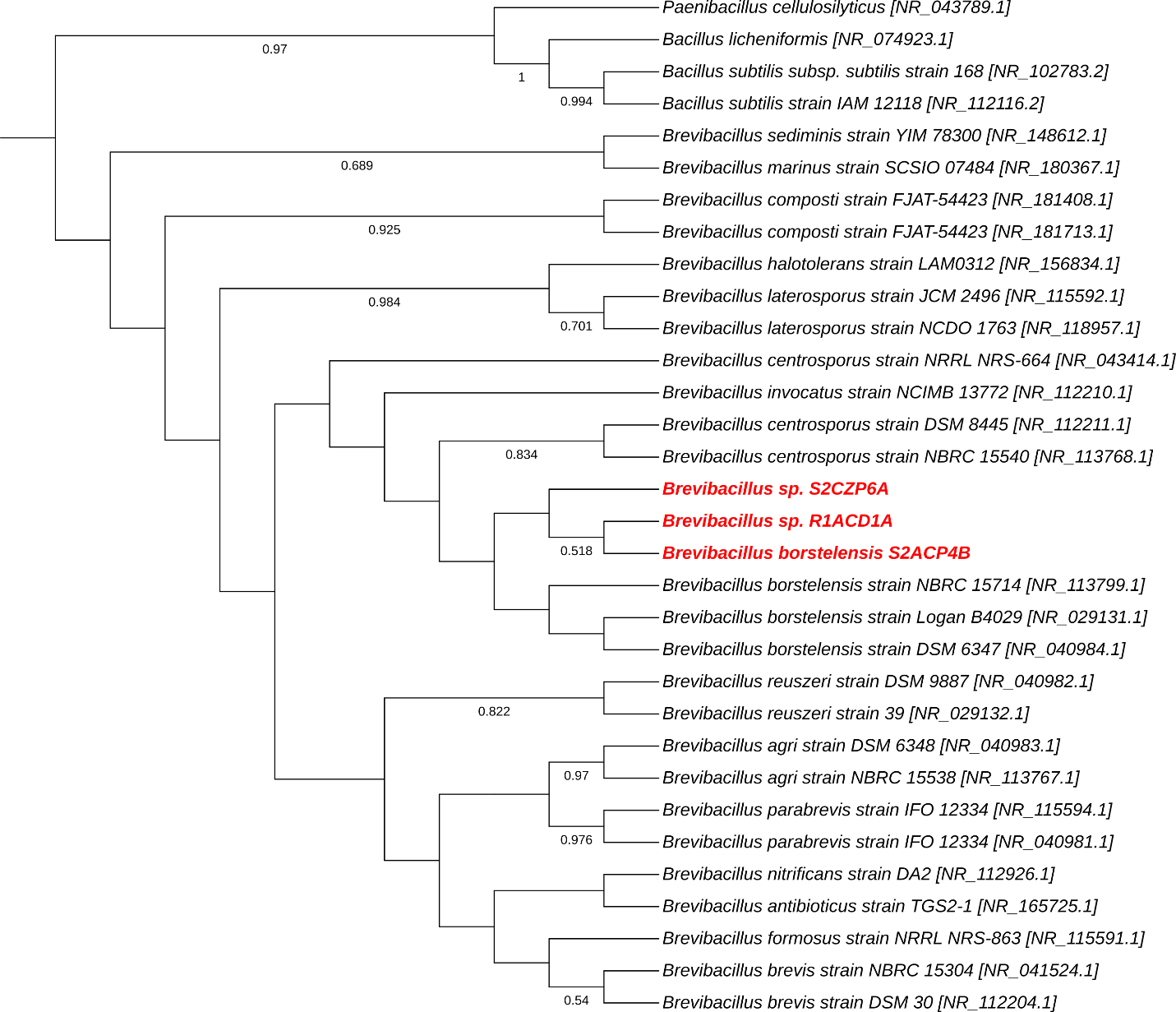


**Fig. S7.** The neighbor-joining phylogenetic tree of the genus *Brevibacillus* based on the T92 + G model of DNA substitution; bootstrap values less than 50% are not shown. The tree is rooted on members of *Bacillus* and *Paenibacillus*. Red font represents the isolate(s) in this study.


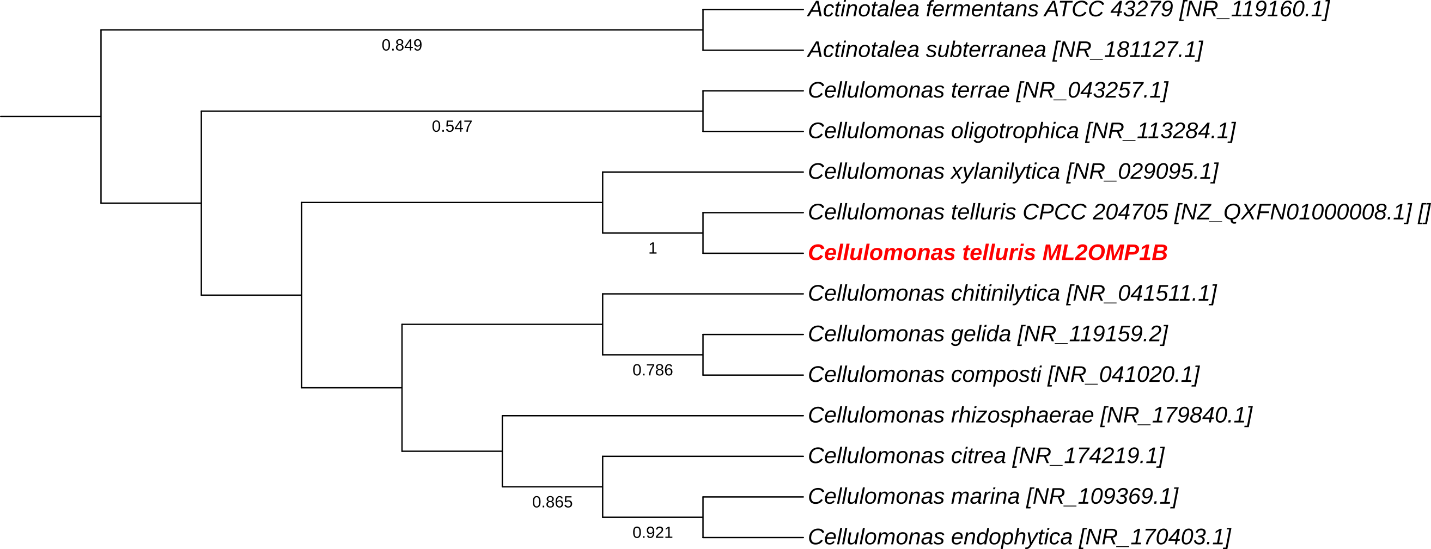


**Fig. S8.** The maximum likelihood phylogenetic tree of the genus *Cellulomonas* based on the K2 + G model of DNA substitution; bootstrap values less than 50% are not shown. The tree is rooted on a member of *Actinotalea*. Red font represents the isolate(s) in this study.


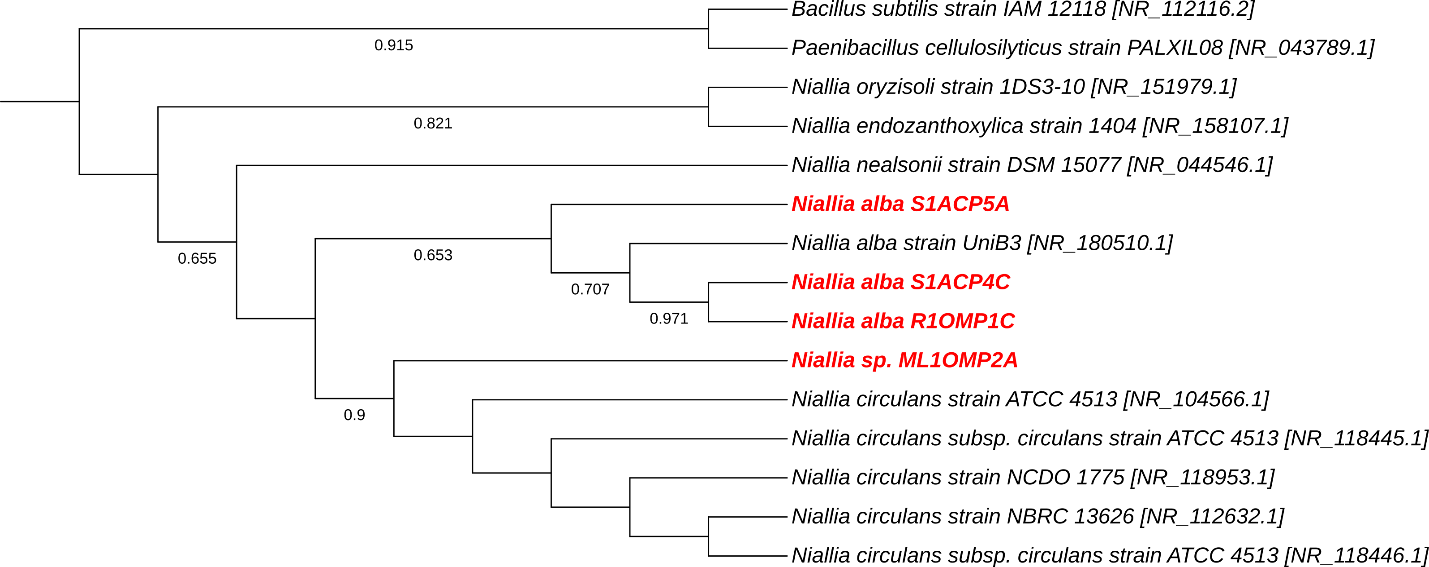


**Fig. S9.** The neighbor-joining phylogenetic tree of the genus *Niallia* based on the T92 + G model of DNA substitution; bootstrap values less than 50% are not shown. The tree is rooted on a member of *Paenibacillus* and *Bacillus*. Red font represents the isolate(s) in this study.


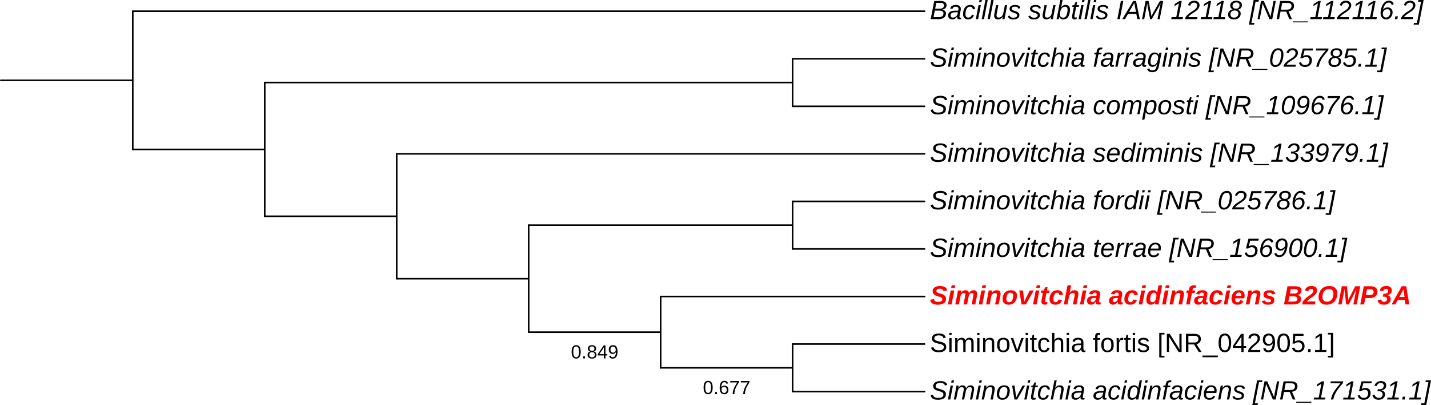


**Fig. S10.** The maximum likelihood phylogenetic tree of the genus *Siminovitchia* based on the T92 + G + I model of DNA substitution; bootstrap values less than 50% are not shown. The tree is rooted on a member of *Bacillus*. Red font represents the isolate(s) in this study.


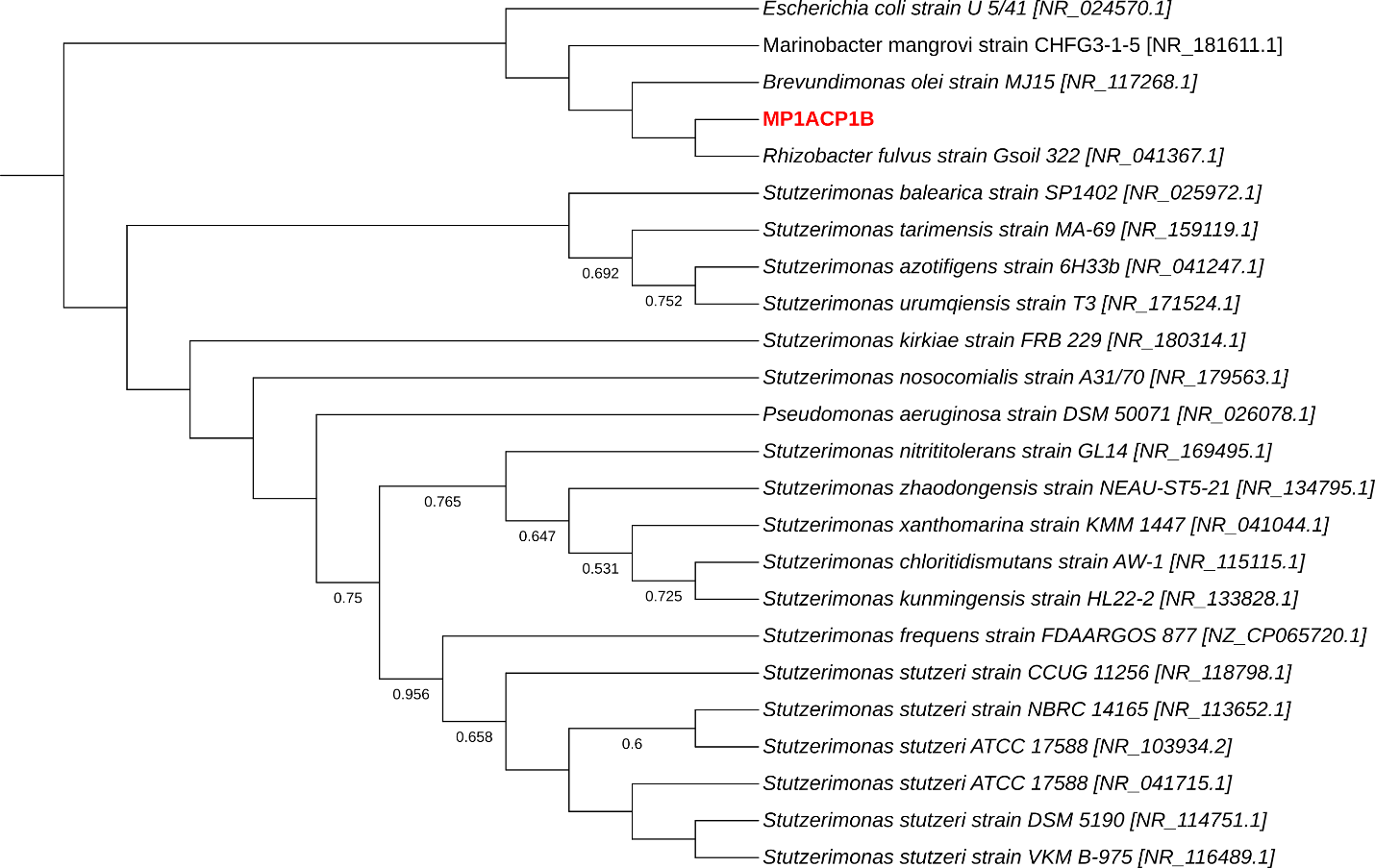


**Fig. S11.** The neighbor-joining phylogenetic tree of the genus *Stutzerimonas* (presumptive identity of MP1ACP1B isolate) based on the K2 + G model of DNA substitution; bootstrap values less than 50% are not shown. The tree is rooted on a member of *Rhizobacter, Brevundimonas, Marinobacter,* and *E. coli*. Red font represents the isolate(s) in this study.


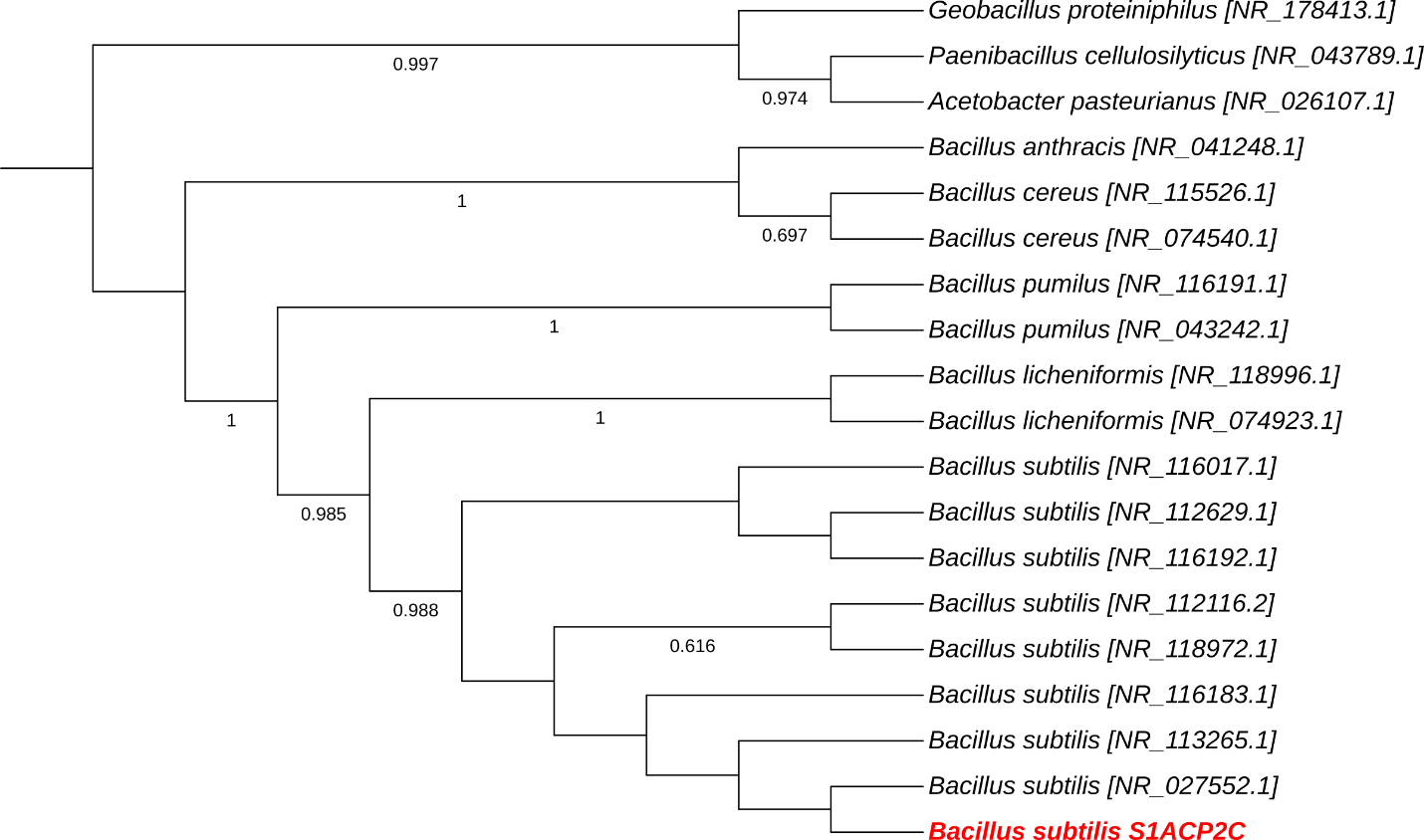


**Fig. S12.** The maximum likelihood phylogenetic tree of the genus *Bacillus* based on the T92 + G model of DNA substitution; bootstrap values less than 50% are not shown. The tree is rooted on members of *Acetobacter*, *Paenibacillus,* and *Geobacillus*. Red font represents the isolate(s) in this study.

**Table S1.** Summary of the soil sample analysis data and sampling sites.

| **Site** | **Coordinates** | **Elevation (m)** | **Humidity**  **(%)** | **Soil Temperature (°C)** | **Soil pH** | **Total Organic Matter (%)** | **Total Nitrogen (%)** | |
| --- | --- | --- | --- | --- | --- | --- | --- | --- |
| Rice Paddy Field, Miagao (RP) | N 10° 39.625’ E 112° 13.686’ | 29 | 76% | 31-32 | 8.05-8.15 | 3.0-3.5 | 0.12-0.17 |  |
| Sugarcane Field in Tulatulaan, Dingle (SC) | N 11° 03.336’ E 122° 42.062’ | 92 | 81% | 27.6 | 5.94-6.22 | 3.3-3.8 | 0.15 |  |
| Bulabog Putian Natural Park, Dingle (BP) | N 11° 01.628’  E 122° 39.532’ | 120 | 80% | 28.6 | 7.74-8.06 | 10.4-27.6 | 0.70-1.23 |  |
| Sibalom Natural Park, Antique (SA) | N 10° 47.320’ E 122° 06.649’ | 134 | 81% | 25.7 | 5.33-6.27 | 3.89-6.18 | 0.13-0.25 |  |
| Pedada Mangrove Sanctuary, Ajuy (MP) | N 11° 03.508’ E 112° 56.975’ | 3 | 80% | 29-30 | 8.45-8.50 | 1.1-1.2 | 0.02-0.03 |  |
| San Dionisio Mangrove Area (MS) | N 11° 15.933’ E 113° 05.720’ | 18 | 74% | 31.5 | 7.74-8.05 | 1.8-6.5 | 0.03-0.17 |  |
| Katunggan Eco Park, Leganes (ML) | N 10° 46.778’ E 112° 37.495’ | 10 | 69% | 28.6 | 5.20-6.17 | 1.0-6.2 | 0.02-0.03 |  |

**Table S2.** Statistical analysis of enzyme assays and CIs among isolates using ANOVA.

| **Enzyme** | Source | **Sum of Squares** | **df** | **Mean Square** | **F-value** | **p-value** |
| --- | --- | --- | --- | --- | --- | --- |
| CMCase | Isolate | 0.1740 | 17 | 0.0102 | 28.2 | < 0.001 |
|  | Error | 0.0131 | 36 | 3.64e-4 |  |  |
| Avicelase | Isolate | 0.2658 | 17 | 0.01563 | 6.03 | < 0.001 |
|  | Error | 0.0933 | 36 | 0.00259 |  |  |
| FPase | Isolate | 0.2302 | 17 | 0.0135 | 28.4 | < 0.001 |
|  | Error | 0.0172 | 36 | 4.77e-4 |  |  |
| Cellulolytic Index | Isolate | 36.63 | 17 | 2.1550 | 28.5 | < 0.001 |
|  | Error | 2.73 | 36 | 0.0757 |  |  |

**Table S3.** Statistical analysis of enzyme assay between the terrestrial and marine environments using ANOVA and Tukey’s test.

| **Enzyme** | Source | **Sum of Squares** | **df** | **Mean Square** | **F-value** | **p-value** | **p_tukey_** |
| --- | --- | --- | --- | --- | --- | --- | --- |
| CMCase | Environment | 0.0471 | 1 | 0.04707 | 17.5 | < 0.001 | <0.001 |
|  | Error | 0.1400 | 52 | 0.00269 |  |  |  |
| Avicelase | Environment | 0.102 | 1 | 0.10243 | 20.8 | < 0.001 | <0.001 |
|  | Error | 0.257 | 52 | 0.00494 |  |  |  |
| FPase | Environment | 8.96e-4 | 1 | 8.96e-4 | 0.189 | 0.666 | 0.666 |
|  | Error | 0.246 | 52 | 0.00474 |  |  |  |
| Cellulolytic Index | Environment | 9.84 | 1 | 9.840 | 17.3 | < 0.001 | <0.001 |
|  | Error | 29.52 | 52 | 0.568 |  |  |  |

**Table S4.** Estimated marginal means of the enzyme assays and cellulolytic index between the two environments.

| **Enzyme** | Environment | **Mean** | **SE** | **95% Confidence Interval** | |
| --- | --- | --- | --- | --- | --- |
|  |  |  |  | **Lower** | **Upper** |
| CMCase | Terrestrial | 0.168 | 0.00865 | 0.150 | 0.185 |
|  | **Marine** | 0.230 | 0.01223 | 0.206 | 0.255 |
| Avicelase | Terrestrial | 0.165 | 0.0117 | 0.142 | 0.189 |
|  | **Marine** | 0.258 | 0.0166 | 0.225 | 0.291 |
| FPase | Terrestrial | 0.207 | 0.0115 | 0.184 | 0.230 |
|  | **Marine** | 0.199 | 0.0162 | 0.166 | 0.231 |
| Cellulolytic Index | Terrestrial | 4.69 | 0.126 | 4.44 | 4.94 |
|  | **Marine** | 3.78 | 0.178 | 3.43 | 4.14 |

**Table S5.** Statistical analysis of pre- and post-optimization of enzyme assay between S1ACP6B and MS1OMP2A isolates using ANOVA and Tukey’s test.

| **Isolate** | **Enzyme** | Source | **Sum of Squares** | **df** | **Mean Square** | **F-value** | **p-value** | **p_tukey_** |
| --- | --- | --- | --- | --- | --- | --- | --- | --- |
| S1ACP6B | CMCase | Factor | 0.0172 | 1 | 0.0172 | 304.03 | <0.001 | <0.001 |
|  |  | Error | **2.26e-4** | 4 | 5.65e-5 |  |  |  |
|  | Avicelase | Factor | 0.0187 | 1 | 0.0187 | 4.43 | 0.103 | 0.103 |
|  |  | Error | 0.0169 | 4 | 0.0042 |  |  |  |
|  | FPase | Factor | 0.0006 | 1 | 0.0006 | 7.34 | 0.054 | 0.054 |
|  |  | Error | 0.0003 | 4 | 0.0001 |  |  |  |
| MS1OMP2A | CMCase | Factor | 0.0001 | 1 | 0.0001 | 0.30 | 0.615 | 0.615 |
|  |  | Error | 0.0007 | 4 | 0.0002 |  |  |  |
|  | Avicelase | Factor | 0.0058 | 1 | 0.0058 | 1.32 | 0.314 | 0.314 |
|  |  | Error | 0.0174 | 4 | 0.0044 |  |  |  |
|  | **FPase** | Factor | 0.0007 | 1 | 0.0007 | 0.74 | 0.437 | 0.437 |
|  |  | Error | 0.0037 | 4 | 0.0009 |  |  |  |

**Table S6.** The molecular identity of the eighteen cellulolytic isolates using 16S rRNA and ITS gene sequences.

| **Habitat** | **Isolate** | **NCBI Scientific Name** | **Query Coverage (%)** | **Percent Identity (%)** | **Accession Number** |  |
| --- | --- | --- | --- | --- | --- | --- |
| Terrestrial | R1OMP1C | *Niallia* *alba* | 98% | 100% | NZ_JABBPK010000001.1 |  |
|  | S1ACP4C | *Niallia alba* | 98% | 100% | NZ_JABBPK010000001.1 |  |
|  | S1ACP5A | *Niallia* *alba* | 89% | 100% | NZ_JABBPK010000001.1 |  |
|  | R1ACD1A | *Brevibacillus composti* | 100% | 98.45% | NZ_CP066308.1 |  |
|  | S2ACP4B | *Brevibacillus borstelensis* | 99% | 99.42% | NR_029131.1 |  |
|  | S1ACP2C | *Bacillus* *subtilis* | 98% | 100% | NC_000964.3 |  |
|  | S1ACP6B | *Enterobacter roggenkampii* | 100% | 99.88% | NZ_CP017184.1 |  |
|  | B2OMP3A | *Siminovitchia acidinfaciens* | 99% | 98.64% | NZ_QYTV02000021.1 |  |
| Marine | MS1OMP2A | *Rhodococcus erythropolis* | 98% | 98% | NR_037024.1 |  |
|  | MP1ACP1B | *Stutzerimonas frequens* | 96% | 100% | NZ_CP065720.1 |  |
|  | ML2OMP1B | *Cellulomonas telluris* | 97% | 99.84% | NZ_QXFN01000008.1 |  |
|  | ML1OMP2A | *Niallia alba* | 97% | 99.67% | NZ_JABBPK010000001.1 |  |
| ***Isolates that need further characterization*** | | | | | | |
| Terrestrial | B1CZP10A | unknown | 86% | 90.56% | PP748307 |  |
| Marine | MS1ACP1B | *Halomonas* sp. | 92% | 96.39% | PP748254 |  |
|  | MP2ACP3B | *Albirhodobacter* sp. | 99% | 94.31% | PP748306 |  |
| ***Isolates with chloramphenicol resistance*** | | | | | | |
| Terrestrial | S2CZP6B | *Streptomyces coacervatus* | 99% | 99.52% | NZ_JARHTP010000059.1 |  |
|  | B1CZP7A | *Streptomyces* *chartreusis* | 98% | 99.46% | NZ_CP023689.1 |  |
|  | S2CZP6A | *Brevibacillus agri* | 98% | 99.92% | NZ_CP026363.1 |  |
